# SARS-CoV-2 infection rates and associated risk factors in healthcare workers: systematic review and meta-analysis

**DOI:** 10.1038/s41598-025-89472-5

**Published:** 2025-02-08

**Authors:** Amit Bansal, Mai-Chi Trieu, Emily M. Eriksson, Fan Zhou, Jodie McVernon, Karl Albert Brokstad, Rebecca Jane Cox

**Affiliations:** 1 Department of Clinical Science, Influenza Centre, University of Bergen, 5020 Bergen, Norway; 2 Department of Infectious Diseases, The Peter Doherty Institute for Infection and Immunity, University of Melbourne, Melbourne, VIC Australia; 3 Norwegian School of Sport Sciences, Oslo, Norway; 4 Department of Microbiology and Immunology, The Peter Doherty Institute for Infection and Immunity, University of Melbourne, Melbourne, VIC Australia; 5 Population Health and Immunity Division, The Walter and Eliza Hall Institute of Medical Research, Melbourne, Australia; 6 Department of Safety, Chemistry and Biomedical Laboratory Sciences, Western Norway University of Applied Sciences, Bergen, Norway; 7 Department of Microbiology, Haukeland University Hospital, Bergen, Norway

**Keywords:** Healthcare workers (HCWs), COVID-19, SARS-CoV-2, Infection, Risk factor, Occupational, Household, Quarantine, Disease prevention, Health policy, Occupational health, Environmental microbiology, Virology, Immunology, Microbiology, Public health, Epidemiology, Risk factors

## Abstract

To protect healthcare workforce during the COVID-19 pandemic, rigorous efforts were made to reduce infection rates among healthcare workers (HCWs), especially prior to vaccine availability. This study aimed to investigate the prevalence of SARS-CoV-2 infections among HCWs and identify potential risk factors associated with transmission. We searched MEDLINE, Embase, and Google Scholar from 1 December 2019 to 5 February 2024. From 498 initial records, 190 articles were reviewed, and 63 studies were eligible. ROBINS-E tool revealed a lower risk of bias in several domains; however, some concerns related to confounding and exposure measurement were identified. Globally, 11% (95% confidence interval (CI) 9–13) of 283,932 HCWs were infected with SARS-CoV-2. Infection rates were associated with a constellation of risk factors and major circulating SARS-CoV-2 variants. Household exposure (odds ratio (OR) 7.07; 95% CI 3.93–12.73), working as a cleaner (OR 2.72; 95% CI 1.39–5.32), occupational exposure (OR 1.79; 95% CI 1.49–2.14), inadequate training on infection prevention and control (OR 1.46; 95% CI 1.14–1.87), insufficient use of personal protective equipment (OR 1.45; 95% CI 1.14–1.84), performing aerosol generating procedures (OR 1.36; 95% CI 1.21–1.52) and inadequate hand hygiene (OR 1.17; 95% CI 0.79–1.73) were associated with an increased SARS-CoV-2 infection. Conversely, history of quarantine (OR 0.23; 95% CI 0.08–0.60) and frequent decontamination of high touch areas (OR 0.52; 95% CI 0.42–0.64) were protective factors against SARS-CoV-2 infection. This study quantifies the substantial global burden of SARS-CoV-2 infection among HCWs. We underscore the urgent need for effective infection prevention and control measures, particularly addressing factors such as household exposure and occupational practices by HCWs, including cleaning staff.

**Supplementary Information:**

The online version contains supplementary material available at 10.1038/s41598-025-89472-5.

## Introduction

There has been an increase in emerging infectious diseases with a pandemic potential in the last decades^1–3^. The most recent COVID-19 pandemic caused significant and widespread morbidity and mortality, prompting changed behaviours^4^. Globally, over 775 million COVID-19 cases have been confirmed as of 18 August 2024, and there have been more than 7 million excess mortalities^5^. Healthcare workers (HCWs) have experienced considerable morbidity and mortality during the pandemic^6^ with an estimated overall global case fatality rate (CFR) of approximately 9 deaths per 1000 infections, alongside an infection rate of 14.5%. However, studies have reported infection rates as high as 59% among HCWs during the COVID-19 pandemic^7–9^. Despite effective national SARS-CoV-2 control measures^7,10,11^, HCWs remain disproportionately susceptible to infection, leading to nosocomial outbreaks that significantly impacted healthcare capacity due to large-scale quarantine.

Viral outbreaks and pandemics pose a continued challenge to healthcare capacity on a global scale. Healthcare institutions need to take preventive measures to limit the spread of infection at both an individual and hospital level. Due to the inherent high transmission potential of SARS-CoV-2^12^, it was among the most common nosocomial adverse events that affect patient safety. Early in the pandemic, public health warnings were put in place globally to stop the SARS-CoV-2 virus from spreading, including physical distancing, frequent handwash, and wearing properly fitted masks. Studies have examined how well these interventions work in lowering SARS-CoV-2 incidence, transmission, and mortality^7,11,13,14^. The infection prevention and control (IPC) policies that aim to curtail the spread of infectious respiratory viruses work best when multi-faceted^7,11,15^.

Personal protective equipment (PPE) use is designed to protect the wearer from hazards, but it is not always effective. PPE can be incorrectly fitted, or not properly maintained and used. Moreover, PPE can restrict mobility or vision, or require the wearer to carry additional weight, which may lead to musculoskeletal problems. As a result, HCWs may not wear PPEs because of discomfort, and they may then be blamed for their own infection. A critical shortage of PPEs emerged globally during the initial months of the COVID-19 pandemic. This necessitated the reuse of single-use PPE, often without established protocols for optimisation or adaptation of procedures. There remains a need for coherent and simple IPC policies with continued viral surveillance to identify infection rates and potential transmission risk factors, particularly during high community spread^16^. Standardising respirator use across wards is crucial for staff protection^16^. Hand hygiene is another important measure for reducing SARS-CoV-2 transmission in healthcare settings. A systematic review published in 2021 found that community hand hygiene interventions were effective in reducing the incidence of SARS-CoV-2 infection^13^. More recently, the presence of SARS-CoV-2 RNA on the hands of primary cases and contacts, as well as on commonly touched household surfaces, was linked to SARS-CoV-2 transmission to household contacts^17^, but not the presence of viral RNA levels in the upper respiratory tracts of primary cases. Notably, this British study did not establish causative relationship^17^. Some COVID-19 pandemic mitigation measures such as quarantine and sick leave^18,19^ negatively impacted healthcare capacity, including individual behavioural changes, such as fear-induced avoidance of workplaces and other public gathering places. Nonetheless, the exact risk profiling of different factors among HCWs is still largely unknown.

In this systematic review and meta-analysis, we took advantage of the considerable global SARS-CoV-2 infection data among HCWs and quantified the SARS-CoV-2 infection rates, thus determining the potential risk factors for infection in HCWs. We evaluated which non-pharmacological variables reduced the spread of SARS-CoV-2 in healthcare settings, focusing on exposure elimination, usage of PPE, and hospital environmental services. The findings of this review provide insights into SARS-CoV-2 transmission in healthcare settings and will aid in hospital preparedness for emerging respiratory viral outbreaks/epidemics and pandemics, particularly before vaccines are available.

## Methods

This is a systematic review and meta-analyses on infection rates, as well as on analytic observational studies for assessing potential risk factors for SARS-CoV-2 infection among HCWs from a wide range of countries. We included anonymous data that were extracted from previously published studies. All studies included in this review received ethical approval from their local review boards, where required, and informed consent from participants were obtained by the studies’ investigators. We followed the reporting guidelines^20^ set forth by Preferred Reporting Items for Systematic Reviews and Meta-analyses (PRISMA).

### Design

We conducted a systematic review to determine global SARS-CoV-2 infection rates and identify key risk or protective factors associated with SARS-CoV-2 infection among HCWs. Eligible studies were published between 1 December 2019 and 5 February 2024. SARS-CoV-2 transmission risk characterises the likelihood of an infected person transmitting the virus to a susceptible person through various routes (through-the-air at work/home and direct contact), and activities such as aerosol generating procedures, PPE use, decontamination of high touch areas, working in environmental services and hand hygiene. This review examines transmission risk factors, including occupational and household exposure to SARS-CoV-2, and behaviours or activities listed above. Transmission risk and infection or susceptibility risk are related. While susceptibility risk is broader and includes factors like age and prior immunity, this review focuses on the aspects influencing transmission among HCWs.

### Search strategy

We selected three electronic databases for study identification: MEDLINE, Embase, and Google Scholar. Inclusion and exclusion criteria limited the search to human participants, English language, and selected article type (clinical trials, observational study, letters, case reports) (Supplementary Tables 1–4). Combining MEDLINE, Embase, and Google Scholar for meta-analyses provided comprehensive access to high-quality, peer-reviewed biomedical literature, thereby enhancing our review’s robustness and reliability. Our main outcome measure was SARS-CoV-2 infection (determined by reverse transcription polymerase chain reaction (RT-PCR) test, nucleoprotein seropositivity, or spike-protein seropositivity in unvaccinated individuals, and not relying solely on symptoms). Search terms included SARS-CoV-2 infection rates, risk assessment, occupational exposure to SARS-CoV-2, household exposure to SARS-CoV-2, masking, infection prevention control training, hand hygiene, environmental/hospital infection control**,** COVID-19, COVID, coronavirus, Wuhan, 2019, SARS, SARS-CoV-2, coronavirus 2, 2019-ncov, SARS-2, health worker, health workforce, health professional, nurse, sero-epidemiologic studies, seroprevalence, antibodies, serological tests, risk factors, immunoglobulin, public health intervention, masking, and hand washing in MEDLINE and Embase databases (Supplementary Tables 2 and 3). For Google Scholar, we conducted a search using relevant keywords, without specific concept groups, limiting our results to the top 251 studies to ensure a comprehensive yet manageable dataset and avoiding overly extensive searches that yield unnecessary results (Supplementary Table 4).

### Data collection process

Studies were first screened by titles and abstracts according to the inclusion and exclusion criteria (Supplementary Table 1). The eligible studies were thoroughly reviewed, and relevant data were initially extracted by the first author. The number of cases of SARS-CoV-2 infection and total subgroup size in connection with exposure variables were extracted as 2 × 2 table data from the existing global HCW literature. Two authors independently reviewed the papers, extracted data, and assessed the risk of bias using the risk of bias in non-randomized follow-up studies of exposure effects (ROBINS-E) and Risk-of-bias VISualization (robvis) tools^21^ (Supplementary Fig. 1). Regular meetings were held to discuss the included articles, cross-check findings, and minimise errors and potential biases. The initial rigorous review process conducted by the first author resulted in a good database, leading to minimal discrepancies during review by the second reviewer. The majority of included studies demonstrated sound scientific rigour, albeit we needed to contact some article’s authors for clarification or more information. The ROBINS-E tool revealed a low risk of bias in several domains; however, some concerns emerged regarding confounding and exposure measurement. None of the studies exhibited severe flaws necessitating exclusion, and there were no substantial concerns regarding study quality.

### Statistics of meta-analyses

Quantitative analyses of included studies were performed using both fixed and random-effects models using the metaprop and metabin functions in R version 4.4.1 (meta package^22^). Pooled effect estimates with corresponding 95% confidence intervals (CI) were reported. Forest plots displayed infection rates or odds ratios (ORs) with 95% CIs, accompanied by total case numbers and study population sizes. Heterogeneity among studies was assessed using I^2^ test statistic, with two-tailed *p* values. The I^2^ statistic quantifies the proportion of total variation in effect estimates across studies that is attributable to heterogeneity rather than chance alone^23^. The I^2^ values of 0–25%, 26–50%, and above 50% were considered to represent low, moderate, and high levels of heterogeneity, respectively. However, these thresholds are arbitrary and should be interpreted with caution. For each potential risk factor, both fixed and random effect models were conducted. Fixed-effects models assume a common treatment effect across all studies, with observed differences attributed solely to chance. Study weights are assigned based on the inverse variance of study effect sizes. Random-effects models account for both chance and between-study heterogeneity. Study weights are determined by the inverse of their effect size variances, with an added term accounting for between-study heterogeneity. The random-effects models were preferred to pool the prevalence or risk of infection since each study was conducted in a different population with differing effect sizes. Studies were subgrouped, where appropriate, based upon major circulating variant of concerns (VOCs). For each study, the circulating VOCs were determined by inputting the study period and country in nextstrain.org website and using virological data sourced from GISAID public repositories (www.gisaid.org)^24^. Funnel and Doi plots were used to assess publication bias with visual inspection because funnel plot alone may be inaccurate particularly for prevalence or infection rate estimates^25^. Doi plots include standardised score against effect size and Luis Furuya-Kanamori (LFK) index^26^ (computed using metasens package^27^). Potential publication bias is indicated by LFK index values outside the range of − 1 to + 1. We used the trim-and-fill method (trimfill function in R) to address potential publication bias in asymmetrical funnel plots and/or LFK values outside the range − 1 to + 1. This method trims outliers, adjusts the summary effect, and fills in missing studies. It is a sensitivity analysis and assumes publication bias as the sole cause of asymmetry.

### Registration

This systematic review project was registered on Open Science Framework (OSF) on 21-12-2023 (10.17605/OSF.IO/EFMTA).

## Results: risk factors and role of public health interventions in HCWs

We investigated the prevalence and risk factors for SARS-CoV-2 infection, which included occupational exposure to SARS-CoV-2, inadequate IPC training, insufficient use of PPE at work, performing aerosol-generating procedures, hygiene measures, quarantine and household exposure.

### Study selection

**Fig. 1:**
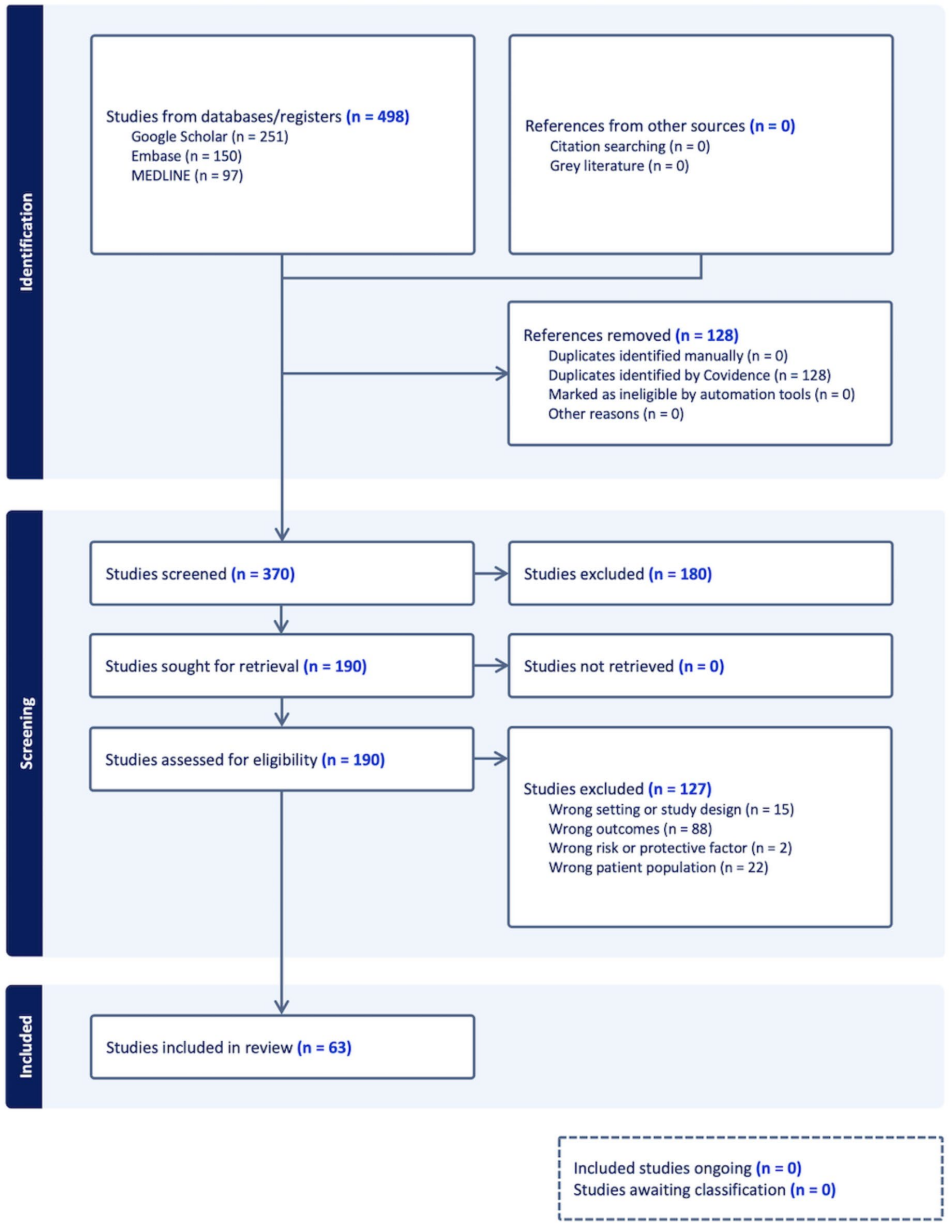
PRISMA Flow Diagram for selection of the studies in systematic review and meta-analysis. Figure was created using Covidence. Covidence is a web-based collaboration software platform that streamlines the production of systematic and other literature reviews. This study aimed to identify key risk and protective factors associated with SARS-CoV-2 infection among healthcare workers (HCWs). The included quantitative studies were published between 1 December 2019 and 5 February 2024 and investigated the association between risk factors and SARS-CoV-2 infection in HCWs. Three electronic databases, MEDLINE, Embase, and Google Scholar, were searched. Inclusion criteria restricted the search to studies involving human participants, published in English, and of specific article types (clinical trials, observational studies, letters, and case reports) (See Table 1 for details). Studies excluded due to “wrong setting” encompassed those with irrelevant characteristics or inappropriate design. The primary outcome measure was SARS-CoV-2 infection, confirmed by either a positive Reverse Transcription Polymerase Chain Reaction (RT-PCR) test, nucleoprotein seropositivity, or spike-protein seropositivity in unvaccinated individuals.

We used Covidence to streamline the production of the systematic review (Covidence systematic review software, Veritas Health Innovation, Melbourne, Australia. Available at www.covidence.org). The search from three databases (MEDLINE, Embase and Google Scholar) yielded a total of 498 studies, of which 370 studies were screened through titles and abstracts (Fig. 1). A total of 190 studies were thoroughly reviewed in full text. Of these, 63 studies met the inclusion criteria and were included in these meta-analyses (see summary of each study in Supplementary Table 5). Data from these studies were collected during December 2019 to December 2021.

### SARS-CoV-2 infection rates

**Fig. 2:**
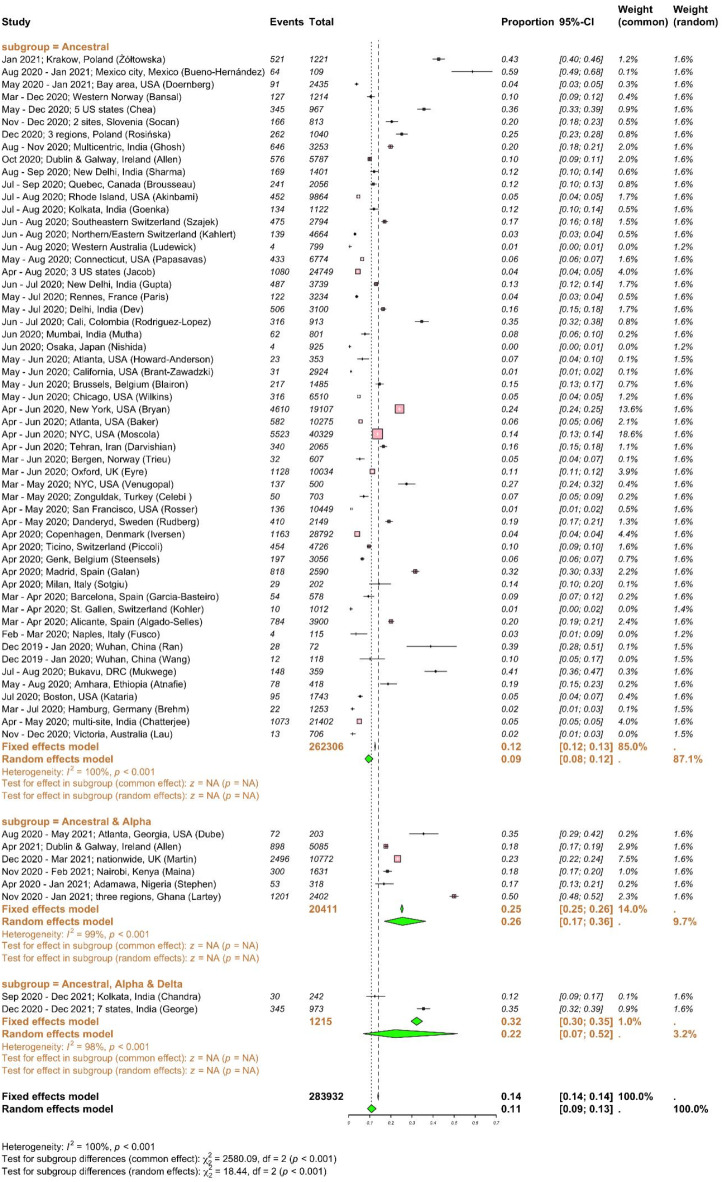
SARS-CoV-2 infection rates among healthcare workers. Meta-analysis of effect estimates was performed using the metaprop function (meta^22^ package) in R version 4.4.1. We used “Plogit” method or logit transformation for pooling of studies. SARS-CoV-2 infection rates with 95% confidence intervals (CIs) among HCWs were reported from 27 countries, including: Australia, Belgium, Canada, China, Colombia, Denmark, Democratic Republic of the Congo (DRC), Ethiopia, France, Germany, India, Iran, Ireland, Italy, Japan, Kenya, Mexico, Nigeria, Norway, Poland, Slovenia, Spain, Sweden, Switzerland, Turkey, the UK and the USA. Infection rates were plotted against major circulating variants of concerns as sub-group analysis. I^2^ is a statistic used in meta-analysis to estimate the proportion of total variance in effect sizes due to heterogeneity between studies. Diamonds representing meta-analysis results are printed in green colour and squares representing individual study results are printed in pink colour. A chi-square test (χ^2^) with degrees of freedom (df) and a *p*-value was conducted to assess differences between subgroups, indicating whether significant differences exist in the effects across subgroups (*p* < 0.05).

All the included studies predominantly focused on HCWs without prior infection or those with a likely primary infection, as opposed to those with breakthrough infection. The SARS-CoV-2 infection rates in HCWs varied widely (ranging between 1 and 59%) in different parts of the world with different transmission settings^7,9,28^. SARS-CoV-2 infection rates with 95% CI among HCWs were reported from 27 countries (Fig. 2). Here, SARS-CoV-2 infection refers to laboratory-confirmed cases, diagnosed through either RT-PCR or serological testing. Globally, SARS-CoV-2 infection rate of 11% (95% CI 9–13%; I^2^ = 100%, high heterogeneity) was found among 283,932 HCWs (Table 1). Since the high heterogeneity between studies could be due to the different infection rates when VOCs involved, we performed a subgroup analysis of infection rate according to the major circulating strains. Majority of included studies (55/63) were conducted between December 2019 and January 2021 (262,306 HCWs), when the ancestral strain circulated. Six studies collected data up to May 2021 (20,411 HCWs), when the alpha VOC emerged and became predominant. Only 2 studies collected data up to December 2021 (1,215 HCWs), when the delta VOC presented and became predominant. SARS-CoV-2 infection rates among HCWs increased from 9% when the ancestral strain was solely presented to 26% and 22% when the alpha and delta strains emerged. The difference in infection rates between subgroups was statistically significant (*p* < 0.001), suggesting that the appearance of VOCs increased the risk of infection among HCWs. However, since far more studies (and participants) contribute data to the ancestral subgroup than to the ancestral and alpha subgroup or ancestral, alpha and delta VOC sub-group, these results should be interpreted with caution. We found a wider range of infection rates in the USA^29,30^ (1–35%) and India^31,32^ (5–20%) in 2020–21. The highest infection rates among HCWs reported in Mexico^9^ was 59% (95% CI 49–68%) during August 2020–January 2021, 43% (95% CI 40–46%) in Poland^33^ in January 2021, and 41% (95% CI 36–47%) in the Democratic Republic of the Congo (DRC)^8^ during July–August 2020. In contrast, Australia^34^, Germany^35^, Japan^36^ and Switzerland^37^ had the lowest SARS-CoV-2 infection rates (< 2%) in 2020. We conducted additional subgroup analyses based on geography and study design to identify key contributors to heterogeneity (Supplementary Figs. 2 and 3). The infection rates were significantly impacted by regions: 27% (95% CI 15–45%) in Africa, 12% (95% CI 8–17%) in Asia, 10% (95% CI 8–14%) in Europe, 9% (95% CI 6–13%) in North America, 1% (95% CI 0–4%) in Oceania, and 1% (95% CI 0–4%) in South America (Supplementary Fig. 2). Additionally, infection rates differed by study design, although not significantly; 22% (95% CI 9–44%) for case–control studies, 8% (95% CI 6–11%) for cohort studies and 11% (95% CI 8–13%) for cross-sectional studies (Supplementary Fig. 3). The heterogeneity was high for all subgroup analyses, suggesting multiple contributing risk factors. Publication bias was implied in both funnel and Doi plots (LFK index = − 2; Supplementary Fig. 4a,b). Despite potential evidence of publication bias, visual asymmetry in the funnel plot and false positive LFK index may also arise from small study effects and/or true heterogeneity between the included studies^38,39^. As this list of studies demonstrates, there is a significant over-representation of research from high-income nations. There is limited data from South America, Africa, and the Middle East, which is likely related to inadequate surveillance in less developed countries and linguistic barriers. In order to obtain LFK index within acceptable limits, we removed 7 studies based on high standard error of effect estimate (Supplementary Fig. 4c–e). In this subset analyses, the overall infection rate remains comparable for the alpha and delta variants. However, it is 2% higher for the ancestral strain or when subgroup analysis is not performed. The SARS-CoV-2 infection rate among HCWs was estimated to be 14.33% (95% CI 11.95–17.10%) based on 72 studies, with 9 studies added using the trim-and-fill method to account for publication bias (Table 2). There was substantial heterogeneity across studies (I^2^ = 99.6%).

**Table 1:** Key questions and findings.

What is already known?	Healthcare workers (HCWs) are more likely to get SARS-CoV-2 infection than other individuals, even though they are likely well trained in IPC policies
What is unknown?	The precise SARS-CoV-2 infection rate and risk assessment of occupational and household exposure to SARS-CoV-2, masking, hand hygiene, quarantine, and environmental cleaning in controlling outbreaks/pandemics, especially before vaccines are available
What are the new findings?	a) Global SARS-CoV-2 infection rate among 283,932 HCWs was 11% (95% CI 9–13%) b) Infection rates varied by circulating SARS-CoV-2 strain(s): *Ancestral strain: 9% (95% CI 8–12%) *Ancestral and alpha strains combined: 26% (95% CI 17–36%) *Ancestral, alpha, and delta strains combined: 22% (95% CI 7–52%) c) SARS-CoV-2 infection among HCWs was associated with household exposure (OR 7.07; 3.93–12.73), occupational exposure (OR 1.79; 95% CI 1.49–2.14), inadequate IPC training (OR 1.46; 1.14–1.87), insufficient use of PPE (OR 1.45; 1.14–1.84), performing aerosol generating procedures (OR 1.36; 1.21–1.52), inadequate hand hygiene (OR 1.17; 0.79–1.73) and working as a cleaner (OR 2.72; 1.39–5.32). ORs for insufficient PPE use were higher for ancestral strain than combined ancestral and alpha strains, or combined ancestral, alpha and delta strains; suggesting either early shortage of medical supplies, or later efficient interventional measures were taken or both
What do the new findings imply?	The findings indicate a substantial SARS-CoV-2 infection rate among HCWs, with variations in infection risk linked to specific SARS-CoV-2 strains and a constellation of risk factors While household exposure emerged as the major risk factor for SARS-CoV-2 infection, known occupational practices such as improper IPC training, PPE use, and aerosol-generating procedures also contributed to infection risk The observed association between infection rate and SARS-CoV-2 variants suggests that the evolving nature of the virus may influence the effectiveness of prevention measures

**Table 2:** Meta-analyses of SARS-CoV-2 infection rate and potential risk factors for SARS-CoV-2 infection among healthcare workers: summary results of standard and trim-and-fill methods.

Meta-analyses type	Standard method with eligible studies^1^			Trim-and-fill method adjusted estimates^2^		
	Rate/ORs (95% CI)	n	LFK index	Rate/ORs (95% CI)	n	LFK index
Infection rate	10.78% (8.89–13.01%)	63	-2.02	14.33% (11.95–17.10%)	72	− 0.04
Occupational exposure	1.79 (1.49–2.14)	56	-2.04	4.83 (3.45–6.75)	84	0.79
Insufficient PPE	1.45 (1.14–1.84)	28	0.15	–	–	–
Inadequate IPC training	1.46 (1.14–1.87)	7	-0.37	–	–	–
Performing aerosol-generating procedures	1.36 (1.21–1.52)	12	-2.92	1.43 (1.24–1.66)	15	-0.37
HCW working as a cleaner	2.72 (1.39–5.32)	11	0.61	–	–	–
Frequent decontamination of high-touch areas	0.52 (0.42–0.64)	2	3.62	Not applicable*	Not applicable*	Not applicable*
Inadequate hand hygiene	1.17 (0.79–1.73)	10	5.73	0.96 (0.60–1.52)	13	1.03
History of quarantine	0.23 (0.08–0.60)	5	5.27	0.07 (0.02–0.25)	8	0.51
Household exposure to SARS-CoV-2	7.07 (3.93–12.73)	15	3.24	2.76 (1.24–6.14)	22	0.54

^1^Infection rates/odds ratio (OR) and LFK index computed for original dataset using metaprop (for proportions), metabin (for ORs) and doiplot (for LFK index) functions in R.

^2^Infection rates/odds ratio (OR) and LFK index computed after trim-and-fill method to adjust for potential publication bias with additional studies using trimfill and doiplot function in R in case LFK index lies outside the range of − 1 to + 1.

–Not performed as LFK values were within the acceptable range with the standard method.

*Not applicable due to the minimal number of three studies for trim-and-fill method.

### Occupational exposure to SARS-CoV-2

**Fig. 3:**
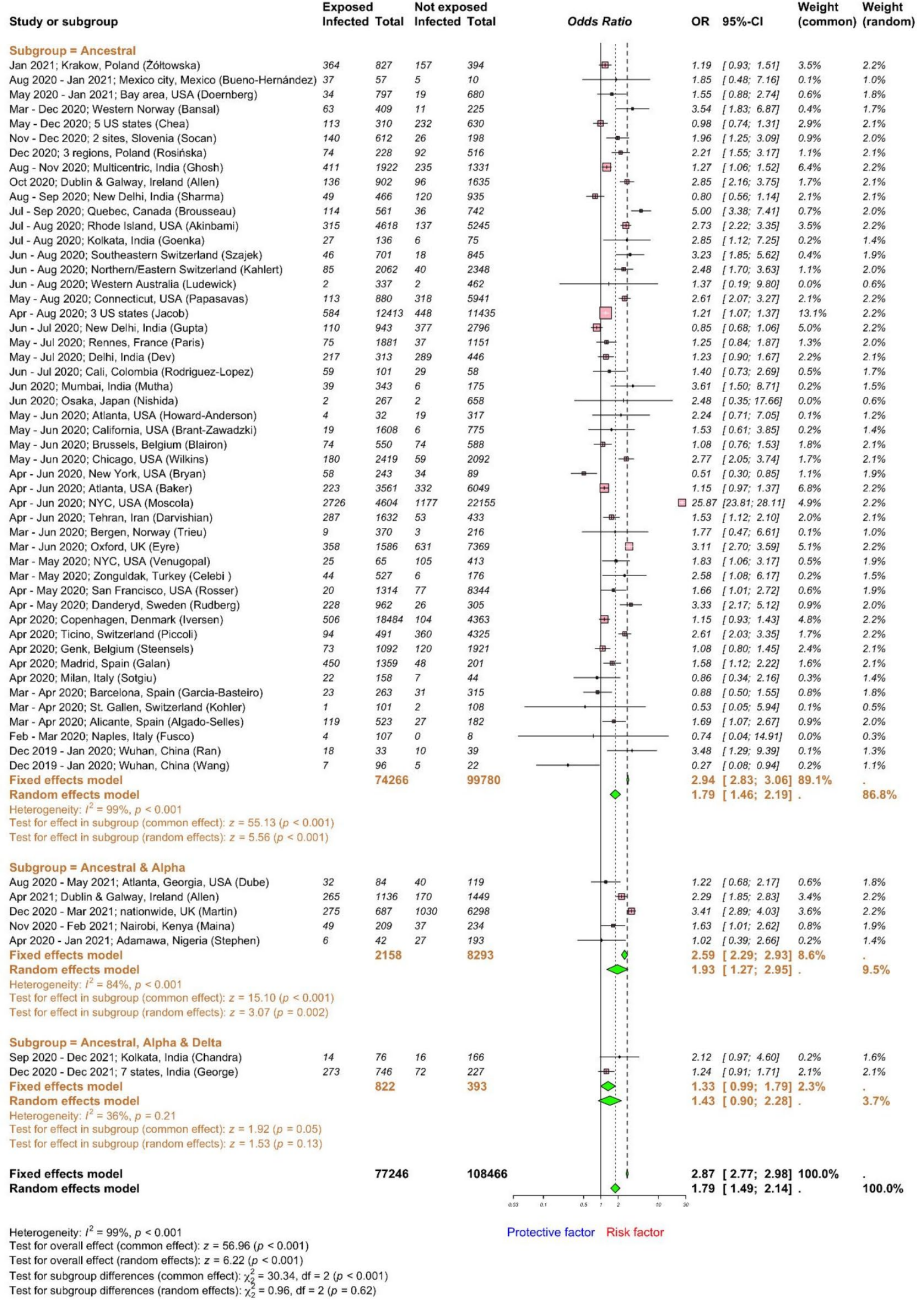
Forest plot to evaluate whether SARS-CoV-2 infection rates differed with occupational exposure to SARS-CoV-2 among healthcare workers. Data are presented as proportions and 95% confidence interval, CI. Meta-analysis of effect estimates was performed using the metabin function (meta^22^ package) in R version 4. 4.1. Pooling of studies was performed using Mantel–Haenszel method. Diamonds representing meta-analysis results are printed in green colour and squares representing individual study results are printed in pink colour. Forest plot was plotted against major circulating variants of concern as sub-group analysis. I^2^ is a statistic used in meta-analysis to estimate the proportion of total variance in effect sizes due to heterogeneity between studies. The z-score for the overall combined subgroup effect (common effect) evaluates the pooled effect size across all subgroups, while the z-score for the overall combined subgroup effect (random effects) considers both within-subgroup and between-subgroup variability. A chi-square test (χ^2^) with degrees of freedom (df) and a *p*-value was conducted to assess differences between subgroups, indicating whether significant differences exist in the effects across subgroups (*p* < 0.05).

We deducted data for occupational exposure from 56 studies including 185,712 HCWs (Fig. 3). High-risk occupational exposure for HCWs was defined as direct patient care that likely exposed them to individuals with COVID-19, such as HCWs working in infectious disease wards. Conversely, HCWs with minimal or no contact with COVID-19 cases, like those in the administration or other designated low-risk areas, were considered at low-risk for occupational exposure. The global pooled OR for SARS-CoV-2 infection against high-risk occupational exposure was 1.79 (95% CI 1.49–2.14; high heterogeneity, I^2^ = 99%) compared to low-risk exposure; meaning that HCWs with high risk of occupational exposure had nearly twice higher odds to be infected with SARS-CoV-2 than HCWs with limited or no occupational exposure. Subgroup analysis based on the major circulating viruses shown that the pooled OR for SARS-CoV-2 infection associated with high-risk occupational exposure was 1.79 (95% CI 1.46–2.19) when the ancestral strain circulated, 1.93 (95% CI 1.27–2.95) when ancestral and alpha strains circulated, and 1.43 (95% CI 0.90–2.28) when ancestral, alpha and delta strains circulated. Low and insignificant heterogeneity was observed within the ancestral, alpha and delta subgroup only. However, the test for VOC subgroup differences indicates that there is no statistically significant subgroup effect (*p* = 0.62). The ancestral subgroup contributes more studies and participants than other subgroups. We conducted additional subgroup analyses based on geography and study design to explore the heterogeneity among studies assessing occupational exposure to SARS-CoV-2 among HCWs. The ORs by regions were as follows: 1.49 (95% CI 0.97–2.28) in Africa, 1.41 (95% CI 1.05–1.88) in Asia, 1.88 (95% CI 1.54–2.30) in Europe, 2.03 (95% CI 1.29–3.19) in North America, 1.37 (95% CI 0.19–9.80) in Oceania, and 1.40 (95% CI 0.73–2.69) in South America (Supplementary Fig. 5). No significant difference in ORs for occupational exposure by regions was observed. However, the ORs were significantly impacted by study design, where cohort studies (OR 2.10 (95% CI 1.29–3.41)) found higher ORs for occupational exposure than cross-sectional (OR 1.70 (95% CI 1.42–2.04)) and case–control (OR 1.18 (95% CI 1.00–1.40)) studies (Supplementary Fig. 6). Publication bias was implied in both funnel and Doi plots with LFK index of − 2 (Supplementary Fig. 7); however, it could also be indicative of small study effects and/or heterogeneity between studies^38,39^. The OR of SARS-CoV-2 infection after occupational exposure for HCWs was estimated to be 4.83 (95% CI 3.45–6.75) based on 84 studies, with 28 studies added using the trim-and-fill method to account for publication bias, with a total of 272,364 observations (127,891 exposed and 144,473 control) and 24,203 events. There was high heterogeneity across studies (I^2^ = 99.0%). In a real-world setting, clinical input would be necessary to determine whether the OR distribution is an important consideration when interpreting this subgroup analysis. Although community infection spread and patient contacts play a key role in occupational exposure, the chance of being infected is influenced by other important work-related factors such as IPC training and practices, PPE use, and working tasks. Hence, this estimate of infection risk for occupational exposure requires further assessment.

### Work-related factors

**Fig. 4:**
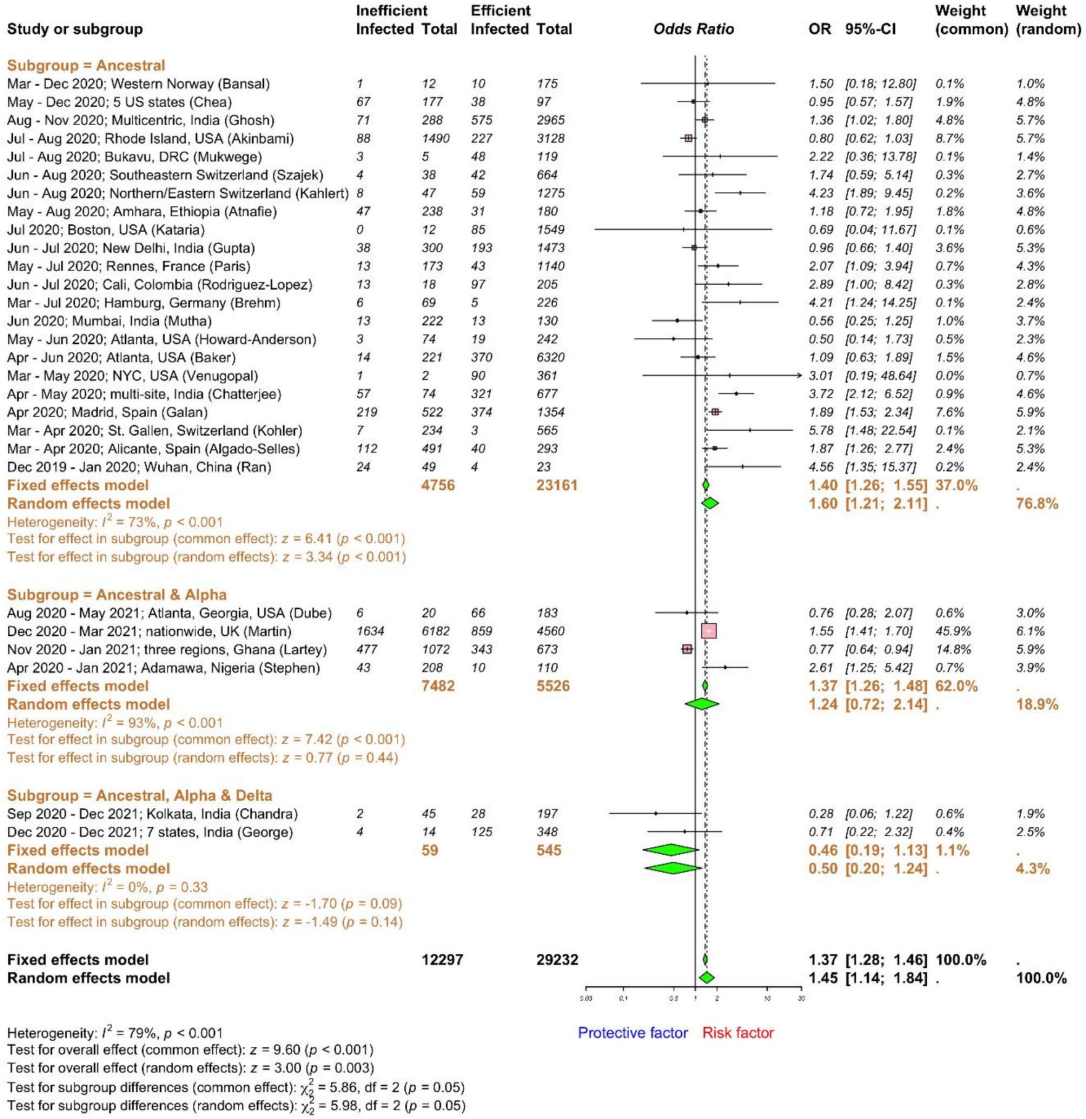
Forest plot to evaluate whether SARS-CoV-2 infection rates differed with personal protective equipment (PPE) use among healthcare workers. Data are presented as odds ratios, ORs, and 95% confidence interval, CI. Meta-analysis of effect estimates was performed using the metabin function (meta^22^ package) in R version 4. 4.1. Pooling of studies was performed using Mantel–Haenszel method. Diamonds representing meta-analysis results are printed in green colour and squares representing individual study results are printed in pink colour. I^2^ is a statistic used in meta-analysis to estimate the proportion of total variance in effect sizes due to heterogeneity between studies. The z-score for the overall combined subgroup effect (common effect) evaluates the pooled effect size across all subgroups, while the z-score for the overall combined subgroup effect (random effects) considers both within-subgroup and between-subgroup variability. A chi-square test (χ^2^) with degrees of freedom (df) and a *p*-value was conducted to assess differences between subgroups, indicating whether significant differences exist in the effects across subgroups (*p* < 0.05).

**Fig. 5:**
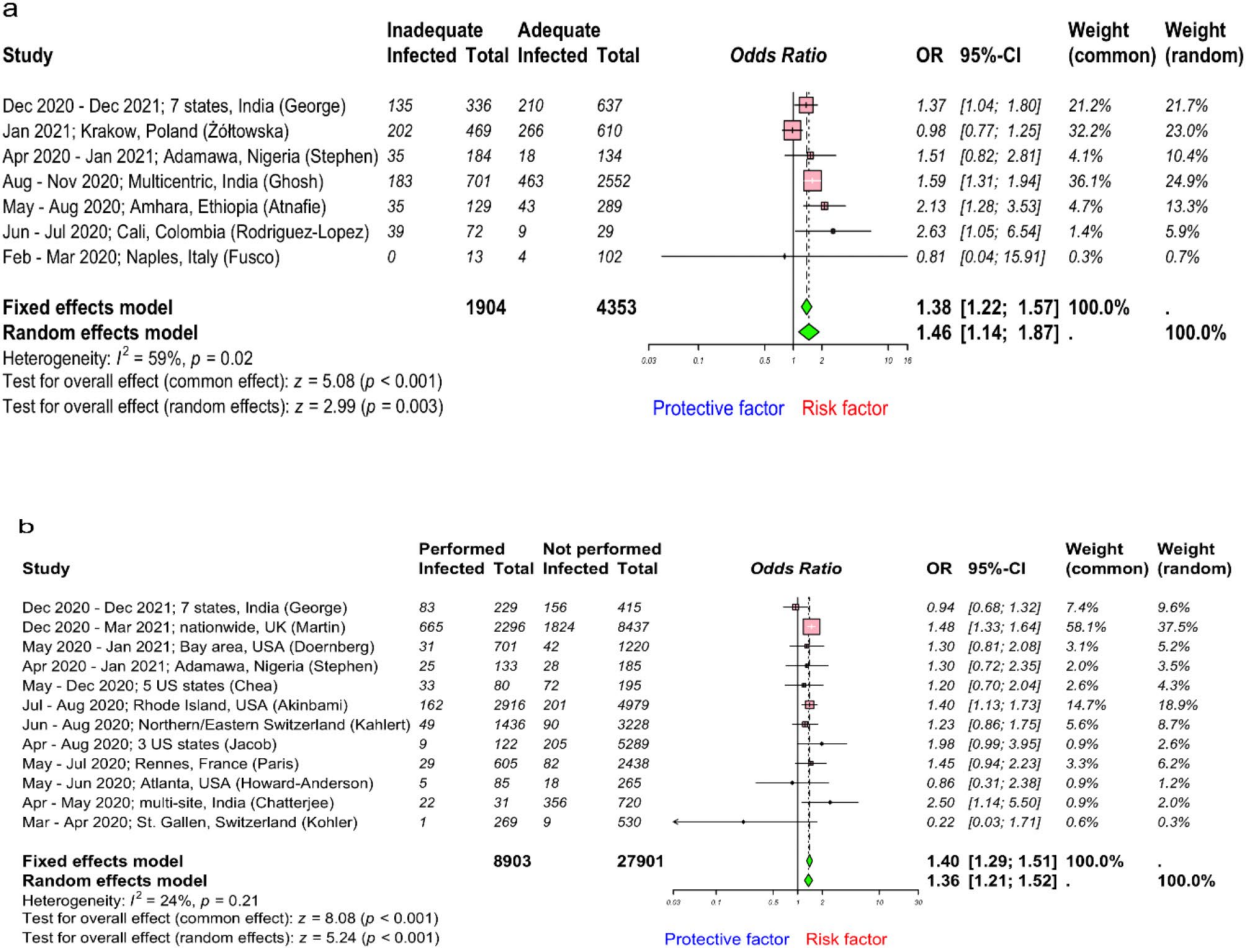
Forest plot to evaluate whether SARS-CoV-2 infection rates differed with infection prevention and control (IPC) training and performing aerosol-generating procedure among healthcare workers. (**a**) and (**b**) describes risk assessment following IPC training and performing aerosol-generating procedures respectively. Data are presented as odds ratios, ORs; and 95% confidence interval, CI. Meta-analysis of effect estimates was performed using the metabin function (meta^22^ package) in R version 4. 4.1. Pooling of studies was performed using Mantel–Haenszel method. Diamonds representing meta-analysis results are printed in green colour and squares representing individual study results are printed in pink colour. I^2^ is a statistic used in meta-analysis to estimate the proportion of total variance in effect sizes due to heterogeneity between studies.

In healthcare settings, various occupational practices can pose risk or protect against SARS-CoV-2 infection. The use of PPE, as appropriate, when in contact with patients or performing certain procedures is often included in IPC training for HCWs. In practice, PPE may not be efficiently used. We retrieved data for occupational exposure with insufficient PPE use from 28 studies^8,30–32,35,37,40–59^ including a total of 41,529 HCWs (Fig. 4). In most studies, “insufficient PPE” encompasses two key areas: neglecting to wear PPE as recommended and/or utilising the incorrect or malfunctioning type of PPE for the given hazard. Subgroup analyses according to the major circulating strains revealed that insufficient PPE use was related to higher odds of SARS-CoV-2 infection with OR of 1.60 (95% CI 1.21–2.11) when ancestral strain predominantly circulated, 1.24 (95% CI 0.72–2.14) when ancestral and alpha circulated, and 0.50 (95% CI 0.20–1.24) when ancestral, alpha and delta strains circulated. No statistically significant differences among VOC subgroups were found (*p* = 0.05). However, it is unlikely to produce clinically useful findings due to the substantial disparity in sample sizes (23,161 HCWs for the ancestral subgroup vs. 5526 HCWs for the ancestral and alpha subgroup and 545 HCWs for the ancestral, alpha, and delta VOC subgroup). To identify key contributors to heterogeneity, we conducted subgroup analyses by geography and study design (Supplementary Figs. 8 and 9). Insufficient PPE use was associated with increased infection odds in Europe (OR 1.95, 95% CI 1.57–2.41) and South America (OR 2.89, 95% CI 1.00–8.42). Associations were less consistent in Africa (OR 1.30, 95% CI 0.70–2.39), Asia (OR 1.21, 95% CI 0.63–2.34), and North America (OR 0.85, 95% CI 0.69–1.04). Cohort studies (OR 2.29, 95% CI 1.34–3.89) showed a stronger association with insufficient PPE use compared to case–control (OR 1.39, 95% CI 0.70–2.78) or cross-sectional studies (OR 1.30, 95% CI 1.00–1.69). The pooled OR for occupational exposure with insufficient PPE use was 1.45 (95% CI 1.14–1.84; I^2^ = 79%, high heterogeneity). Funnel and Doi plots did not reveal substantial publication bias (LFK index = 0.2; Supplementary Fig. 10) in this meta-analysis. Hospitals often provide IPC training for HCWs. The extent, the duration and the frequency of training vary largely by hospital department and between hospitals, regions, and nations, depending on the availability of resources. During the COVID-19 pandemic, inadequate IPC training encompassed insufficient training in various ways such as depth, duration and resources available. Our meta-analysis showed that the pooled OR for inadequate IPC training was 1.46 (95% CI 1.14–1.87; I^2^ = 59%, high heterogeneity) for SARS-CoV-2 infection, including data from 6,257 HCWs derived from 7 studies^32,33,43,47,50,51,60^ (Fig. 5a). Funnel and Doi plots did not reveal substantial publication bias (LFK index = − 0.4; Supplementary Fig. 11). Subgrouping by VOCs was deemed inappropriate due to limited available data while alpha or delta strains were circulating for IPC training and other risk factors evaluated below. Aerosol-generating procedures are generally defined as medical interventions that produce aerosols, potentially increasing the risk of transmission of SARS-CoV-2 among HCWs. Commonly recognised aerosol-generating procedures include intubation and extubation, non-invasive ventilation, tracheotomy, bronchoscopy, sputum induction, open suctioning of airways, high-flow oxygen therapy, and nebulizer therapy^61^. There was some disagreement on the exact procedure list (e.g., the UK^61^ or CDC^62^ guidelines). Among 12 studies assessing infection risk of performing aerosol generating procedures, 8 studies did not specify guidelines^31,37,43,46,47,55,63,64^ and other four studies used the CDC^40,41,58^ or Swiss^65^ guidelines to define aerosol generating procedures, highlighting the need for standardised definitions in research studies. Performing aerosol generating procedures was associated with higher odds (OR 1.36; 95% CI 1.21–1.52; I^2^ = 24%, low heterogeneity) of SARS-CoV-2 infectivity, including data from 36,804 HCWs extracted from 12 studies^37,40,41,43,46,47,55,58,63,65^ (Fig. 5b). Despite the anticipated higher risk of infection among HCWs performing these procedures, funnel and Doi plots implied potential publication bias (LFK index = − 3; Supplementary Fig. 11). Using the trim-and-fill method (Table 2), the analysis included 15 studies, with 3 additional studies added, encompassing a total of 38,597 observations (9486 exposed and 29,111 control) and 4469 events. The random effects model yielded an OR of 1.43 (95% CI 1.24 to 1.66), indicating a statistically significant increased risk (z = 4.81, *p* < 0.0001). Heterogeneity was moderately quantified with I^2^ of 45.1% (95% CI 0.0%-70.0%).

### Environment services and hygiene

**Fig. 6:**
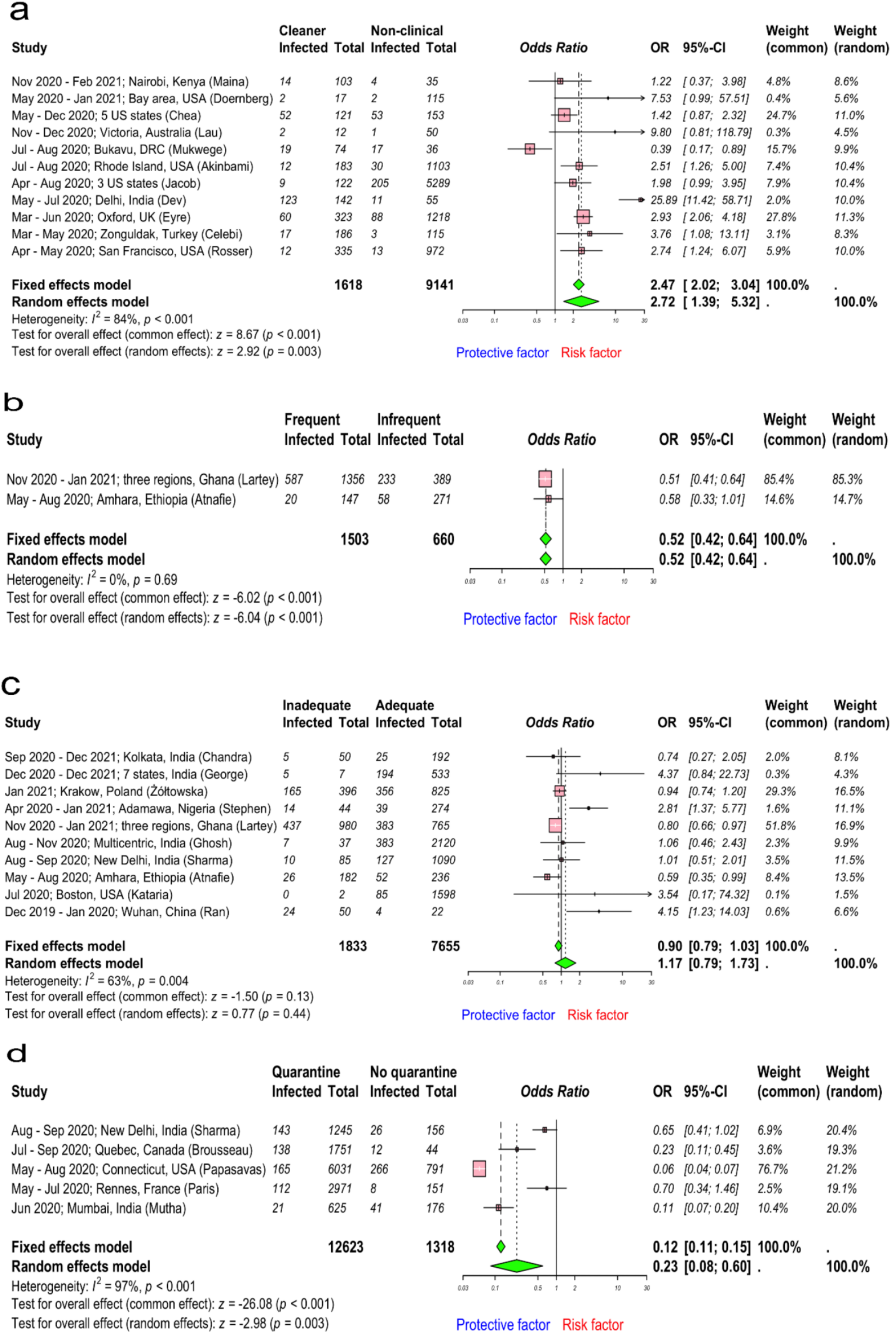
Forest plot to evaluate whether SARS-CoV-2 infection rates differed with job profile as cleaner, frequent decontamination of high touch areas, hand hygiene, and quarantine among healthcare workers. (**a**–**d**) describes risk assessment following job profile (as cleaner), frequent decontamination of high touch areas, hand hygiene, and history of quarantine. Data are presented as odds ratios, ORs; and 95% confidence interval, CI. Meta-analysis of effect estimates was performed using the metabin function (meta^22^ package) in R version 4. 4.1. Pooling of studies was performed using Mantel–Haenszel method. Diamonds representing meta-analysis results are printed in green colour and squares representing individual study results are printed in pink colour. I^2^ is a statistic used in meta-analysis to estimate the proportion of total variance in effect sizes due to heterogeneity between studies.

Intriguingly, the pooled OR for history of working as a cleaner was 2.72 (95% CI 1.39–5.32; I^2^ = 84%, high heterogeneity), using data from 10,759 HCWs retrieved from 11 studies^8,34,40,58,63,64,66–70^ (Fig. 6a). Funnel and Doi plots did not imply publication bias (LFK index = 0.6; Supplementary Fig. 12a,b). We investigated whether frequent decontamination of high touch surfaces was a protective factor against SARS-CoV-2 infection. The pooled OR effect estimates for frequent decontamination of high touch surfaces was 0.52 (95% CI 0.42–0.64; I^2^ = 0%, low heterogeneity) from 2163 HCWs (Fig. 6b). Potential publication bias was implied in both funnel and Doi plots (LFK index = 4; Supplementary Fig. 12c,d), although this analysis included only two studies^51,53^. In addition, hand hygiene is an important protective factor against infections. However, hand hygiene practices are often in reality not optimal. We included data on inadequate hand hygiene as a risk factor for SARS-CoV-2 infection from 10 studies^32,33,43,45,47,49,51,53,57,71^ involving a total of 9,488 HCWs. The evaluation of hand hygiene practices differed across studies, with some adhering to the national or global^72^ guidelines and others relying on single-question surveys such as consistency of hand washing. Nevertheless, we found that HCWs with inadequate hand hygiene practices had higher odds of being infected with SARS-CoV-2 (pooled OR 1.17; 95% CI 0.79–1.73; I^2^ = 63%, high heterogeneity) than those with good hand hygiene practices (Fig. 6c). These results are imprecise with wide CIs, and potential publication bias was implied in both funnel and Doi plots (LFK index = 6; Supplementary Fig. 12e,f); however, it could also be contributed by small study effects and/or between studies heterogeneity^38,39^. The trim-and-fill method included 13 studies, with 3 additional studies, comprising a total of 11,700 observations (1892 exposed and 9808 control) and 2653 events. The random effects model yielded an OR of 0.96 (95% CI 0.60–1.52) (Table 2), indicating no significant association (*p* = 0.8476), although heterogeneity was high (I^2^ = 66.2% (95% CI 39.2–81.2%)).

### Quarantine

We extracted data from 5 studies^54,55,71,73,74^ investigating the history of quarantine as a risk factor for SARS-CoV-2 infection. HCWs with a history of quarantine were removed from work because of a close contact with a COVID-19 case. However, one study^73^ quarantined HCWs who suffered from COVID-19 like illness, which might influence the results, and this situation is more accurately described as isolation than quarantine. It reflects a different risk profile and prior probability of SARS-CoV-2 infection compared to asymptomatic individuals who are removed from work based on contact tracing. Data from a total of 13,941 HCWs was included in this analysis. We found the pooled OR for history of quarantine was 0.23 (95% CI 0.08–0.60; I^2^ = 97%) against SARS-CoV-2 infection, suggesting that HCWs with a history of quarantine were protected against SARS-CoV-2 infection when returning to work (Fig. 6d). Although funnel and Doi plots indicated potential publication bias (LFK index = 5; Supplementary Fig. 12g,h), asymmetric plots could be due to heterogeneity or limited number of included studies^38,39^. The trim-and-fill method analysis included 8 studies, with 3 additional studies, comprising a total of 20,259 observations (18,590 exposed and 1669 control) and 1371 events. The random effects model yielded an OR of 0.07 (95% CI 0.02 to 0.25) (Table 2), indicating a significantly reduced risk (*p* < 0.0001). Heterogeneity was high, I^2^ of 97.9% (95% CI 97.0–98.5%). Data was not available with circulating alpha/delta variants or for how long the HCWs were in quarantine or how long they had been back at work before the data was collected.

### Household exposure to SARS-CoV-2

**Fig. 7:**
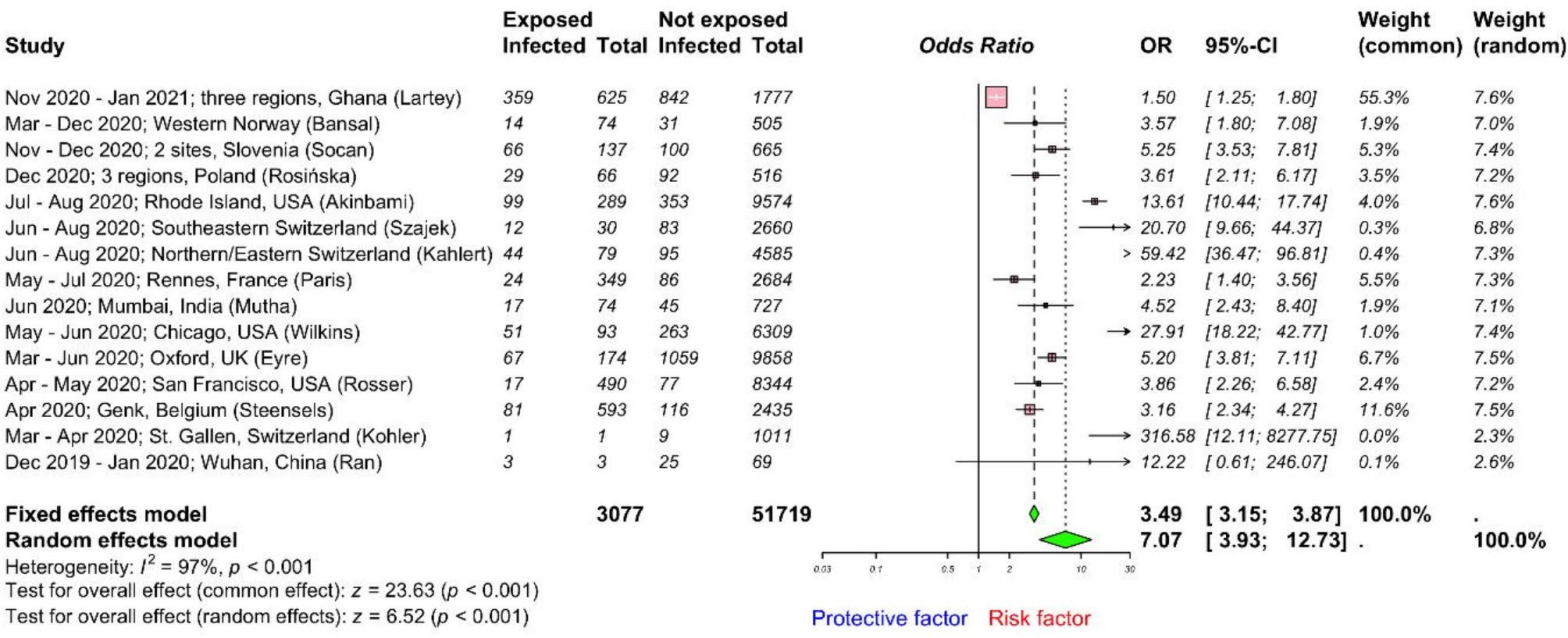
Forest plot to evaluate whether SARS-CoV-2 infection rates differed with household exposure to SARS-CoV-2 among healthcare workers. Data are presented as odds ratios, ORs; and 95% confidence interval, CI. Meta-analysis of effect estimates was performed using the metabin function (meta^22^ package) in R version 4. 4.1. Pooling of studies was performed using Mantel–Haenszel method. Diamonds representing meta-analysis results are printed in green colour and squares representing individual study results are printed in pink colour. I^2^ is a statistic used in meta-analysis to estimate the proportion of total variance in effect sizes due to heterogeneity between studies.

Available data was retrieved from 15 studies^37,40,49,52–55,65,69,70,75–77^ including 54,796 HCWs and the pooled OR calculated (Fig. 7). We found that the odds of being infected with SARS-CoV-2 were much higher (OR 7.07; 95% CI 3.93–12.73; I^2^ = 97%, high heterogeneity) for HCWs with household exposure to SARS-CoV-2 than without. Therefore, HCWs who were exposed to SARS-CoV-2 within their household had 7 times more odds to be infected with SARS-CoV-2 than without. This highlights the importance of strengthening IPC practices, both at home and at work. Additionally, potential publication bias was implied in both funnel and Doi plots (LFK index = 3; Supplementary Fig. 13). However, any visual imbalance in the funnel plot and false positive LFK index could also result from true heterogeneity rather than only publication bias^38,39^. The trim-and-fill method included 22 studies, with 7 additional studies, encompassing a total of 80,301 observations (3709 exposed and 76,592 control) and 5364 events. The random effects model indicated a significantly increased risk with an OR of 2.76 (95% CI 1.24–6.14), supported by a *p*-value of 0.0128. Heterogeneity was high, I^2^ of 98.0% (95% CI 97.6–98.4%).

### Biases and sensitivity analyses

Some of funnel and Doi plots implied potential publication bias (except meta-analyses assessing the role of inadequate IPC training, insufficient PPE use, and working as a cleaner). Nevertheless, these plots might also report false positive results due to between-study heterogeneity, number and size of studies, rather than publication bias alone^38,39^. Estimates from random-effects models may be more appropriate, as they account for potential heterogeneity between studies. Fixed-effects could be considered, where there is low heterogeneity (such as performed aerosol-generating procedures and frequent decontamination); however, overall results of both models in such cases were similar. Furthermore, we evaluated the potential for bias in each estimate arising from several sources using ROBINS-E and robvis tools^21^. These include confounding variables, measurement errors in exposure or risk factor classification, selection bias, interventions after exposure (e.g., vaccination), missing data, outcome measurement errors (e.g., PCR and serology tests), and reporting bias. Risk of bias assessment was conducted by two independent reviewers. We then assigned each study an overall risk-of-bias rating (low, some concerns, high, and very high) based on the domain and the estimate judged to have the highest risk of bias. We found that 24 studies had low concerns and 39 had some concerns (Supplementary Fig. 1). As no individual study was rated as having high concerns for bias, sensitivity analysis was not deemed necessary.

## Discussion

In these meta-analyses, we analysed SARS-CoV-2 infection rates and potential risk factors for infection in HCWs during the COVID-19 pandemic as a case study for future pandemic preparedness. We found high global SARS-CoV-2 infection rates of 11% among HCWs, which were influenced by circulating VOCs and multiple risk factors. The correlation between infection rates and specific SARS-CoV-2 strains highlights the virus’s evolving impact on prevention strategies. We identified the potential risk factors for SARS-CoV-2 infection in HCWs to reduce infection rates, including household exposure, occupational exposure, PPE usage, and quarantine. We found a generally high risk of being infected by household exposure to SARS-CoV-2 globally. This suggests that IPC measures and practices are often difficult to follow at home. Our meta-analyses revealed that working in the cleaning profession, and inadequate IPC practices substantially increased SARS-CoV-2 infection risk among HCWs. Conversely, quarantine and frequent cleaning and disinfection of high-touch surfaces were protective. Our findings provide valuable insights and improve the implementation of evidence-based preventive strategies during potential respiratory virus outbreaks.

Klompas and Rhee^78^ proposed a simplified approach to respiratory viral precautions, suggesting a single set of measures that would apply to all respiratory viruses, including gowns, gloves, eye protection, and fitted respirators in well-ventilated areas. This would replace the current system, which uses different precautions for different viruses and lacks a strong evidence base^78^. It is important to incorporate enhanced predicting methodologies for extreme scenarios and a robust evidence-based PPE inventory management strategy as critical elements for future pandemic preparedness plan. These lessons can help to protect HCWs and patients preventing the spread of respiratory viruses. Notably, we did not directly compare the epidemiology of SARS-CoV-2 infection among HCWs to that of other respiratory viral infections such as influenza, SARS, and Middle East respiratory syndrome (MERS). Furthermore, new variants may be more infectious and require adjustments in different approaches.

During 2020 and 2021, our pooled data showed that 11% of HCWs globally were infected with SARS-CoV-2. However, there was high variability among studies as infection rates ranged from ≤ 2% (in some high-income countries: Australia^34^, Germany^35^, Japan^36^ and Switzerland^37^) to more than 50% (Ghana^53^, Mexico^9^, low- and middle-income countries (LMICs)). Explicitly, the highest SARS-CoV-2 infection rate was found among HCWs in Mexico^9^ 59% during August 2020–January 2021, 43% in Poland^33^ in January 2021, and 41% in the DRC^8^ during July–August 2020. Mexico, Poland and DRC experienced multiple waves of SARS-CoV-2, with community prevalence reaching up to 25%^79–82^, which may have contributed to the higher infection rates in these countries. A lower number of studies included were from Oceania (n = 2), South America (n = 1), and Africa (n = 5) compared to Europe (n = 24) and North America (n = 17). Only two of the five African studies assessed occupational exposure, and four assessed PPE use in relation to SARS-CoV-2 infection among HCWs. This dearth of research investigating risk factors associated with infection among African HCWs hinders the formulation of definitive conclusions. Furthermore, HCWs in LMICs face unique challenges compared to their counterparts in high-income countries. These challenges include relative HCW shortages^83^, exposure to misinformation and misogyny, and the emotional toll of family separation during outbreaks^84^.

Differences in healthcare systems across various countries and regions play a major role, as they influence the protocols and resources available for managing infections including availability of PPE and guidelines for its usage. The prevalence of community transmission also contributes to differing infection rates among HCWs. Regions with higher community transmission rates are likely to see more cases within healthcare settings, as the virus is more prevalent in the general population. Notably, we observed higher SARS-CoV-2 infection rates among HCWs in Africa relative to those in Europe and America. Economic burden from HCW infection is greatest in countries with low HCW density and severe staff shortages^85^. Secondary infections and excess maternal and child mortality constitute the heaviest costs. For instance, the economic burden of secondary infections surpassed that of direct HCW infections, constituting between 13 and 70% of the total costs attributable to HCW infections in Kenya and South Africa^85^. SARS-CoV-2 infection claims in Victoria, Australia, were predominantly (53%) from HCWs, peaking in July–October 2020. This was a significantly higher claim rate than other industries^86^ (17 vs. 2 per 10,000 employed), highlighting the need for strengthening IPC measures in both high and low resource settings.

Additionally, like other RNA viruses, SARS-CoV-2 mutates over time. These mutations in the virus, however, might alter its properties such as how quickly it spreads, the severity of the disease, and how well vaccination, medication, diagnosis, and other public health and social initiatives work. Potential transmission mechanisms^87^ of SARS-CoV-2 variants of concern are enhanced contagious window and viral shedding^88–92^, enhanced environmental stability^93,94^, and improved binding to host ACE2 receptors, and reduced cross-reactivity or decay of neutralising antibodies^95^. Published data collected from early 2020 to late 2021 were included in our meta-analyses. We observed substantial changes with differing circulating variants in which higher infection rates among HCWs were found when alpha (26%, 95% CI 17–36%) or delta (22%, 95% CI 7–52) VOCs circulated compared to when only ancestral viruses circulated (9%, 95% CI 8–12%). This is likely due to higher community spread despite likely improved access to PPE and increased knowledge gained from IPC training during the later stages of the pandemic. The alpha, delta and omicron variants had a greater community spread in 2020–2021^7,96^, which may influence infection rates. The emerging Omicron variants were linked to increased reinfections and breakthrough infections, which could lead to higher infection rates among HCWs despite vaccination^97,98^. Recently published studies have found 2–39% infection rates among HCWs when Omicron VOC circulated^99–103^. However, whether the infection rates in HCWs when Omicron VOC circulated are higher than when earlier VOCs were in circulation remains unanswered. We need more data on newer highly transmissible and immune evading variants such as Omicron, especially data from LMICs together with vaccination status, to discuss their potential implications on infection rates among HCWs.

HCWs have been disproportionately affected by COVID-19 due to direct exposure to patients, experiencing high rates of infection and mortality^104^. We found a generally moderate occupational risk of SARS-CoV-2 infection among HCWs globally (OR 1.8; 95% CI 1.5–2.1). Three studies^59,105,106^ found high infection rates (14–27%) among HCWs in the New York area (USA) in 2020. However, only one study^105^ reported substantially higher risk of SARS-CoV-2 infection among HCWs exposed to individuals with COVID-19 (OR 26; 95% CI 24–26), which is potentially related to inadequate availability of PPE at work and higher community spread in the early period of COVID-19 pandemic. The availability and usage of PPE vary widely, impacting the level of protection for HCWs. In settings where PPE is insufficient or incorrectly used, the risk of infection is higher. We found some evidence to recommend mask wearing in healthcare settings based upon risk assessment (ORs with insufficient PPE use in HCWs were 1.45; 95% CI 1.14–1.84; I^2^ = 79%). Additionally, adequate caution should be taken to reduce risk of potential exposure to suspected/confirmed SARS-CoV-2 infection or other respiratory infection (e.g., high-risk procedural exposure, and close contact with patients and household members). Sufficient PPE use, based upon occupational exposure and risk-assessment, within healthcare settings is essential in preventing viral spread. HCW SARS-CoV-2 infection clusters were observed in general wards with surgical mask recommendations, while no workplace-acquired infections were reported in critical care wards with respirator recommendations^16^. A systematic review published in 2020 found that HCWs who wore particulate-filtering facepiece respirator (N95 or equivalent) had a significantly lower risk of SARS-CoV-2 infection as compared to those who wore surgical masks or no mask^107^. In our Norwegian cohort study, the SARS-CoV-2 infection rate was 10% in 2020. This is three times higher than the estimated community rate of 3%^28,108–110^. This disparity is notable, given the availability of stringent IPC measures and paid sick leave. It underscores the urgent need to further strengthen IPC measures, even in high-income countries. Particular attention should be paid to household transmission and identifying vulnerable groups that may be overlooked. Additionally, HCWs using partial PPE when managing patients with SARS-CoV-2 had 2.5-fold higher odds of being seropositive than HCW with no COVID-19 patient contact in 2020^28^. As universal masking was associated with lowered SARS-CoV-2 incidence among HCWs, while the infection rate in the nearby population continued to rise, it protected HCWs against respiratory illness^111^. During a respiratory viral outbreak, exposure-based PPE use by HCWs is reasonable, particularly throughout patient contacts and in at-risk departments (e.g., emergency department, intensive care unit, and while managing patients at extremes of age and those with immunosuppression).

IPC policies evolved during the pandemic, along with availability of vaccines, which might have reduced transmission. A scoping review revealed that only a few IPC guidelines from LMICs addressed the need for workplace induction training, and the application of adult learning principles. Finally, the mode of delivery and curriculum varied across guidelines with limited evaluation frameworks for training programs^112^. The transition from pre-vaccination to post-vaccination phases and its impact on the identified risk factors in HCWs is also important, although specific data was not available in this review. During the initial phase of the pandemic, before vaccines were available, HCWs faced high infection rates due to direct patient contacts, insufficient PPE, and the lack of effective treatments with key potential risk factors including prolonged exposure, PPE shortages and high patient load. With the rollout of vaccination campaigns, the dynamics of infection rates and potential risk factors shifted to include vaccination status, breakthrough infections and vaccine hesitancy. The undiagnosed SARS-CoV-2 infection rates were higher with newer variants (e.g., Omicron) with reduced relative vaccine effectiveness with a two-dose vaccine schedule^99,101,113^. Fortunately, high antibody levels in HCWs after third dose of mRNA vaccine are associated with reduced Omicron infection and disease risk^114^. A higher willingness to take a COVID-19 vaccine has been observed in LMICs (mean: 80%), compared to the United States (mean 65%) and Russia (mean 30%)^115^. It is important to update vaccine formulations, support vaccination campaigns addressing safety concerns, and continuously adapt IPC measures to reflect the evolving infection risk landscape, ensuring both vaccinated and unvaccinated HCWs are adequately protected.

The effectiveness of quarantine and frequent decontamination can vary significantly based on local practices, resources, and the stage of the pandemic. A history of quarantine was found to be a protective factor against SARS-CoV-2 infection in HCWs (OR 0.23; 95% CI 0.08–0.60). Physical distancing and ‘stay at home’ mandates played an important role in the pandemic to limit the spread of the virus^13,107,116^. Australian researchers estimated that community physical distancing behaviour lowered the risk of SARS-CoV-2 transmission/infection by 7 to 56% in Victoria in 2020^15^. Although, these are likely dependent on community resilience and socio-cultural factors. Frequent decontamination of high-touch areas emerged as a protective factor against SARS-CoV-2 infection in our analysis. Although our data did not conclusively identify inadequate hand hygiene as a risk factor due to wide CIs, the general recommendation for diligent hand hygiene in healthcare settings remains paramount^72^. SARS-CoV-2 is likely transmitted through a spectrum rather than a single predominant mode^117,118^. Transmission may primarily occur through-the-air with infectious respiratory particles (akin to droplets transmission)^119^ and fomites^120^ in close contact situations; however, it is less likely through faecal–oral^121^ routes. Atsushi^122^ et al. developed a model to assess the risk of SARS-CoV-2 infection from multiple exposure pathways in healthcare settings such as hand contact, droplet spray, and inhalation of inspirable and respirable particles. They found that droplet spray was the major infection pathway (contributing to 60–86% cases), followed by hand contact (9–32%). Regular cleaning of high-touch surfaces and targeted quarantine for high-risk individuals or those with confirmed exposure seems practical and might be resource-efficient, although with varying feasibility and effectiveness in different healthcare contexts. In settings where strict quarantine or frequent decontamination might be challenging, sufficient PPE use, and adequate IPC training could help to reduce SARS-CoV-2 transmission among HCWs. In high-resource settings, strict quarantine protocols and frequent decontamination are more easily implemented and maintained. However, in LMICs, these measures might be challenging due to limited access to necessary supplies and infrastructure. Similarly, facilities with robust IPC programs are better equipped to enforce quarantine and decontamination protocols effectively. During the early stages of the pandemic, strict quarantine and frequent decontamination were important in controlling the spread of the virus. However, as the pandemic progressed and vaccination campaigns were rolled out, the reliance on lockdowns, physical distancing, quarantine and decontamination decreased. LMICs can consider low-cost strategies to mitigate infections, such as home quarantine and affordable effective cleaning solutions.

Our findings highlight the importance of taking preventive measures to reduce the risk of SARS-CoV-2 infection, such as training, wearing appropriate PPE, quarantine, washing hands frequently, and decontamination of high touch areas, in the event of respiratory viral outbreaks. The ORs for inadequate IPC training and insufficient PPE use were similar, and it suggests that both factors are similarly associated with the infection. Both inadequate IPC training and insufficient PPE use could independently increase the odds of the infection among HCWs due to the gap between theoretical knowledge and practical application. Furthermore, we found a paradox that working as a cleaner was risk factor while frequent decontamination of high-touch surfaces was a protective measure against SARS-CoV-2 infection. Considering the vulnerability of cleaners, we highlight the need for rigorous training programs, along with feasibility studies investigating innovative approaches such cost-effectiveness of adjunct automated decontamination systems. These measures should be implemented as part of a comprehensive strategy to control the viral spread. While IPC training is noticeably important, other measures such as HCW scheduling, access limitations, and work process design, may also be helpful and require further studies to determine their role in mitigating viral spread.

We found a generally high risk of being infected after household exposure to SARS-CoV-2 globally (OR 7; 95% CI 4–13). Particularly, several studies found higher household risk among HCWs in Switzerland^37,52,65^ with ORs ranging 20–317; and Chicago, USA^75^ (OR 28; 95% CI 18–43) which is likely related to inadequate IPC precautions at home. Household exposure is a risk factor for the whole household. HCWs have a likely higher baseline exposure risk to SARS-CoV-2 due to their work profile^104^. When two risk factors come together, HCWs face a substantially higher risk of infection compared to the public with household exposure alone. This is detrimental as frontline HCWs are regularly in contact with vulnerable patients who are at-risk of severe COVID-19. Therefore, exposure limitation strategies should be followed both at work and at home. Individual institutes may need further refined strategies that can be applied effectively at home, which will also benefit the general population.

### Limitations of the review

This review has a few limitations. The quality of included studies was inconsistent with high heterogeneity in results and asymmetric funnel plots in some of the meta-analyses. Larger studies, often characterised by more rigorous methodologies, exhibited greater effect sizes. It is plausible that some smaller studies employed different methods, contributing to their stronger results. Publication bias can significantly inflate effect estimates and skew conclusions, leading to an overestimation of infection risks or protective effects, with significant results are more likely to be published. We acknowledge that observed high infection rates among HCWs might be partly due to publication bias; therefore, we conducted additional trim-and-fill method in cases with potential of publication bias. Similarly, the protective effects of some factors (e.g., PPE) might be overestimated if studies showing minimal or no effect are unpublished or underrepresented. The heterogeneity observed between countries could be attributed to diverse factors like socioeconomic status, lifestyle choices, cultural practices, and hospital infection control policies. It is worth considering that high degrees of heterogeneity may reflect substantial variations in pre-existing immunity among HCWs across various geographical regions, age groups, genders, and community transmission patterns. Unfortunately, detailed covariate information (e.g., age and sex) was not available for most of the studies to perform stratified and sensitivity analyses. These factors, combined with variations in study methodologies, contribute to the observed differences in infection rates and associated risk factors across the included studies. Drawing clear conclusions is hampered by some concerns of the risk of bias in included studies and differing national recommendations and spread of the virus. As an alternative approach, we utilised a random-effects model, assuming that the effects being evaluated across studies do not follow identical patterns but rather conform to a distribution with an average effect at the centre and a dispersion representing the heterogeneity’s extent. A notable geographic bias was observed in the reviewed studies, with a preponderance of research originating from high-income countries. The limited data available from LMICs underscores the urgent need for further investigation in these regions to gain a more comprehensive understanding of the global impact of differing occupational practices and household exposure on SARS-CoV-2 infection, enabling the identification of subpopulations or departments at risk of infection. The majority of the studies included in this review were cross-sectional. Due to smaller subgroup sizes, we were unable to conduct subgroup analyses by different study designs and VOCs for all included risk factors.

### Future research

Emerging variants may increase transmission, severity, and vaccine resistance, potentially leading to higher infection rates, increased hospitalisations, and disruptions in healthcare services. Despite a comprehensive search strategy, no relevant data on later variants (e.g., Omicron) were identified in HCWs. Omicron, known for its higher transmissibility and partial immune evasion, has dramatically altered the landscape of infection rates and transmission dynamics^97,98^. This highlights the need for continuous monitoring and updating of risk assessments to include the latest variant data. It is important that all relevant data, regardless of the significance of the findings, are considered for publication. This comprehensive approach will help in developing more effective and evidence-based strategies for protecting HCWs and managing infection risks in healthcare settings. To maximize the effectiveness of interventions, future reviews could conduct a more detailed analyses of risk factors and their interactions with specific viral variants, particularly post-Omicron which was not covered in this review.

The effectiveness of interventions observed in high-income countries might not translate directly to settings with resource constraints. In resource-constrained settings, where community transmission rates are high and access to PPE and other protective measures is limited, HCWs can be at increased risk of infection. To provide a more comprehensive understanding of global HCW risk, future research should focus on underrepresented regions such as LMICs. These regions may have higher community transmission rates, limited access to PPE, lower numbers of HCWs, and different working conditions, which could significantly affect infection rates among HCWs. In addition, the role of differing testing strategies, environmental cleaning, and ventilation are potentially important for healthcare capacity in an unexplained respiratory outbreak; however, we could not find data quantifying this risk in HCWs. More research is needed to optimise their cost-effective use in different healthcare settings.

The ROBINS-E tool was designed to evaluate the risk of bias in non-randomised studies of exposures, including confounding, measurement of the exposure, selection of participants, post-exposure interventions, missing data, measurement of outcome and selection of the reported result. We included unadjusted estimates and therefore, the reported effect of risk factors might be over- or underestimated. Additionally, HCWs work in diverse setting ranging from outpatient to intensive care units, potentially skewing infection rate estimates. There is also a need for standardised definition of exposure and interventions because this can lead to misclassification bias, affecting the reliability of the identified risk factors. To mitigate the impact of study quality on the results, future research should standardise definitions of exposures and measurements, random sampling to enhance the generalisability of the findings and encourage publication of all results.

### Practical recommendations

The IPC policies should focus on both the household exposure and known occupational practices such as improper IPC training, insufficient PPE use, and aerosol-generating procedures. Additionally, individuals at-risk of infection should be promptly identified during outbreaks, especially those who are often underrepresented and vulnerable. For instance, we found higher odds of infection among hospital cleaning staff, which might be related to their exposure to infectious virus or regular mobility between different departments or other socio-economic factors. The applicability of our findings could be expanded by conducting more research on protective effects of targeted training modules (e.g., occupation-based training, simulation, and regular refresher courses on the latest IPC measures), strategies for effective PPE distribution (e.g., centralised PPE management, PPE fit testing and training, emergency PPE reserves), tailored IPC policies for high-risk situations (e.g., dedicated high-risk zones, dual or enhanced protective measures, regular health monitoring of at-risk individuals), cost-effectiveness of different ventilation or environmental cleaning methods, paid sick leaves and telemedicine.

## Conclusion

In the case of an epidemic or pandemic, infection control procedures must be strictly enforced as top priority to protect HCWs. Evidence-based measures for reducing respiratory transmission in healthcare settings include ensuring quarantine or sick leave to HCWs with household or occupational exposure, adequate IPC training and PPE use for all HCWs including cleaners, and frequent decontamination of high touch areas. Given some potential for biases in individual studies, future meta-analyses that incorporate data from newer VOCs and multi-risk factor analyses will be essential for updating our understanding. A quick and organised response is needed to prevent the spread of respiratory viruses. To effectively mitigate the transmission of SARS-CoV-2 among HCWs, our findings should be adapted to regional contexts, considering variations in SARS-CoV-2 community spread and risk assessments across countries.

## Electronic supplementary material

Below is the link to the electronic supplementary material.

Supplementary Material 1

## Data Availability

No new original data is generated. All the data extracted from the published papers is included with the submission. This paper does not report original code. This project leverages open-source R code. We have documented the specific functions used in relevant sections. Any additional information required to reanalyse the data reported in this paper is available from the corresponding authors upon reasonable request.
