# Supplementary material for: SARS-CoV-2 infection rates and associated risk factors in healthcare workers: systematic review and meta-analysis
Source: Sci Rep. 2025 Feb 8;15:4705. doi: 10.1038/s41598-025-89472-5 (PMC11807171; doi:10.1038/s41598-025-89472-5)
Supplement: Supplementary file 1 — Supplementary Material 1 [file 41598_2025_89472_MOESM1_ESM.pdf]

Supplementary materials

Title: SARS-CoV-2 infection rates and associated risk factors in healthcare workers: systematic review and meta-analysis

Amit Bansal[1,2,3*], Mai-Chi Trieu[1,4], Emily M Eriksson[5], Fan Zhou[1], Jodie McVernon[2], Karl Albert Brokstad[1,6], Rebecca Jane Cox[1,7, *]

[1]Influenza Centre, Department of Clinical Science, University of Bergen, Bergen, Norway
[2]Department of Infectious Diseases, University of Melbourne, at the Peter Doherty Institute for Infection and Immunity, Victoria, Australia
[3]Norwegian School of Sport Sciences, Oslo, Norway
[4]Department of Microbiology and Immunology, Peter Doherty Institute for Infection and Immunity, University of Melbourne, Melbourne, Victoria, Australia
[5]Population Health and Immunity Division, the Walter and Eliza Hall Institute of Medical Research, Melbourne, Australia
[6]Department of Safety, Chemistry and Biomedical Laboratory Sciences, Western Norway University of Applied Sciences, Bergen, Norway
[7]Department of Microbiology, Haukeland University Hospital, Bergen, Norway

*Corresponding authors

Table of Contents

Supplementary figure 1: Risk of bias assessment 3

Supplementary figure 2: SARS-CoV-2 infection rates subgrouped by study region or continent 4

Supplementary figure 3: SARS-CoV-2 infection rate subgrouped by study design 5

Supplementary figure 4: Funnel and Doi plots for meta-analysis involving SARS-CoV-2 infection rate estimation 6

Supplementary figure 5: Occupational exposure to SARS-CoV-2 by study region or continent 7

Supplementary figure 6: Occupational exposure to SARS-CoV-2 by study design 8

Supplementary figure 7: Funnel and Doi plot for meta-analysis involving occupational exposure to SARS-CoV-2 9

Supplementary figure 8: Occupational exposure to SARS-CoV-2 with insufficient personal protective equipment (PPE) use by study region or continent 10

Supplementary figure 9: Occupational exposure to SARS-CoV-2 with insufficient PPE use by study design 11

Supplementary figure 10: Funnel and Doi plot for meta-analysis involving PPE use 12

Supplementary figure 11: Funnel and Doi plot for meta-analyses involving infective prevention and control (IPC) training and aerosol-generating procedures 13

Supplementary figure 12: Funnel and Doi plot for meta-analyses involving working as a cleaner, decontamination, hand hygiene and quarantine 14

Supplementary figure 13: Funnel and Doi plots for meta-analysis involving household exposure to SARS-CoV-2 15

Supplementary Table 1: Study inclusion and exclusion criteria 16

Supplementary Table 2: Search strategy for Ovid MEDLINE(R) 17

Supplementary Table 3: Search strategy for Embase 21

Supplementary Table 4: Search strategy for Google Scholar 24

Supplementary Table 5: Characterising main findings of the included 63 articles. 25

Supplementary figure 1: Risk of bias assessment

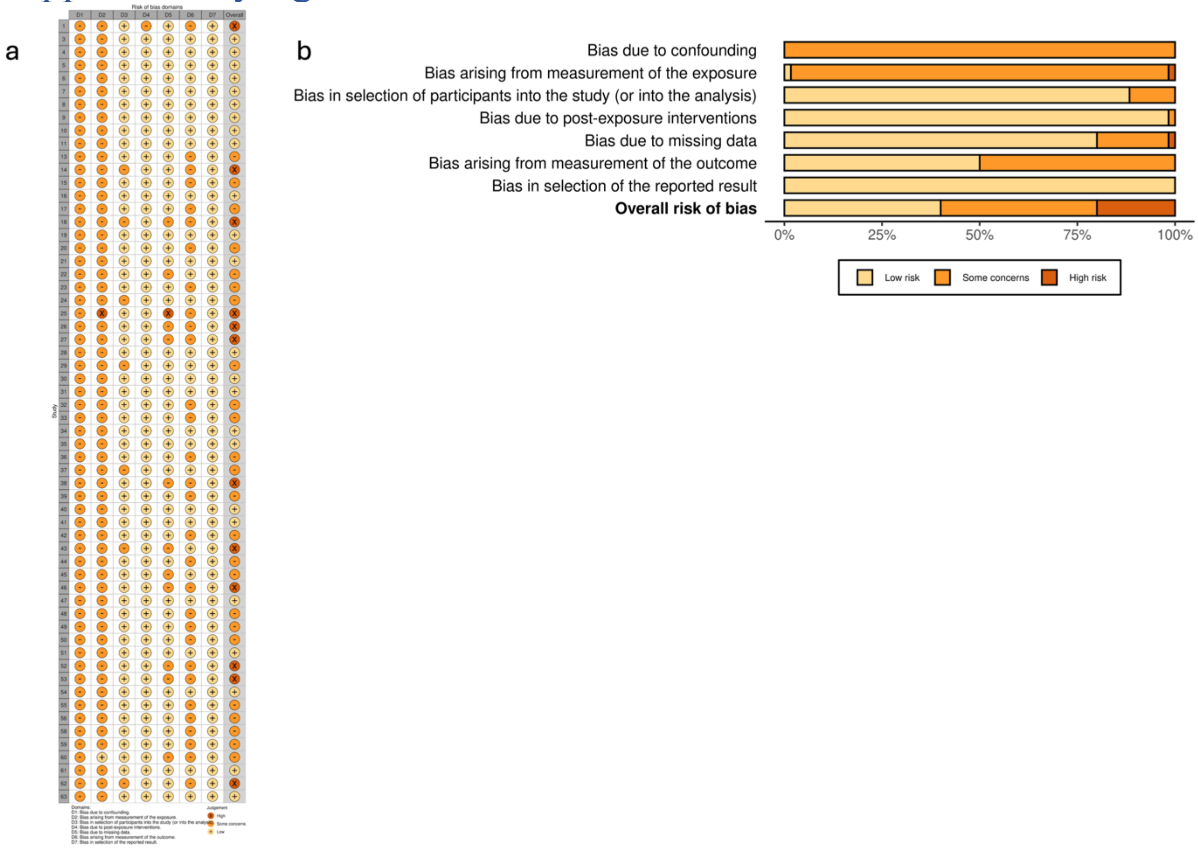

Supplementary figure 1a and 1b visualise traffic-light and summary plots of included studies, using the ROBINS-E (Risk Of Bias In Non-randomized Studies - of Exposures) tool.

Supplementary figure 2: SARS-CoV-2 infection rates subgrouped by study region or continent

Study	Events	Total	Proportion	95%-CI	Weight (common)	Weight (random)
**subgroup = Africa**						
Nov 2020 - Feb 2021; Nairobi, Kenya (Maina)	300	1631	0.18	[0.17; 0.20]	1.0%	1.6%
Apr 2020 - Jan 2021; Adamawa, Nigeria (Stephen)	53	318	0.17	[0.13; 0.21]	0.2%	1.6%
Nov 2020 - Jan 2021; three regions, Ghana (Lartey)	1201	2402	0.50	[0.48; 0.52]	2.3%	1.6%
Jul - Aug 2020; Bukavu, DRC (Mukwege)	148	359	0.41	[0.36; 0.47]	0.3%	1.6%
May - Aug 2020; Amhara, Ethiopia (Atnafie)	78	418	0.19	[0.15; 0.23]	0.2%	1.6%
**Fixed effects model**		**5128**	**0.37**	**[0.35; 0.38]**	**4.1%**	**.**
**Random effects model**			**0.27**	**[0.15; 0.45]**	**.**	**8.1%**
Heterogeneity: I^2 = 99%, p < 0.001						
Test for effect in subgroup (common effect): z = NA (p = NA)						
Test for effect in subgroup (random effects): z = NA (p = NA)						
**subgroup = Asia**						
Sep 2020 - Dec 2021; Kolkata, India (Chandra)	30	242	0.12	[0.09; 0.17]	0.1%	1.6%
Dec 2020 - Dec 2021; 7 states, India (George)	345	973	0.35	[0.32; 0.39]	0.9%	1.6%
Aug - Nov 2020; Multicentric, India (Ghosh)	646	3253	0.20	[0.18; 0.21]	2.0%	1.6%
Aug - Sep 2020; New Delhi, India (Sharma)	169	1401	0.12	[0.10; 0.14]	0.6%	1.6%
Jul - Aug 2020; Kolkata, India (Goenka)	134	1122	0.12	[0.10; 0.14]	0.5%	1.6%
Jun - Jul 2020; New Delhi, India (Gupta)	487	3739	0.13	[0.12; 0.14]	1.7%	1.6%
May - Jul 2020; Delhi, India (Dev)	506	3100	0.16	[0.15; 0.18]	1.7%	1.6%
Jun 2020; Mumbai, India (Mutha)	62	801	0.08	[0.06; 0.10]	0.2%	1.6%
Jun 2020; Osaka, Japan (Nishida)	4	925	0.00	[0.00; 0.01]	0.0%	1.2%
Apr - Jun 2020; Tehran, Iran (Darvishian)	340	2065	0.16	[0.15; 0.18]	1.1%	1.6%
Mar - May 2020; Zonguldak, Turkey (Celebi)	50	703	0.07	[0.05; 0.09]	0.2%	1.6%
Dec 2019 - Jan 2020; Wuhan, China (Ran)	28	72	0.39	[0.28; 0.51]	0.1%	1.5%
Dec 2019 - Jan 2020; Wuhan, China (Wang)	12	118	0.10	[0.05; 0.17]	0.0%	1.5%
Apr - May 2020; multi-site, India (Chatterjee)	1073	21402	0.05	[0.05; 0.05]	4.0%	1.6%
**Fixed effects model**		**39916**	**0.12**	**[0.11; 0.12]**	**12.9%**	**.**
**Random effects model**			**0.12**	**[0.08; 0.17]**	**.**	**22.0%**
Heterogeneity: I^2 = 99%, p < 0.001						
Test for effect in subgroup (common effect): z = NA (p = NA)						
Test for effect in subgroup (random effects): z = NA (p = NA)						
**subgroup = Europe**						
Apr 2021; Dublin & Galway, Ireland (Allen)	898	5085	0.18	[0.17; 0.19]	2.9%	1.6%
Dec 2020 - Mar 2021; nationwide, UK (Martin)	2496	10772	0.23	[0.22; 0.24]	7.5%	1.6%
Jan 2021; Krakow, Poland (Żółtowska)	521	1221	0.43	[0.40; 0.46]	1.2%	1.6%
Mar - Dec 2020; Western Norway (Bansal)	127	1214	0.10	[0.09; 0.12]	0.4%	1.6%
Nov - Dec 2020; 2 sites, Slovenia (Socan)	166	813	0.20	[0.18; 0.23]	0.5%	1.6%
Dec 2020; 3 regions, Poland (Rosińska)	262	1040	0.25	[0.23; 0.28]	0.8%	1.6%
Oct 2020; Dublin & Galway, Ireland (Allen)	576	5787	0.10	[0.09; 0.11]	2.0%	1.6%
Jun - Aug 2020; Southeastern Switzerland (Szajek)	475	2794	0.17	[0.16; 0.18]	1.5%	1.6%
Jun - Aug 2020; Northern/Eastern Switzerland (Kahlert)	139	4664	0.03	[0.03; 0.04]	0.5%	1.6%
May - Jul 2020; Rennes, France (Paris)	122	3234	0.04	[0.03; 0.04]	0.5%	1.6%
May - Jun 2020; Brussels, Belgium (Blairon)	217	1485	0.15	[0.13; 0.17]	0.7%	1.6%
Mar - Jun 2020; Bergen, Norway (Trieu)	32	607	0.05	[0.04; 0.07]	0.1%	1.6%
Mar - Jun 2020; Oxford, UK (Eyre)	1128	10034	0.11	[0.11; 0.12]	3.9%	1.6%
Apr - May 2020; Danderyd, Sweden (Rudberg)	410	2149	0.19	[0.17; 0.21]	1.3%	1.6%
Apr 2020; Copenhagen, Denmark (Iversen)	1163	28792	0.04	[0.04; 0.04]	4.4%	1.6%
Apr 2020; Ticino, Switzerland (Piccoli)	454	4726	0.10	[0.09; 0.10]	1.6%	1.6%
Apr 2020; Genk, Belgium (Steensels)	197	3056	0.06	[0.06; 0.07]	0.7%	1.6%
Apr 2020; Madrid, Spain (Galan)	818	2590	0.32	[0.30; 0.33]	2.2%	1.6%
Apr 2020; Milan, Italy (Sotgiu)	29	202	0.14	[0.10; 0.20]	0.1%	1.6%
Mar - Apr 2020; Barcelona, Spain (Garcia-Basteiro)	54	578	0.09	[0.07; 0.12]	0.2%	1.6%
Mar - Apr 2020; St. Gallen, Switzerland (Kohler)	10	1012	0.01	[0.00; 0.02]	0.0%	1.4%
Mar - Apr 2020; Alicante, Spain (Algado-Selles)	784	3900	0.20	[0.19; 0.21]	2.4%	1.6%
Feb - Mar 2020; Naples, Italy (Fusco)	4	115	0.03	[0.01; 0.09]	0.0%	1.2%
Mar - Jul 2020; Hamburg, Germany (Brehm)	22	1253	0.02	[0.01; 0.03]	0.1%	1.5%
**Fixed effects model**		**97123**	**0.14**	**[0.14; 0.15]**	**35.5%**	**.**
**Random effects model**			**0.10**	**[0.08; 0.14]**	**.**	**38.2%**
Heterogeneity: I^2 = 100%, p < 0.001						
Test for effect in subgroup (common effect): z = NA (p = NA)						
Test for effect in subgroup (random effects): z = NA (p = NA)						
**subgroup = North America**						
Aug 2020 - May 2021; Atlanta, Georgia, USA (Dube)	72	203	0.35	[0.29; 0.42]	0.2%	1.6%
Aug 2020 - Jan 2021; Mexico city, Mexico (Bueno-Hernández)	64	109	0.59	[0.49; 0.68]	0.1%	1.6%
May 2020 - Jan 2021; Bay area, USA (Doernberg)	91	2435	0.04	[0.03; 0.05]	0.3%	1.6%
May - Dec 2020; 5 US states (Chea)	345	967	0.36	[0.33; 0.39]	0.9%	1.6%
Jul - Sep 2020; Quebec, Canada (Brousseau)	241	2056	0.12	[0.10; 0.13]	0.8%	1.6%
Jul - Aug 2020; Rhode Island, USA (Akinbami)	452	9864	0.05	[0.04; 0.05]	1.7%	1.6%
May - Aug 2020; Connecticut, USA (Papasavas)	433	6774	0.06	[0.06; 0.07]	1.6%	1.6%
Apr - Aug 2020; 3 US states (Jacob)	1080	24749	0.04	[0.04; 0.05]	4.0%	1.6%
May - Jun 2020; Atlanta, USA (Howard-Anderson)	23	353	0.07	[0.04; 0.10]	0.1%	1.5%
May - Jun 2020; California, USA (Brant-Zawadzki)	31	2924	0.01	[0.01; 0.02]	0.1%	1.6%
May - Jun 2020; Chicago, USA (Wilkins)	316	6510	0.05	[0.04; 0.05]	1.2%	1.6%
Apr - Jun 2020, New York, USA (Bryan)	4610	19107	0.24	[0.24; 0.25]	13.6%	1.6%
Apr - Jun 2020; Atlanta, USA (Baker)	582	10275	0.06	[0.05; 0.06]	2.1%	1.6%
Apr - Jun 2020; NYC, USA (Moscola)	5523	40329	0.14	[0.13; 0.14]	18.6%	1.6%
Mar - May 2020; NYC, USA (Venugopal)	137	500	0.27	[0.24; 0.32]	0.4%	1.6%
Apr - May 2020; San Francisco, USA (Rosser)	136	10449	0.01	[0.01; 0.02]	0.5%	1.6%
Jul 2020; Boston, USA (Kataria)	95	1743	0.05	[0.04; 0.07]	0.4%	1.6%
**Fixed effects model**		**139347**	**0.13**	**[0.13; 0.13]**	**46.6%**	**.**
**Random effects model**			**0.09**	**[0.06; 0.13]**	**.**	**27.4%**
Heterogeneity: I^2 = 100%, p < 0.001						
Test for effect in subgroup (common effect): z = NA (p = NA)						
Test for effect in subgroup (random effects): z = NA (p = NA)						
**subgroup = Oceania**						
Jun - Aug 2020; Western Australia (Ludewick)	4	799	0.01	[0.00; 0.01]	0.0%	1.2%
Nov - Dec 2020; Victoria, Australia (Lau)	13	706	0.02	[0.01; 0.03]	0.0%	1.5%
**Fixed effects model**		**1505**	**0.01**	**[0.01; 0.02]**	**0.1%**	**.**
**Random effects model**			**0.01**	**[0.00; 0.04]**	**.**	**2.7%**
Heterogeneity: I^2 = 81%, p = 0.02						
Test for effect in subgroup (common effect): z = NA (p = NA)						
Test for effect in subgroup (random effects): z = NA (p = NA)						
**subgroup = South America**						
Jun - Jul 2020; Cali, Colombia (Rodriguez-Lopez)	316	913	0.35	[0.32; 0.38]	0.8%	1.6%
**Fixed effects model**		**283932**	**0.14**	**[0.14; 0.14]**	**100.0%**	**.**
**Random effects model**			**0.11**	**[0.09; 0.13]**	**.**	**100.0%**

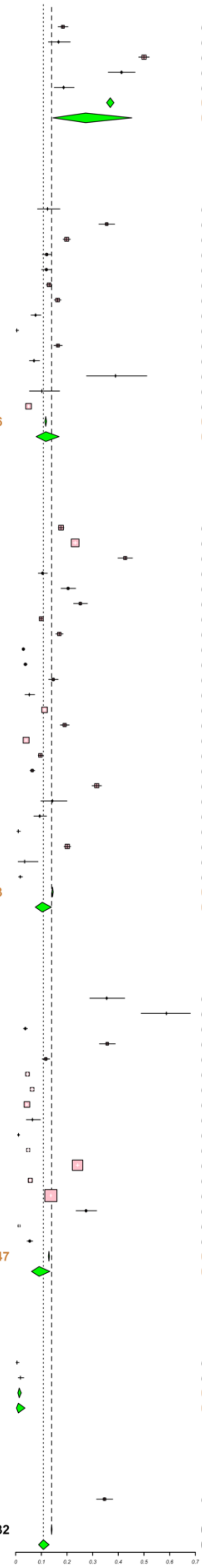

Heterogeneity: I^2 = 100%, p < 0.001
Test for subgroup differences (common effect): χ^2_5 = 2333.94, df = 5 (p < 0.001)
Test for subgroup differences (random effects): χ^2_5 = 159.06, df = 5 (p < 0.001)

Supplementary figure 3: SARS-CoV-2 infection rate subgrouped by study design

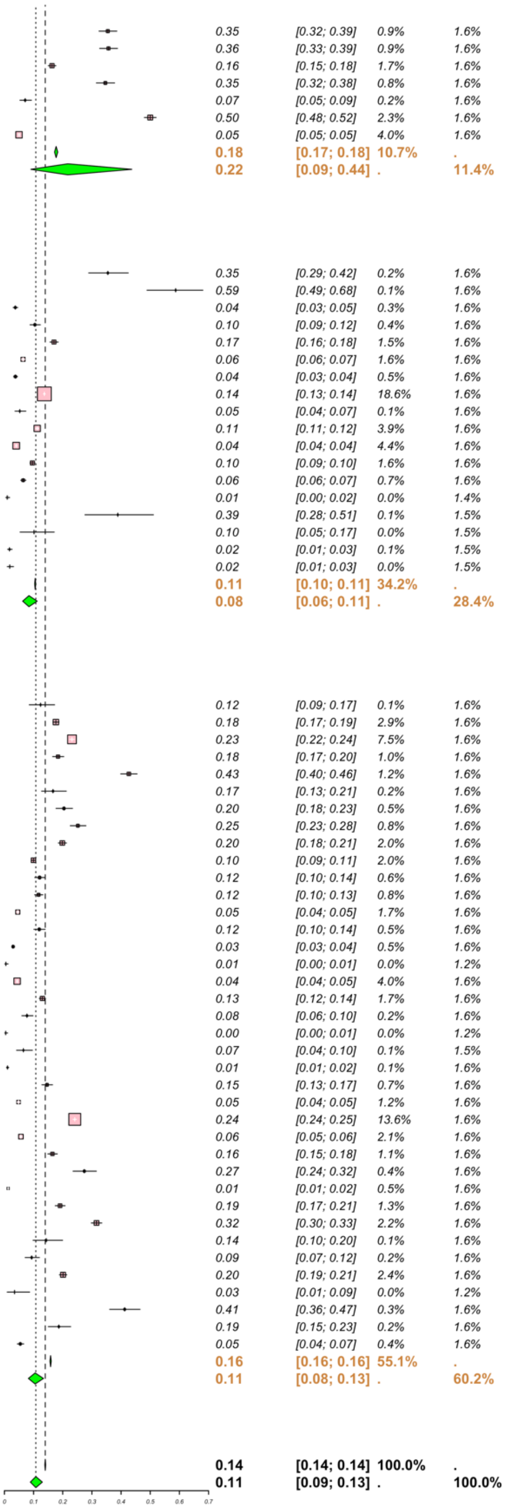

Study	Events	Total	Proportion	95%-CI	Weight (common)	Weight (random)
**subgroup = Case-control**						
Dec 2020 - Dec 2021; 7 states, India (George)	345	973	0.35	[0.32; 0.39]	0.9%	1.6%
May - Dec 2020; 5 US states (Chea)	345	967	0.36	[0.33; 0.39]	0.9%	1.6%
May - Jul 2020; Delhi, India (Dev)	506	3100	0.16	[0.15; 0.18]	1.7%	1.6%
Jun - Jul 2020; Cali, Colombia (Rodriguez-Lopez)	316	913	0.35	[0.32; 0.38]	0.8%	1.6%
Mar - May 2020; Zonguldak, Turkey (Celebi)	50	703	0.07	[0.05; 0.09]	0.2%	1.6%
Nov 2020 - Jan 2021; three regions, Ghana (Lartey)	1201	2402	0.50	[0.48; 0.52]	2.3%	1.6%
Apr - May 2020; multi-site, India (Chatterjee)	1073	21402	0.05	[0.05; 0.05]	4.0%	1.6%
**Fixed effects model**		**30460**	**0.18**	**[0.17; 0.18]**	**10.7%**	**.**
**Random effects model**			**0.22**	**[0.09; 0.44]**	**.**	**11.4%**
Heterogeneity: $I^2 = 100\%$, $p < 0.001$						
Test for effect in subgroup (common effect): z = NA (p = NA)						
Test for effect in subgroup (random effects): z = NA (p = NA)						
**subgroup = Cohort**						
Aug 2020 - May 2021; Atlanta, Georgia, USA (Dube)	72	203	0.35	[0.29; 0.42]	0.2%	1.6%
Aug 2020 - Jan 2021; Mexico city, Mexico (Bueno-Hernández)	64	109	0.59	[0.49; 0.68]	0.1%	1.6%
May 2020 - Jan 2021; Bay area, USA (Doernberg)	91	2435	0.04	[0.03; 0.05]	0.3%	1.6%
Mar - Dec 2020; Western Norway (Bansal)	127	1214	0.10	[0.09; 0.12]	0.4%	1.6%
Jun - Aug 2020; Southeastern Switzerland (Szajek)	475	2794	0.17	[0.16; 0.18]	1.5%	1.6%
May - Aug 2020; Connecticut, USA (Papasavas)	433	6774	0.06	[0.06; 0.07]	1.6%	1.6%
May - Jul 2020; Rennes, France (Paris)	122	3234	0.04	[0.03; 0.04]	0.5%	1.6%
Apr - Jun 2020; NYC, USA (Moscola)	5523	40329	0.14	[0.13; 0.14]	18.6%	1.6%
Mar - Jun 2020; Bergen, Norway (Trieu)	32	607	0.05	[0.04; 0.07]	0.1%	1.6%
Mar - Jun 2020; Oxford, UK (Eyre)	1128	10034	0.11	[0.11; 0.12]	3.9%	1.6%
Apr 2020; Copenhagen, Denmark (Iversen)	1163	28792	0.04	[0.04; 0.04]	4.4%	1.6%
Apr 2020; Ticino, Switzerland (Piccoli)	454	4726	0.10	[0.09; 0.10]	1.6%	1.6%
Apr 2020; Genk, Belgium (Steensels)	197	3056	0.06	[0.06; 0.07]	0.7%	1.6%
Mar - Apr 2020; St. Gallen, Switzerland (Kohler)	10	1012	0.01	[0.00; 0.02]	0.0%	1.4%
Dec 2019 - Jan 2020; Wuhan, China (Ran)	28	72	0.39	[0.28; 0.51]	0.1%	1.5%
Dec 2019 - Jan 2020; Wuhan, China (Wang)	12	118	0.10	[0.05; 0.17]	0.0%	1.5%
Mar - Jul 2020; Hamburg, Germany (Brehm)	22	1253	0.02	[0.01; 0.03]	0.1%	1.5%
Nov - Dec 2020; Victoria, Australia (Lau)	13	706	0.02	[0.01; 0.03]	0.0%	1.5%
**Fixed effects model**		**107468**	**0.11**	**[0.10; 0.11]**	**34.2%**	**.**
**Random effects model**			**0.08**	**[0.06; 0.11]**	**.**	**28.4%**
Heterogeneity: $I^2 = 99\%$, $p < 0.001$						
Test for effect in subgroup (common effect): z = NA (p = NA)						
Test for effect in subgroup (random effects): z = NA (p = NA)						
**subgroup = Cross□sectional**						
Sep 2020 - Dec 2021; Kolkata, India (Chandra)	30	242	0.12	[0.09; 0.17]	0.1%	1.6%
Apr 2021; Dublin & Galway, Ireland (Allen)	898	5085	0.18	[0.17; 0.19]	2.9%	1.6%
Dec 2020 - Mar 2021; nationwide, UK (Martin)	2496	10772	0.23	[0.22; 0.24]	7.5%	1.6%
Nov 2020 - Feb 2021; Nairobi, Kenya (Maina)	300	1631	0.18	[0.17; 0.20]	1.0%	1.6%
Jan 2021; Krakow, Poland (Żółtowska)	521	1221	0.43	[0.40; 0.46]	1.2%	1.6%
Apr 2020 - Jan 2021; Adamawa, Nigeria (Stephen)	53	318	0.17	[0.13; 0.21]	0.2%	1.6%
Nov - Dec 2020; 2 sites, Slovenia (Socan)	166	813	0.20	[0.18; 0.23]	0.5%	1.6%
Dec 2020; 3 regions, Poland (Rosińska)	262	1040	0.25	[0.23; 0.28]	0.8%	1.6%
Aug - Nov 2020; Multicentric, India (Ghosh)	646	3253	0.20	[0.18; 0.21]	2.0%	1.6%
Oct 2020; Dublin & Galway, Ireland (Allen)	576	5787	0.10	[0.09; 0.11]	2.0%	1.6%
Aug - Sep 2020; New Delhi, India (Sharma)	169	1401	0.12	[0.10; 0.14]	0.6%	1.6%
Jul - Sep 2020; Quebec, Canada (Brousseau)	241	2056	0.12	[0.10; 0.13]	0.8%	1.6%
Jul - Aug 2020; Rhode Island, USA (Akinbami)	452	9864	0.05	[0.04; 0.05]	1.7%	1.6%
Jul - Aug 2020; Kolkata, India (Goenka)	134	1122	0.12	[0.10; 0.14]	0.5%	1.6%
Jun - Aug 2020; Northern/Eastern Switzerland (Kahlert)	139	4664	0.03	[0.03; 0.04]	0.5%	1.6%
Jun - Aug 2020; Western Australia (Ludewick)	4	799	0.01	[0.00; 0.01]	0.0%	1.2%
Apr - Aug 2020; 3 US states (Jacob)	1080	24749	0.04	[0.04; 0.05]	4.0%	1.6%
Jun - Jul 2020; New Delhi, India (Gupta)	487	3739	0.13	[0.12; 0.14]	1.7%	1.6%
Jun 2020; Mumbai, India (Mutha)	62	801	0.08	[0.06; 0.10]	0.2%	1.6%
Jun 2020; Osaka, Japan (Nishida)	4	925	0.00	[0.00; 0.01]	0.0%	1.2%
May - Jun 2020; Atlanta, USA (Howard-Anderson)	23	353	0.07	[0.04; 0.10]	0.1%	1.5%
May - Jun 2020; California, USA (Brant-Zawadzki)	31	2924	0.01	[0.01; 0.02]	0.1%	1.6%
May - Jun 2020; Brussels, Belgium (Blairon)	217	1485	0.15	[0.13; 0.17]	0.7%	1.6%
May - Jun 2020; Chicago, USA (Wilkins)	316	6510	0.05	[0.04; 0.05]	1.2%	1.6%
Apr - Jun 2020, New York, USA (Bryan)	4610	19107	0.24	[0.24; 0.25]	13.6%	1.6%
Apr - Jun 2020; Atlanta, USA (Baker)	582	10275	0.06	[0.05; 0.06]	2.1%	1.6%
Apr - Jun 2020; Tehran, Iran (Darvishian)	340	2065	0.16	[0.15; 0.18]	1.1%	1.6%
Mar - May 2020; NYC, USA (Venugopal)	137	500	0.27	[0.24; 0.32]	0.4%	1.6%
Apr - May 2020; San Francisco, USA (Rosser)	136	10449	0.01	[0.01; 0.02]	0.5%	1.6%
Apr - May 2020; Danderyd, Sweden (Rudberg)	410	2149	0.19	[0.17; 0.21]	1.3%	1.6%
Apr 2020; Madrid, Spain (Galan)	818	2590	0.32	[0.30; 0.33]	2.2%	1.6%
Apr 2020; Milan, Italy (Sotgiu)	29	202	0.14	[0.10; 0.20]	0.1%	1.6%
Mar - Apr 2020; Barcelona, Spain (Garcia-Basteiro)	54	578	0.09	[0.07; 0.12]	0.2%	1.6%
Mar - Apr 2020; Alicante, Spain (Algado-Selles)	784	3900	0.20	[0.19; 0.21]	2.4%	1.6%
Feb - Mar 2020; Naples, Italy (Fusco)	4	115	0.03	[0.01; 0.09]	0.0%	1.2%
Jul - Aug 2020; Bukavu, DRC (Mukwege)	148	359	0.41	[0.36; 0.47]	0.3%	1.6%
May - Aug 2020; Amhara, Ethiopia (Atnafie)	78	418	0.19	[0.15; 0.23]	0.2%	1.6%
Jul 2020; Boston, USA (Kataria)	95	1743	0.05	[0.04; 0.07]	0.4%	1.6%
**Fixed effects model**		**146004**	**0.16**	**[0.16; 0.16]**	**55.1%**	**.**
**Random effects model**			**0.11**	**[0.08; 0.13]**	**.**	**60.2%**
Heterogeneity: $I^2 = 100\%$, $p < 0.001$						
Test for effect in subgroup (common effect): z = NA (p = NA)						
Test for effect in subgroup (random effects): z = NA (p = NA)						
**Fixed effects model**		**283932**	**0.14**	**[0.14; 0.14]**	**100.0%**	**.**
**Random effects model**			**0.11**	**[0.09; 0.13]**	**.**	**100.0%**

Heterogeneity: $I^2 = 100\%$, $p < 0.001$
Test for subgroup differences (common effect): $\chi^2_2 = 1413.60$, df = 2 ($p < 0.001$)
Test for subgroup differences (random effects): $\chi^2_2 = 4.58$, df = 2 ($p = 0.10$)

Supplementary figure 4: Funnel and Doi plots for meta-analysis involving SARS-CoV-2 infection rate estimation

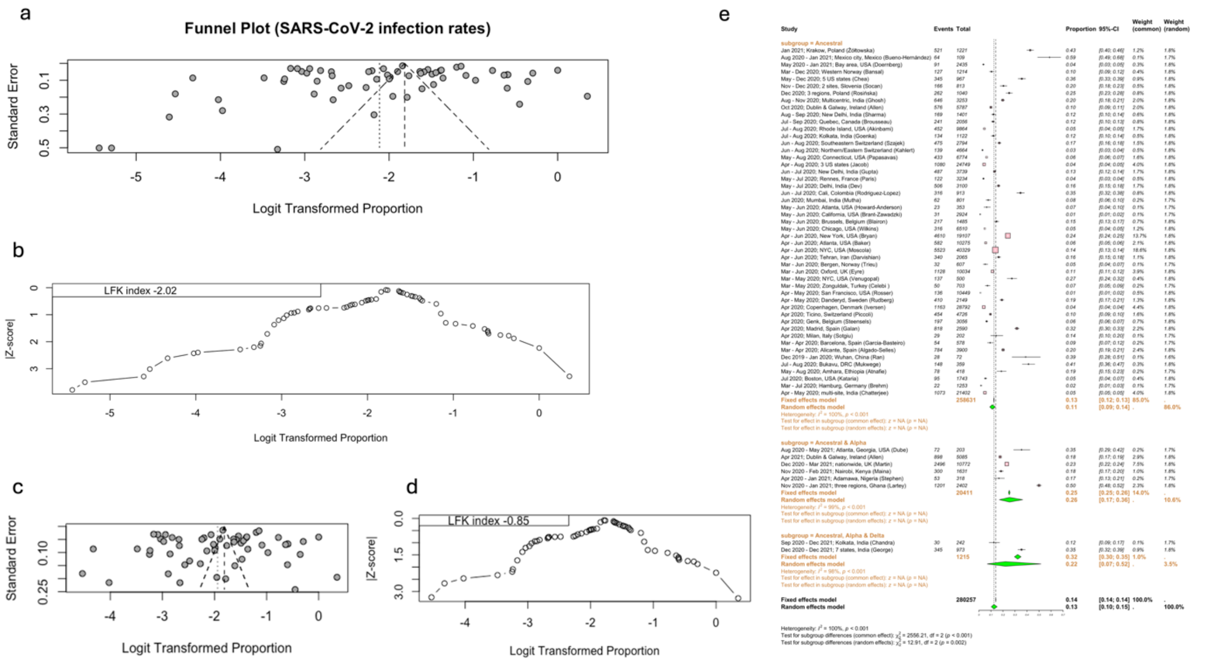

Supplementary figure 4a and 4b visualise funnel and Doi plots respectively. Funnel plots are used to assess publication bias, while Doi plots visualise and quantify the asymmetry of study effects in meta-analyses examining SARS-CoV-2 infection rates in healthcare workers (HCWs). Supplementary figure 4c-e visualise funnel plot, Doi plot and infection rate following a trim-and-fill method to adjust for potential biases in infection rate meta-analysis. In order to obtain LFK index within acceptable limits, we removed studies (numbers 23, 31, 52, 54, 52, 56, 63; refer to Supplementary Table 5) based on high standard error of effect estimate. The overall infection rate remains comparable for the alpha and delta variants. However, it is 2% higher for the ancestral strain or when subgroup analysis is not performed.

Supplementary figure 5: Occupational exposure to SARS-CoV-2 by study region or continent

Study or subgroup	Exposed Infected	Exposed Total	Not exposed Infected	Not exposed Total	OR	95%-CI	Weight (common)	Weight (random)
**Subgroup = Africa**								
Nov 2020 - Feb 2021; Nairobi, Kenya (Maina)	49	209	37	234	1.63	[1.01; 2.62]	0.8%	1.9%
Apr 2020 - Jan 2021; Adamawa, Nigeria (Stephen)	6	42	27	193	1.02	[0.39; 2.66]	0.2%	1.4%
**Fixed effects model**		**251**		**427**	**1.49**	**[0.98; 2.27]**	**1.0%**	**.**
**Random effects model**					**1.49**	**[0.97; 2.28]**	**.**	**3.3%**
Heterogeneity: $I^2 = 0\%$, $p = 0.39$								
Test for effect in subgroup (common effect): $z = 1.85$ ($p = 0.06$)								
Test for effect in subgroup (random effects): $z = 1.83$ ($p = 0.07$)								
**Subgroup = Asia**								
Sep 2020 - Dec 2021; Kolkata, India (Chandra)	14	76	16	166	2.12	[0.97; 4.60]	0.2%	1.6%
Dec 2020 - Dec 2021; 7 states, India (George)	273	746	72	227	1.24	[0.91; 1.71]	2.1%	2.1%
Aug - Nov 2020; Multicentric, India (Ghosh)	411	1922	235	1331	1.27	[1.06; 1.52]	6.4%	2.2%
Aug - Sep 2020; New Delhi, India (Sharma)	49	466	120	935	0.80	[0.56; 1.14]	2.1%	2.1%
Jul - Aug 2020; Kolkata, India (Goenka)	27	136	6	75	2.85	[1.12; 7.25]	0.2%	1.4%
Jun - Jul 2020; New Delhi, India (Gupta)	110	943	377	2796	0.85	[0.68; 1.06]	5.0%	2.2%
May - Jul 2020; Delhi, India (Dev)	217	313	289	446	1.23	[0.90; 1.67]	2.2%	2.1%
Jun 2020; Mumbai, India (Mutha)	39	343	6	175	3.61	[1.50; 8.71]	0.2%	1.5%
Jun 2020; Osaka, Japan (Nishida)	2	267	2	658	2.48	[0.35; 17.66]	0.0%	0.6%
Apr - Jun 2020; Tehran, Iran (Darvishian)	287	1632	53	433	1.53	[1.12; 2.10]	2.0%	2.1%
Mar - May 2020; Zonguldak, Turkey (Celebi)	44	527	6	176	2.58	[1.08; 6.17]	0.2%	1.5%
Dec 2019 - Jan 2020; Wuhan, China (Ran)	18	33	10	39	3.48	[1.29; 9.39]	0.1%	1.3%
Dec 2019 - Jan 2020; Wuhan, China (Wang)	7	96	5	22	0.27	[0.08; 0.94]	0.2%	1.1%
**Fixed effects model**		**7500**		**7479**	**1.21**	**[1.09; 1.33]**	**21.0%**	**.**
**Random effects model**					**1.41**	**[1.05; 1.88]**	**.**	**21.8%**
Heterogeneity: $I^2 = 71\%$, $p < 0.001$								
Test for effect in subgroup (common effect): $z = 3.71$ ($p < 0.001$)								
Test for effect in subgroup (random effects): $z = 2.32$ ($p = 0.02$)								
**Subgroup = Europe**								
Apr 2021; Dublin & Galway, Ireland (Allen)	265	1136	170	1449	2.29	[1.85; 2.83]	3.4%	2.2%
Dec 2020 - Mar 2021; nationwide, UK (Martin)	275	687	1030	6298	3.41	[2.89; 4.03]	3.6%	2.2%
Jan 2021; Krakow, Poland (Żółtowska)	364	827	157	394	1.19	[0.93; 1.51]	3.5%	2.2%
Mar - Dec 2020; Western Norway (Bansal)	63	409	11	225	3.54	[1.83; 6.87]	0.4%	1.7%
Nov - Dec 2020; 2 sites, Slovenia (Socan)	140	612	26	198	1.96	[1.25; 3.09]	0.9%	2.0%
Dec 2020; 3 regions, Poland (Rosińska)	74	228	92	516	2.21	[1.55; 3.17]	1.1%	2.1%
Oct 2020; Dublin & Galway, Ireland (Allen)	136	902	96	1635	2.85	[2.16; 3.75]	1.7%	2.1%
Jun - Aug 2020; Southeastern Switzerland (Szajek)	46	701	18	845	3.23	[1.85; 5.62]	0.4%	1.9%
Jun - Aug 2020; Northern/Eastern Switzerland (Kahlert)	85	2062	40	2348	2.48	[1.70; 3.63]	1.1%	2.0%
May - Jul 2020; Rennes, France (Paris)	75	1881	37	1151	1.25	[0.84; 1.87]	1.3%	2.0%
May - Jun 2020; Brussels, Belgium (Blairon)	74	550	74	588	1.08	[0.76; 1.53]	1.8%	2.1%
Mar - Jun 2020; Bergen, Norway (Trieu)	9	370	3	216	1.77	[0.47; 6.61]	0.1%	1.0%
Mar - Jun 2020; Oxford, UK (Eyre)	358	1586	631	7369	3.11	[2.70; 3.59]	5.1%	2.2%
Apr - May 2020; Danderyd, Sweden (Rudberg)	228	962	26	305	3.33	[2.17; 5.12]	0.9%	2.0%
Apr 2020; Copenhagen, Denmark (Iversen)	506	18484	104	4363	1.15	[0.93; 1.43]	4.8%	2.2%
Apr 2020; Ticino, Switzerland (Piccoli)	94	491	360	4325	2.61	[2.03; 3.35]	1.7%	2.2%
Apr 2020; Genk, Belgium (Steensels)	73	1092	120	1921	1.08	[0.80; 1.45]	2.4%	2.1%
Apr 2020; Madrid, Spain (Galan)	450	1359	48	201	1.58	[1.12; 2.22]	1.6%	2.1%
Apr 2020; Milan, Italy (Sotgiu)	22	158	7	44	0.86	[0.34; 2.16]	0.3%	1.4%
Mar - Apr 2020; Barcelona, Spain (Garcia-Basteiro)	23	263	31	315	0.88	[0.50; 1.55]	0.8%	1.8%
Mar - Apr 2020; St. Gallen, Switzerland (Kohler)	1	101	2	108	0.53	[0.05; 5.94]	0.1%	0.5%
Mar - Apr 2020; Alicante, Spain (Algado-Selles)	119	523	27	182	1.69	[1.07; 2.67]	0.9%	2.0%
Feb - Mar 2020; Naples, Italy (Fusco)	4	107	0	8	0.74	[0.04; 14.91]	0.0%	0.3%
**Fixed effects model**		**35491**		**35004**	**2.08**	**[1.95; 2.22]**	**37.9%**	**.**
**Random effects model**					**1.88**	**[1.54; 2.30]**	**.**	**42.2%**
Heterogeneity: $I^2 = 88\%$, $p < 0.001$								
Test for effect in subgroup (common effect): $z = 22.33$ ($p < 0.001$)								
Test for effect in subgroup (random effects): $z = 6.21$ ($p < 0.001$)								
**Subgroup = North America**								
Aug 2020 - May 2021; Atlanta, Georgia, USA (Dube)	32	84	40	119	1.22	[0.68; 2.17]	0.6%	1.8%
Aug 2020 - Jan 2021; Mexico city, Mexico (Bueno-Hernández)	37	57	5	10	1.85	[0.48; 7.16]	0.1%	1.0%
May 2020 - Jan 2021; Bay area, USA (Doernberg)	34	797	19	680	1.55	[0.88; 2.74]	0.6%	1.8%
May - Dec 2020; 5 US states (Chea)	113	310	232	630	0.98	[0.74; 1.31]	2.9%	2.1%
Jul - Sep 2020; Quebec, Canada (Brousseau)	114	561	36	742	5.00	[3.38; 7.41]	0.7%	2.0%
Jul - Aug 2020; Rhode Island, USA (Akinbami)	315	4618	137	5245	2.73	[2.22; 3.35]	3.5%	2.2%
May - Aug 2020; Connecticut, USA (Papasavas)	113	880	318	5941	2.61	[2.07; 3.27]	2.1%	2.2%
Apr - Aug 2020; 3 US states (Jacob)	584	12413	448	11435	1.21	[1.07; 1.37]	13.1%	2.2%
May - Jun 2020; Atlanta, USA (Howard-Anderson)	4	32	19	317	2.24	[0.71; 7.05]	0.1%	1.2%
May - Jun 2020; California, USA (Brant-Zawadzki)	19	1608	6	775	1.53	[0.61; 3.85]	0.2%	1.4%
May - Jun 2020; Chicago, USA (Wilkins)	180	2419	59	2092	2.77	[2.05; 3.74]	1.7%	2.1%
Apr - Jun 2020, New York, USA (Bryan)	58	243	34	89	0.51	[0.30; 0.85]	1.1%	1.9%
Apr - Jun 2020; Atlanta, USA (Baker)	223	3561	332	6049	1.15	[0.97; 1.37]	6.8%	2.2%
Apr - Jun 2020; NYC, USA (Moscola)	2726	4604	1177	22155	25.87	[23.81; 28.11]	4.9%	2.2%
Mar - May 2020; NYC, USA (Venugopal)	25	65	105	413	1.83	[1.06; 3.17]	0.5%	1.9%
Apr - May 2020; San Francisco, USA (Rosser)	20	1314	77	8344	1.66	[1.01; 2.72]	0.6%	1.9%
**Fixed effects model**		**33566**		**65036**	**4.57**	**[4.34; 4.81]**	**39.6%**	**.**
**Random effects model**					**2.03**	**[1.29; 3.19]**	**.**	**30.3%**
Heterogeneity: $I^2 = 99\%$, $p < 0.001$								
Test for effect in subgroup (common effect): $z = 58.15$ ($p < 0.001$)								
Test for effect in subgroup (random effects): $z = 3.06$ ($p = 0.002$)								
**Subgroup = Oceania**								
Jun - Aug 2020; Western Australia (Ludewick)	2	337	2	462	1.37	[0.19; 9.80]	0.0%	0.6%
**Subgroup = South America**								
Jun - Jul 2020; Cali, Colombia (Rodriguez-Lopez)	59	101	29	58	1.40	[0.73; 2.69]	0.5%	1.7%
**Fixed effects model**		**77246**		**108466**	**2.87**	**[2.77; 2.98]**	**100.0%**	**.**
**Random effects model**					**1.79**	**[1.49; 2.14]**	**.**	**100.0%**

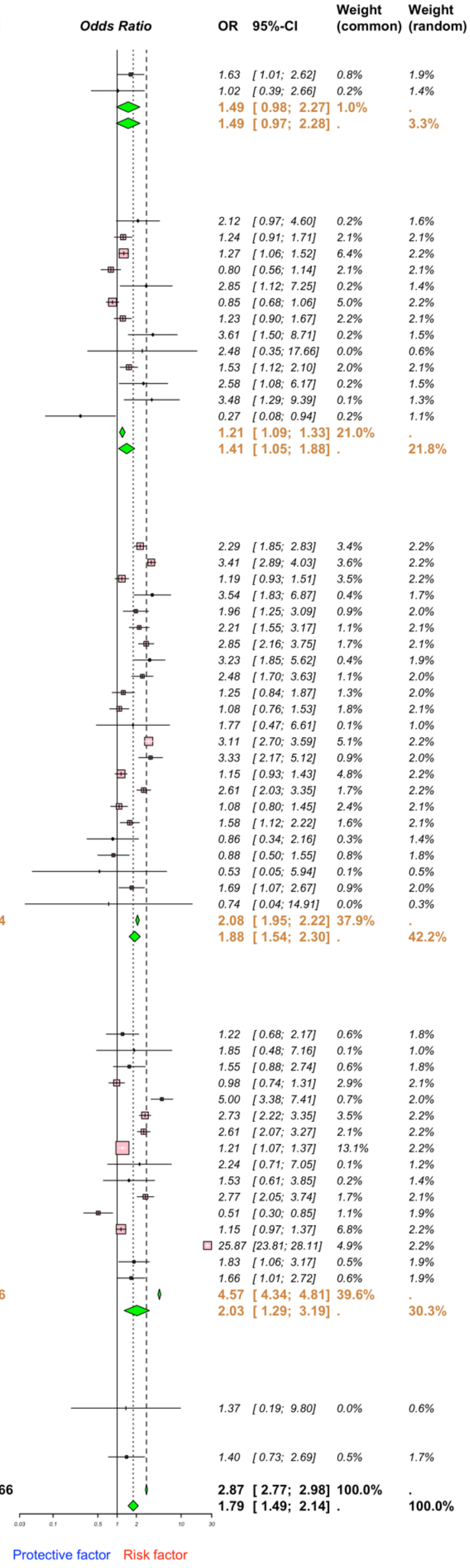

Heterogeneity: $I^2 = 99\%$, $p < 0.001$
Test for overall effect (common effect): $z = 56.96$ ($p < 0.001$)
Test for overall effect (random effects): $z = 6.22$ ($p < 0.001$)
Test for subgroup differences (common effect): $\chi^2_5 = 719.85$, df = 5 ($p < 0.001$)
Test for subgroup differences (random effects): $\chi^2_5 = 4.01$, df = 5 ($p = 0.55$)

Supplementary figure 6: Occupational exposure to SARS-CoV-2 by study design

Study or subgroup	Exposed Infected	Exposed Total	Not exposed Infected	Not exposed Total	OR	95%-CI	Weight (common)	Weight (random)
**Subgroup = Case-control**								
Dec 2020 - Dec 2021; 7 states, India (George)	273	746	72	227	1.24	[0.91; 1.71]	2.1%	2.1%
May - Dec 2020; 5 US states (Chea)	113	310	232	630	0.98	[0.74; 1.31]	2.9%	2.1%
May - Jul 2020; Delhi, India (Dev)	217	313	289	446	1.23	[0.90; 1.67]	2.2%	2.1%
Jun - Jul 2020; Cali, Colombia (Rodriguez-Lopez)	59	101	29	58	1.40	[0.73; 2.69]	0.5%	1.7%
Mar - May 2020; Zonguldak, Turkey (Celebi)	44	527	6	176	2.58	[1.08; 6.17]	0.2%	1.5%
**Fixed effects model**		**1997**		**1537**	**1.19**	**[1.01; 1.41]**	**7.8%**	**.**
**Random effects model**					**1.18**	**[1.00; 1.40]**	**.**	**9.6%**
Heterogeneity: $I^2 = 22\%$, $p = 0.27$								
Test for effect in subgroup (common effect): $z = 2.13$ ($p = 0.03$)								
Test for effect in subgroup (random effects): $z = 1.99$ ($p = 0.05$)								
**Subgroup = Cohort**								
Aug 2020 - May 2021; Atlanta, Georgia, USA (Dube)	32	84	40	119	1.22	[0.68; 2.17]	0.6%	1.8%
Aug 2020 - Jan 2021; Mexico city, Mexico (Bueno-Hernández)	37	57	5	10	1.85	[0.48; 7.16]	0.1%	1.0%
May 2020 - Jan 2021; Bay area, USA (Doernberg)	34	797	19	680	1.55	[0.88; 2.74]	0.6%	1.8%
Mar - Dec 2020; Western Norway (Bansal)	63	409	11	225	3.54	[1.83; 6.87]	0.4%	1.7%
Jun - Aug 2020; Southeastern Switzerland (Szajek)	46	701	18	845	3.23	[1.85; 5.62]	0.4%	1.9%
May - Aug 2020; Connecticut, USA (Papasavas)	113	880	318	5941	2.61	[2.07; 3.27]	2.1%	2.2%
May - Jul 2020; Rennes, France (Paris)	75	1881	37	1151	1.25	[0.84; 1.87]	1.3%	2.0%
Apr - Jun 2020; NYC, USA (Moscola)	2726	4604	1177	22155	25.87	[23.81; 28.11]	4.9%	2.2%
Mar - Jun 2020; Bergen, Norway (Trieu)	9	370	3	216	1.77	[0.47; 6.61]	0.1%	1.0%
Mar - Jun 2020; Oxford, UK (Eyre)	358	1586	631	7369	3.11	[2.70; 3.59]	5.1%	2.2%
Apr 2020; Copenhagen, Denmark (Iversen)	506	18484	104	4363	1.15	[0.93; 1.43]	4.8%	2.2%
Apr 2020; Ticino, Switzerland (Piccoli)	94	491	360	4325	2.61	[2.03; 3.35]	1.7%	2.2%
Apr 2020; Genk, Belgium (Steensels)	73	1092	120	1921	1.08	[0.80; 1.45]	2.4%	2.1%
Mar - Apr 2020; St. Gallen, Switzerland (Kohler)	1	101	2	108	0.53	[0.05; 5.94]	0.1%	0.5%
Dec 2019 - Jan 2020; Wuhan, China (Ran)	18	33	10	39	3.48	[1.29; 9.39]	0.1%	1.3%
Dec 2019 - Jan 2020; Wuhan, China (Wang)	7	96	5	22	0.27	[0.08; 0.94]	0.2%	1.1%
**Fixed effects model**		**31666**		**49489**	**6.69**	**[6.31; 7.10]**	**24.9%**	**.**
**Random effects model**					**2.10**	**[1.29; 3.41]**	**.**	**27.3%**
Heterogeneity: $I^2 = 99\%$, $p < 0.001$								
Test for effect in subgroup (common effect): $z = 63.78$ ($p < 0.001$)								
Test for effect in subgroup (random effects): $z = 2.99$ ($p = 0.003$)								
**Subgroup = Cross-sectional**								
Sep 2020 - Dec 2021; Kolkata, India (Chandra)	14	76	16	166	2.12	[0.97; 4.60]	0.2%	1.6%
Apr 2021; Dublin & Galway, Ireland (Allen)	265	1136	170	1449	2.29	[1.85; 2.83]	3.4%	2.2%
Dec 2020 - Mar 2021; nationwide, UK (Martin)	275	687	1030	6298	3.41	[2.89; 4.03]	3.6%	2.2%
Nov 2020 - Feb 2021; Nairobi, Kenya (Maina)	49	209	37	234	1.63	[1.01; 2.62]	0.8%	1.9%
Jan 2021; Krakow, Poland (Żółtowska)	364	827	157	394	1.19	[0.93; 1.51]	3.5%	2.2%
Apr 2020 - Jan 2021; Adamawa, Nigeria (Stephen)	6	42	27	193	1.02	[0.39; 2.66]	0.2%	1.4%
Nov - Dec 2020; 2 sites, Slovenia (Socan)	140	612	26	198	1.96	[1.25; 3.09]	0.9%	2.0%
Dec 2020; 3 regions, Poland (Rosińska)	74	228	92	516	2.21	[1.55; 3.17]	1.1%	2.1%
Aug - Nov 2020; Multicentric, India (Ghosh)	411	1922	235	1331	1.27	[1.06; 1.52]	6.4%	2.2%
Oct 2020; Dublin & Galway, Ireland (Allen)	136	902	96	1635	2.85	[2.16; 3.75]	1.7%	2.1%
Aug - Sep 2020; New Delhi, India (Sharma)	49	466	120	935	0.80	[0.56; 1.14]	2.1%	2.1%
Jul - Sep 2020; Quebec, Canada (Brousseau)	114	561	36	742	5.00	[3.38; 7.41]	0.7%	2.0%
Jul - Aug 2020; Rhode Island, USA (Akinbami)	315	4618	137	5245	2.73	[2.22; 3.35]	3.5%	2.2%
Jul - Aug 2020; Kolkata, India (Goenka)	27	136	6	75	2.85	[1.12; 7.25]	0.2%	1.4%
Jun - Aug 2020; Northern/Eastern Switzerland (Kahlert)	85	2062	40	2348	2.48	[1.70; 3.63]	1.1%	2.0%
Jun - Aug 2020; Western Australia (Ludewick)	2	337	2	462	1.37	[0.19; 9.80]	0.0%	0.6%
Apr - Aug 2020; 3 US states (Jacob)	584	12413	448	11435	1.21	[1.07; 1.37]	13.1%	2.2%
Jun - Jul 2020; New Delhi, India (Gupta)	110	943	377	2796	0.85	[0.68; 1.06]	5.0%	2.2%
Jun 2020; Mumbai, India (Mutha)	39	343	6	175	3.61	[1.50; 8.71]	0.2%	1.5%
Jun 2020; Osaka, Japan (Nishida)	2	267	2	658	2.48	[0.35; 17.66]	0.0%	0.6%
May - Jun 2020; Atlanta, USA (Howard-Anderson)	4	32	19	317	2.24	[0.71; 7.05]	0.1%	1.2%
May - Jun 2020; California, USA (Brant-Zawadzki)	19	1608	6	775	1.53	[0.61; 3.85]	0.2%	1.4%
May - Jun 2020; Brussels, Belgium (Blairon)	74	550	74	588	1.08	[0.76; 1.53]	1.8%	2.1%
May - Jun 2020; Chicago, USA (Wilkins)	180	2419	59	2092	2.77	[2.05; 3.74]	1.7%	2.1%
Apr - Jun 2020, New York, USA (Bryan)	58	243	34	89	0.51	[0.30; 0.85]	1.1%	1.9%
Apr - Jun 2020; Atlanta, USA (Baker)	223	3561	332	6049	1.15	[0.97; 1.37]	6.8%	2.2%
Apr - Jun 2020; Tehran, Iran (Darvishian)	287	1632	53	433	1.53	[1.12; 2.10]	2.0%	2.1%
Mar - May 2020; NYC, USA (Venugopal)	25	65	105	413	1.83	[1.06; 3.17]	0.5%	1.9%
Apr - May 2020; San Francisco, USA (Rosser)	20	1314	77	8344	1.66	[1.01; 2.72]	0.6%	1.9%
Apr - May 2020; Danderyd, Sweden (Rudberg)	228	962	26	305	3.33	[2.17; 5.12]	0.9%	2.0%
Apr 2020; Madrid, Spain (Galan)	450	1359	48	201	1.58	[1.12; 2.22]	1.6%	2.1%
Apr 2020; Milan, Italy (Sotgiu)	22	158	7	44	0.86	[0.34; 2.16]	0.3%	1.4%
Mar - Apr 2020; Barcelona, Spain (Garcia-Basteiro)	23	263	31	315	0.88	[0.50; 1.55]	0.8%	1.8%
Mar - Apr 2020; Alicante, Spain (Algado-Selles)	119	523	27	182	1.69	[1.07; 2.67]	0.9%	2.0%
Feb - Mar 2020; Naples, Italy (Fusco)	4	107	0	8	0.74	[0.04; 14.91]	0.0%	0.3%
**Fixed effects model**		**43583**		**57440**	**1.65**	**[1.57; 1.74]**	**67.3%**	**.**
**Random effects model**					**1.70**	**[1.42; 2.04]**	**.**	**63.1%**
Heterogeneity: $I^2 = 90\%$, $p < 0.001$								
Test for effect in subgroup (common effect): $z = 19.22$ ($p < 0.001$)								
Test for effect in subgroup (random effects): $z = 5.77$ ($p < 0.001$)								
**Fixed effects model**		**77246**		**108466**	**2.87**	**[2.77; 2.98]**	**100.0%**	**.**
**Random effects model**					**1.79**	**[1.49; 2.14]**	**.**	**100.0%**

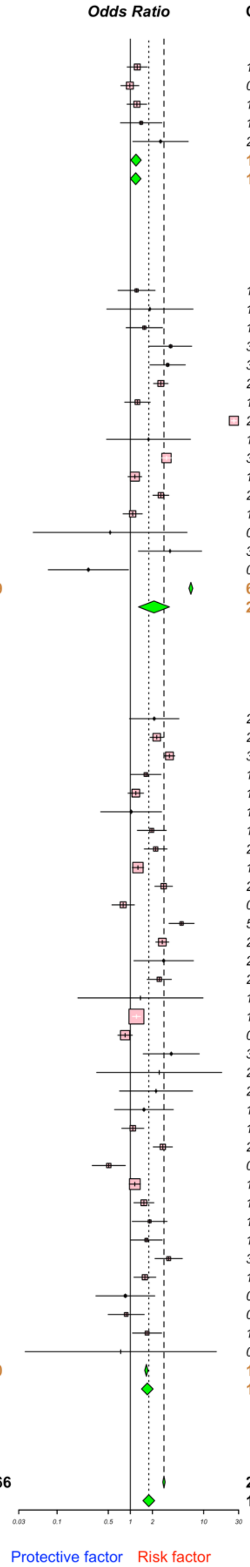

Heterogeneity: $I^2 = 99\%$, $p < 0.001$
Test for overall effect (common effect): $z = 56.96$ ($p < 0.001$)
Test for overall effect (random effects): $z = 6.22$ ($p < 0.001$)
Test for subgroup differences (common effect): $\chi^2_2 = 1361.86$, df = 2 ($p < 0.001$)
Test for subgroup differences (random effects): $\chi^2_2 = 10.98$, df = 2 ($p = 0.004$)

Supplementary figure 7: Funnel and Doi plot for meta-analysis involving occupational exposure to SARS-CoV-2

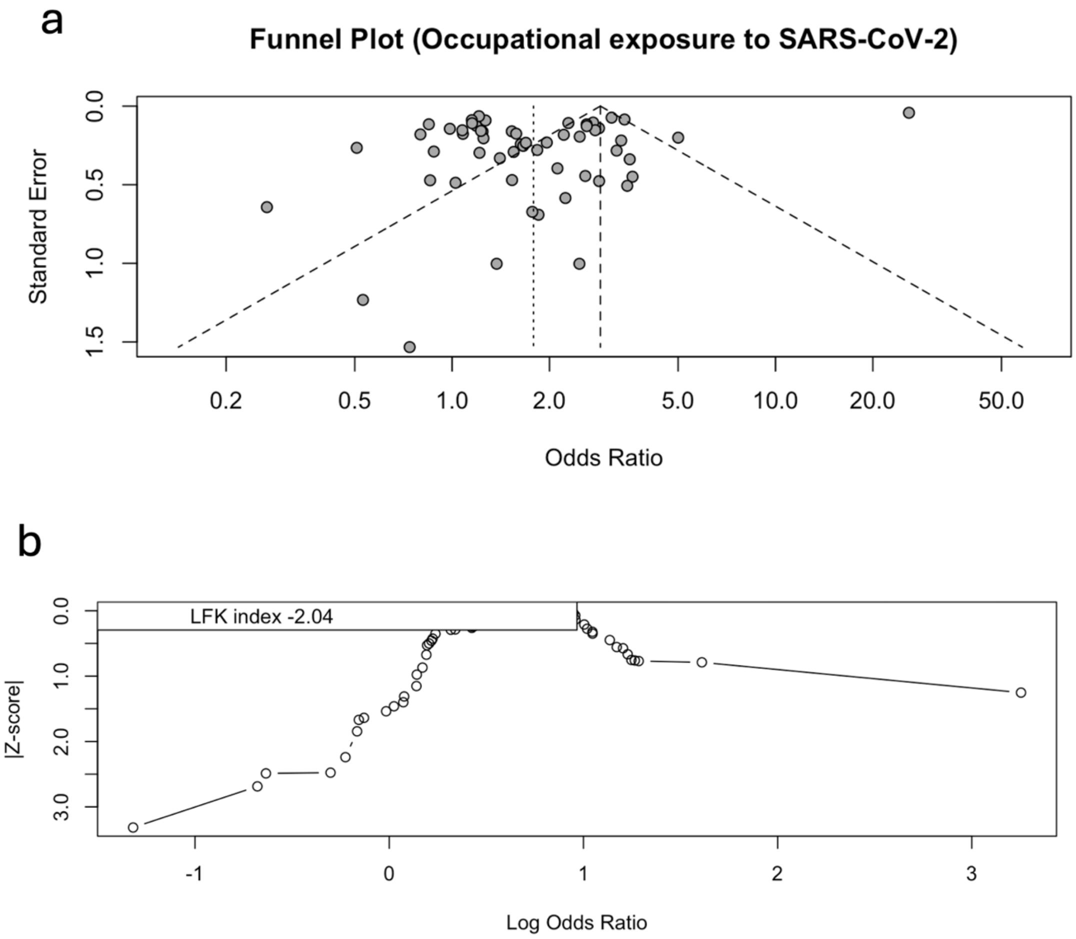

Supplementary figure 7a and 7b visualise funnel and Doi plots respectively. Funnel plots are used to assess publication bias, while Doi plots visualise and quantify the asymmetry of study effects in meta-analyses examining occupational exposure risk factor for SARS-CoV-2 infection in HCWs.

Supplementary figure 8: Occupational exposure to SARS-CoV-2 with insufficient personal protective equipment (PPE) use by study region or continent

Study or subgroup	Insufficient Infected	Insufficient Total	Sufficient Infected	Sufficient Total	OR	95%-CI	Weight (common)	Weight (random)
**Subgroup = Africa**								
Nov 2020 - Jan 2021; three regions, Ghana (Lartey)	477	1072	343	673	0.77	[0.64; 0.94]	14.8%	5.9%
Apr 2020 - Jan 2021; Adamawa, Nigeria (Stephen)	43	208	10	110	2.61	[1.25; 5.42]	0.7%	3.9%
Jul - Aug 2020; Bukavu, DRC (Mukwege)	3	5	48	119	2.22	[0.36; 13.78]	0.1%	1.4%
May - Aug 2020; Amhara, Ethiopia (Atnafie)	47	238	31	180	1.18	[0.72; 1.95]	1.8%	4.8%
**Fixed effects model**		**1523**		**1082**	**0.89**	**[0.75; 1.06]**	**17.3%**	.
**Random effects model**					**1.30**	**[0.70; 2.39]**	.	**16.0%**
Heterogeneity: $I^2 = 76\%$, $p = 0.006$								
Test for effect in subgroup (common effect): $z = -1.31$ ($p = 0.19$)								
Test for effect in subgroup (random effects): $z = 0.83$ ($p = 0.40$)								
**Subgroup = Asia**								
Sep 2020 - Dec 2021; Kolkata, India (Chandra)	2	45	28	197	0.28	[0.06; 1.22]	0.6%	1.9%
Dec 2020 - Dec 2021; 7 states, India (George)	4	14	125	348	0.71	[0.22; 2.32]	0.4%	2.5%
Aug - Nov 2020; Multicentric, India (Ghosh)	71	288	575	2965	1.36	[1.02; 1.80]	4.8%	5.7%
Jun - Jul 2020; New Delhi, India (Gupta)	38	300	193	1473	0.96	[0.66; 1.40]	3.6%	5.3%
Jun 2020; Mumbai, India (Mutha)	13	222	13	130	0.56	[0.25; 1.25]	1.0%	3.7%
Apr - May 2020; multi-site, India (Chatterjee)	57	74	321	677	3.72	[2.12; 6.52]	0.9%	4.6%
Dec 2019 - Jan 2020; Wuhan, China (Ran)	24	49	4	23	4.56	[1.35; 15.37]	0.2%	2.4%
**Fixed effects model**		**992**		**5813**	**1.32**	**[1.10; 1.59]**	**11.6%**	.
**Random effects model**					**1.21**	**[0.63; 2.34]**	.	**26.0%**
Heterogeneity: $I^2 = 80\%$, $p < 0.001$								
Test for effect in subgroup (common effect): $z = 2.92$ ($p = 0.003$)								
Test for effect in subgroup (random effects): $z = 0.57$ ($p = 0.57$)								
**Subgroup = Europe**								
Dec 2020 - Mar 2021; nationwide, UK (Martin)	1634	6182	859	4560	1.55	[1.41; 1.70]	45.9%	6.1%
Mar - Dec 2020; Western Norway (Bansal)	1	12	10	175	1.50	[0.18; 12.80]	0.1%	1.0%
Jun - Aug 2020; Southeastern Switzerland (Szajek)	4	38	42	664	1.74	[0.59; 5.14]	0.3%	2.7%
Jun - Aug 2020; Northern/Eastern Switzerland (Kahlert)	8	47	59	1275	4.23	[1.89; 9.45]	0.2%	3.6%
May - Jul 2020; Rennes, France (Paris)	13	173	43	1140	2.07	[1.09; 3.94]	0.7%	4.3%
Mar - Jul 2020; Hamburg, Germany (Brehm)	6	69	5	226	4.21	[1.24; 14.25]	0.1%	2.4%
Apr 2020; Madrid, Spain (Galan)	219	522	374	1354	1.89	[1.53; 2.34]	7.6%	5.9%
Mar - Apr 2020; St. Gallen, Switzerland (Kohler)	7	234	3	565	5.78	[1.48; 22.54]	0.1%	2.1%
Mar - Apr 2020; Alicante, Spain (Algado-Selles)	112	491	40	293	1.87	[1.26; 2.77]	2.4%	5.3%
**Fixed effects model**		**7768**		**10252**	**1.64**	**[1.51; 1.78]**	**57.5%**	.
**Random effects model**					**1.95**	**[1.57; 2.41]**	.	**33.4%**
Heterogeneity: $I^2 = 47\%$, $p = 0.06$								
Test for effect in subgroup (common effect): $z = 11.82$ ($p < 0.001$)								
Test for effect in subgroup (random effects): $z = 6.12$ ($p < 0.001$)								
**Subgroup = North America**								
Aug 2020 - May 2021; Atlanta, Georgia, USA (Dube)	6	20	66	183	0.76	[0.28; 2.07]	0.6%	3.0%
May - Dec 2020; 5 US states (Chea)	67	177	38	97	0.95	[0.57; 1.57]	1.9%	4.8%
Jul - Aug 2020; Rhode Island, USA (Akinbami)	88	1490	227	3128	0.80	[0.62; 1.03]	8.7%	5.7%
Jul 2020; Boston, USA (Kataria)	0	12	85	1549	0.69	[0.04; 11.67]	0.1%	0.6%
May - Jun 2020; Atlanta, USA (Howard-Anderson)	3	74	19	242	0.50	[0.14; 1.73]	0.5%	2.3%
Apr - Jun 2020; Atlanta, USA (Baker)	14	221	370	6320	1.09	[0.63; 1.89]	1.5%	4.6%
Mar - May 2020; NYC, USA (Venugopal)	1	2	90	361	3.01	[0.19; 48.64]	0.0%	0.7%
**Fixed effects model**		**1996**		**11880**	**0.84**	**[0.69; 1.03]**	**13.3%**	.
**Random effects model**					**0.85**	**[0.69; 1.04]**	.	**21.8%**
Heterogeneity: $I^2 = 0\%$, $p = 0.84$								
Test for effect in subgroup (common effect): $z = -1.64$ ($p = 0.10$)								
Test for effect in subgroup (random effects): $z = -1.58$ ($p = 0.12$)								
**Subgroup = South America**								
Jun - Jul 2020; Cali, Colombia (Rodriguez-Lopez)	13	18	97	205	2.89	[1.00; 8.42]	0.3%	2.8%
**Fixed effects model**		**12297**		**29232**	**1.37**	**[1.28; 1.46]**	**100.0%**	.
**Random effects model**					**1.45**	**[1.14; 1.84]**	.	**100.0%**

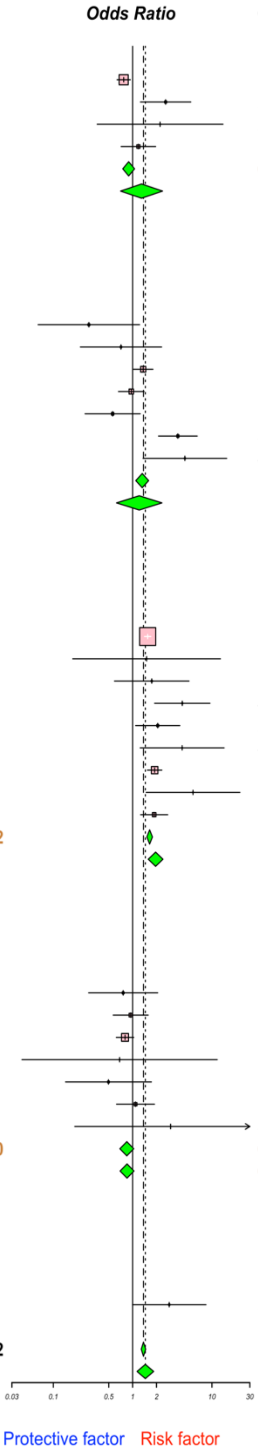

Heterogeneity: $I^2 = 79\%$, $p < 0.001$
Test for overall effect (common effect): $z = 9.60$ ($p < 0.001$)
Test for overall effect (random effects): $z = 3.00$ ($p = 0.003$)
Test for subgroup differences (common effect): $\chi^2_4 = 66.42$, df = 4 ($p < 0.001$)
Test for subgroup differences (random effects): $\chi^2_4 = 32.90$, df = 4 ($p < 0.001$)

Supplementary figure 9: Occupational exposure to SARS-CoV-2 with insufficient PPE use by study design

Study or subgroup	Insufficient Infected	Insufficient Total	Sufficient Infected	Sufficient Total	OR	95%-CI	Weight (common)	Weight (random)
**Subgroup = Case-control**								
Dec 2020 - Dec 2021; 7 states, India (George)	4	14	125	348	0.71	[0.22; 2.32]	0.4%	2.5%
Nov 2020 - Jan 2021; three regions, Ghana (Lartey)	477	1072	343	673	0.77	[0.64; 0.94]	14.8%	5.9%
May - Dec 2020; 5 US states (Chea)	67	177	38	97	0.95	[0.57; 1.57]	1.9%	4.8%
Jun - Jul 2020; Cali, Colombia (Rodriguez-Lopez)	13	18	97	205	2.89	[1.00; 8.42]	0.3%	2.8%
Apr - May 2020; multi-site, India (Chatterjee)	57	74	321	677	3.72	[2.12; 6.52]	0.9%	4.6%
**Fixed effects model**		**1355**		**2000**	**0.97**	**[0.82; 1.14]**	**18.3%**	**.**
**Random effects model**					**1.39**	**[0.70; 2.78]**	**.**	**20.6%**
Heterogeneity: $I^2 = 87\%$, $p < 0.001$								
Test for effect in subgroup (common effect): $z = -0.39$ ($p = 0.69$)								
Test for effect in subgroup (random effects): $z = 0.93$ ($p = 0.35$)								
**Subgroup = Cohort**								
Aug 2020 - May 2021; Atlanta, Georgia, USA (Dube)	6	20	66	183	0.76	[0.28; 2.07]	0.6%	3.0%
Mar - Dec 2020; Western Norway (Bansal)	1	12	10	175	1.50	[0.18; 12.80]	0.1%	1.0%
Jun - Aug 2020; Southeastern Switzerland (Szajek)	4	38	42	664	1.74	[0.59; 5.14]	0.3%	2.7%
May - Jul 2020; Rennes, France (Paris)	13	173	43	1140	2.07	[1.09; 3.94]	0.7%	4.3%
Mar - Jul 2020; Hamburg, Germany (Brehm)	6	69	5	226	4.21	[1.24; 14.25]	0.1%	2.4%
Mar - Apr 2020; St. Gallen, Switzerland (Kohler)	7	234	3	565	5.78	[1.48; 22.54]	0.1%	2.1%
Dec 2019 - Jan 2020; Wuhan, China (Ran)	24	49	4	23	4.56	[1.35; 15.37]	0.2%	2.4%
**Fixed effects model**		**595**		**2976**	**2.19**	**[1.50; 3.21]**	**2.0%**	**.**
**Random effects model**					**2.29**	**[1.34; 3.89]**	**.**	**17.9%**
Heterogeneity: $I^2 = 34\%$, $p = 0.17$								
Test for effect in subgroup (common effect): $z = 4.05$ ($p < 0.001$)								
Test for effect in subgroup (random effects): $z = 3.04$ ($p = 0.002$)								
**Subgroup = Cross-sectional**								
Sep 2020 - Dec 2021; Kolkata, India (Chandra)	2	45	28	197	0.28	[0.06; 1.22]	0.6%	1.9%
Dec 2020 - Mar 2021; nationwide, UK (Martin)	1634	6182	859	4560	1.55	[1.41; 1.70]	45.9%	6.1%
Apr 2020 - Jan 2021; Adamawa, Nigeria (Stephen)	43	208	10	110	2.61	[1.25; 5.42]	0.7%	3.9%
Aug - Nov 2020; Multicentric, India (Ghosh)	71	288	575	2965	1.36	[1.02; 1.80]	4.8%	5.7%
Jul - Aug 2020; Rhode Island, USA (Akinbami)	88	1490	227	3128	0.80	[0.62; 1.03]	8.7%	5.7%
Jul - Aug 2020; Bukavu, DRC (Mukwege)	3	5	48	119	2.22	[0.36; 13.78]	0.1%	1.4%
Jun - Aug 2020; Northern/Eastern Switzerland (Kahlert)	8	47	59	1275	4.23	[1.89; 9.45]	0.2%	3.6%
May - Aug 2020; Amhara, Ethiopia (Atnafie)	47	238	31	180	1.18	[0.72; 1.95]	1.8%	4.8%
Jul 2020; Boston, USA (Kataria)	0	12	85	1549	0.69	[0.04; 11.67]	0.1%	0.6%
Jun - Jul 2020; New Delhi, India (Gupta)	38	300	193	1473	0.96	[0.66; 1.40]	3.6%	5.3%
Jun 2020; Mumbai, India (Mutha)	13	222	13	130	0.56	[0.25; 1.25]	1.0%	3.7%
May - Jun 2020; Atlanta, USA (Howard-Anderson)	3	74	19	242	0.50	[0.14; 1.73]	0.5%	2.3%
Apr - Jun 2020; Atlanta, USA (Baker)	14	221	370	6320	1.09	[0.63; 1.89]	1.5%	4.6%
Mar - May 2020; NYC, USA (Venugopal)	1	2	90	361	3.01	[0.19; 48.64]	0.0%	0.7%
Apr 2020; Madrid, Spain (Galan)	219	522	374	1354	1.89	[1.53; 2.34]	7.6%	5.9%
Mar - Apr 2020; Alicante, Spain (Algado-Selles)	112	491	40	293	1.87	[1.26; 2.77]	2.4%	5.3%
**Fixed effects model**		**10347**		**24256**	**1.44**	**[1.34; 1.55]**	**79.7%**	**.**
**Random effects model**					**1.30**	**[1.00; 1.69]**	**.**	**61.5%**
Heterogeneity: $I^2 = 75\%$, $p < 0.001$								
Test for effect in subgroup (common effect): $z = 10.10$ ($p < 0.001$)								
Test for effect in subgroup (random effects): $z = 1.94$ ($p = 0.05$)								
**Fixed effects model**		**12297**		**29232**	**1.37**	**[1.28; 1.46]**	**100.0%**	**.**
**Random effects model**					**1.45**	**[1.14; 1.84]**	**.**	**100.0%**

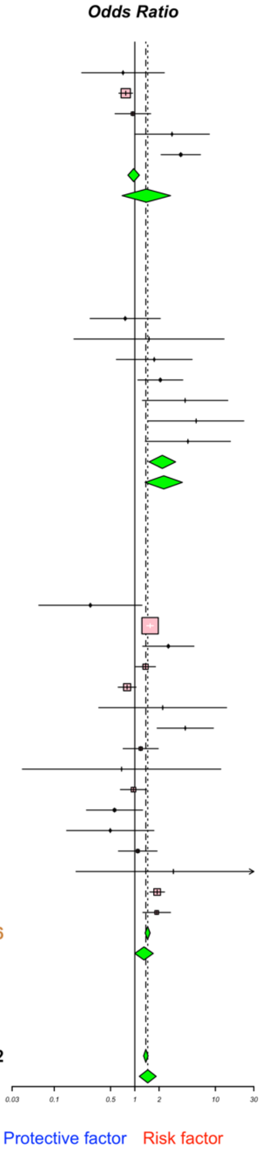

Heterogeneity: $I^2 = 79\%$, $p < 0.001$
Test for overall effect (common effect): $z = 9.60$ ($p < 0.001$)
Test for overall effect (random effects): $z = 3.00$ ($p = 0.003$)
Test for subgroup differences (common effect): $\chi^2_2 = 25.16$, df = 2 ($p < 0.001$)
Test for subgroup differences (random effects): $\chi^2_2 = 3.48$, df = 2 ($p = 0.18$)

Supplementary figure 10: Funnel and Doi plot for meta-analysis involving PPE use

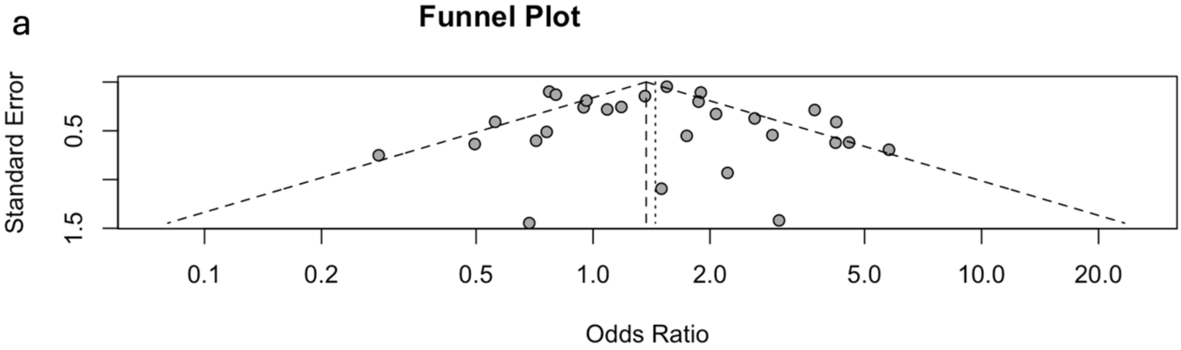

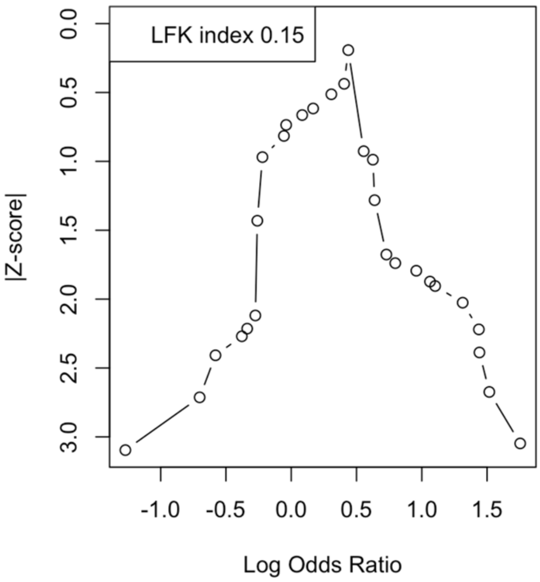

Supplementary figure 10a and 10b visualise funnel and Doi plots respectively. Funnel and Doi plots did not reveal publication bias in meta-analysis determining insufficient PPE use risk factor for SARS-CoV-2 infection in HCWs.

Supplementary figure 11: Funnel and Doi plot for meta-analyses involving infective prevention and control (IPC) training and aerosol-generating procedures

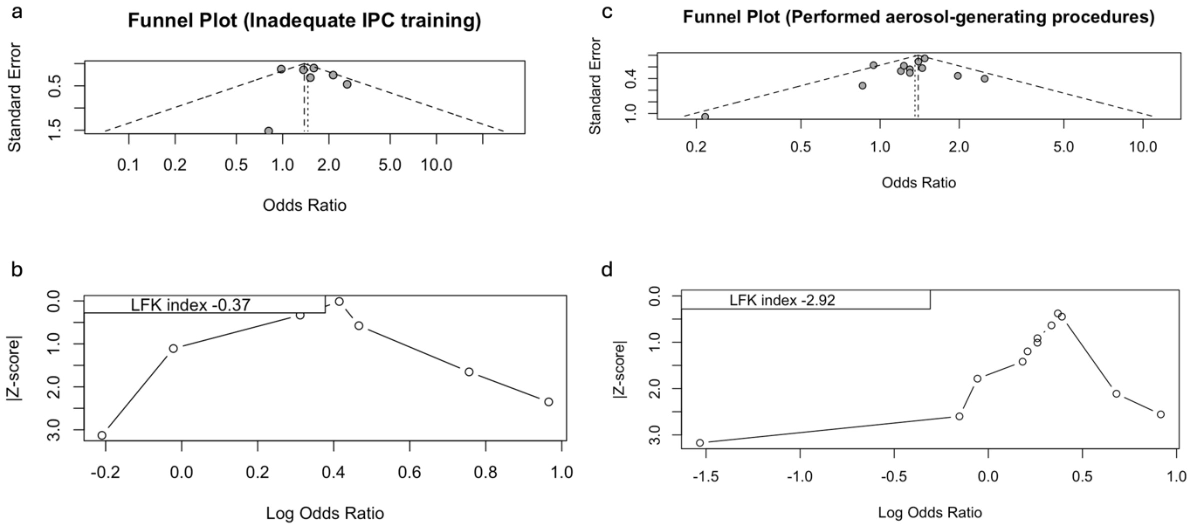

Supplementary figure 11a-c and 11b-d visualise funnel and Doi plots respectively. Funnel plots are used to assess publication bias, while Doi plots visualise and quantify the asymmetry of study effects in meta-analyses examining inadequate IPC training (supplementary figure 11a-b) and performed aerosol-generating procedures (supplementary figure 11c-d) risk factors for SARS-CoV-2 infection in HCWs.

Supplementary figure 12: Funnel and Doi plot for meta-analyses involving working as a cleaner, decontamination, hand hygiene and quarantine

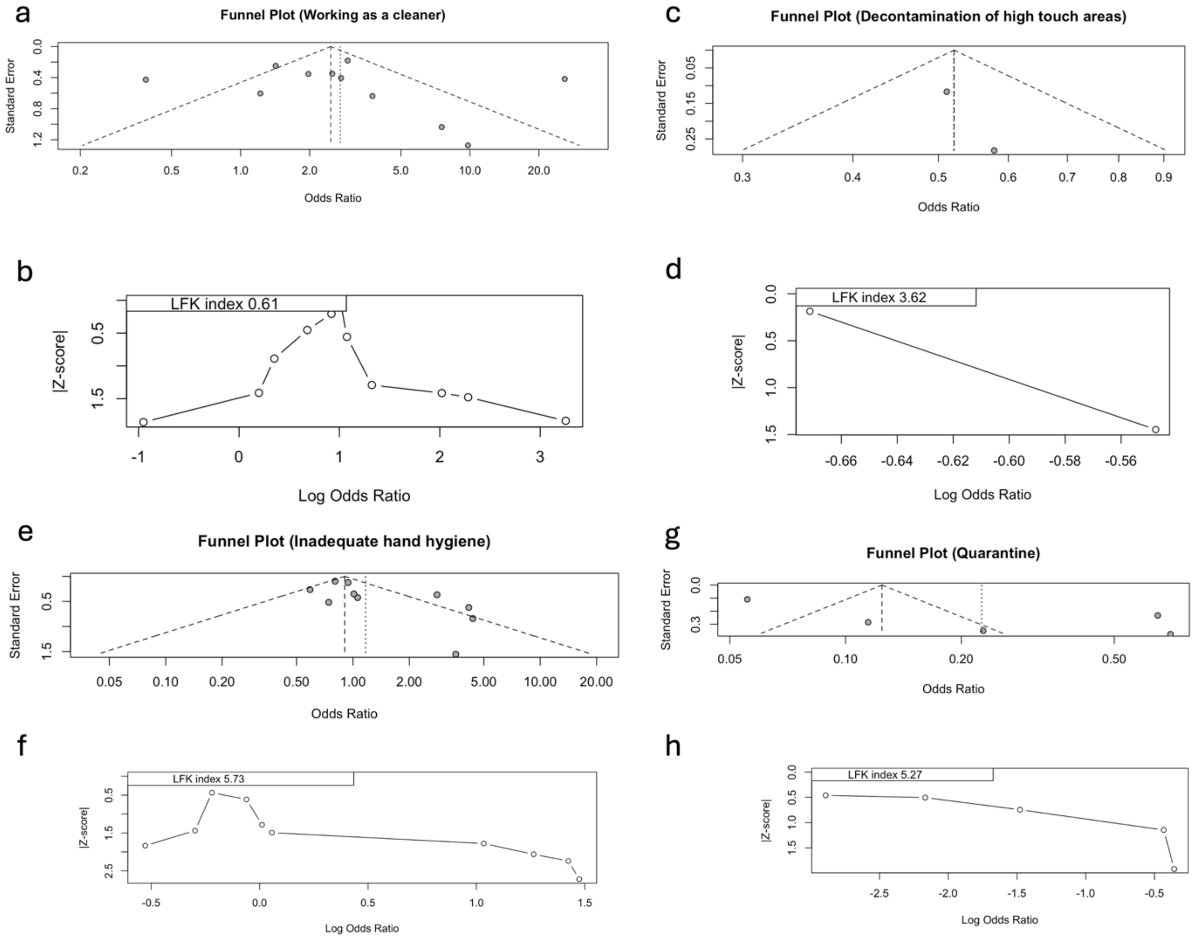

Supplementary figure 12a-c, e-g and 12b-d, f-h visualise funnel and Doi plots respectively. Funnel plots are used to assess publication bias, while Doi plots visualise and quantify the asymmetry of study effects in meta-analyses examining working as a cleaner (supplementary figure 12a-b), frequent decontamination of high touch areas (supplementary figure 12c-d), inadequate hand hygiene (supplementary figure 12e-f) and history of quarantine (supplementary figure 12g-h) risk factors for SARS-CoV-2 infection in HCWs.

Supplementary figure 13: Funnel and Doi plots for meta-analysis involving household exposure to SARS-CoV-2

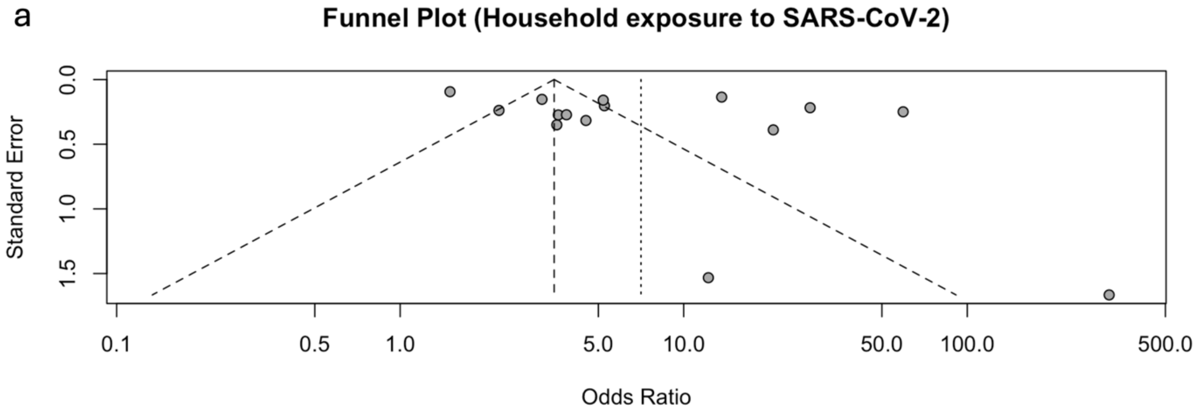

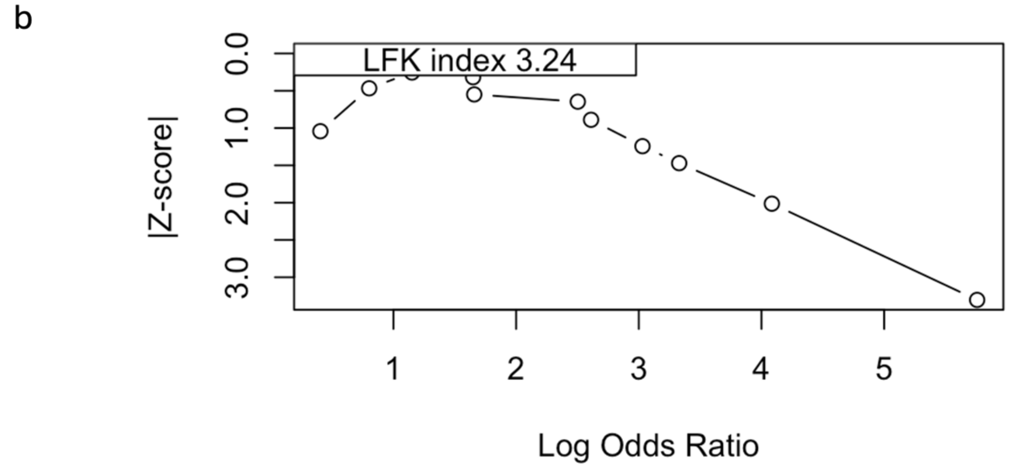

Supplementary figure 13a and 13b visualise funnel and Doi plots respectively. Funnel plots are used to assess publication bias, while Doi plots visualise and quantify the asymmetry of study effects in meta-analyses examining household exposure risk factors for SARS-CoV-2 infection in HCWs.

Supplementary Table 1: Study inclusion and exclusion criteria

**Criteria**	**Include**	**Exclude**
**Study Characteristics**	• Randomised controlled trials (RCTs), observational studies, cohort studies, case-control studies, letters<br>• Published from 1 December 2019 to 5 February 2024	Review, systematic review, meta-analysis
**Population**	• Healthcare workers<br>• Any gender	Heterogenous sample with mixed healthcare and non-healthcare workers
**Exposure**	• Occupational exposure to SARS-CoV-2<br>• Household exposure to SARS-CoV-2<br>• Use of masking while managing individuals with COVID-19<br>• Infection prevention and control training while managing individuals with COVID-19<br>• Hand hygiene while managing individuals with COVID-19<br>• Environmental/hospital infection control	Not applicable
**Control**	Unexposed	Not applicable
**Outcome**	SARS-CoV-2 infection	Studies not reporting quantitative measures. i.e., the number of HCWs who were infected or uninfected with SARS-CoV-2 based on exposure

Supplementary Tables 2-4: Search strategies and concept groups for MEDLINE (total studies, n = 97), Embase (n = 150), and Google Scholar (n = 251) databases

Supplementary Table 2: Search strategy for Ovid MEDLINE(R)

**Search number (no.) 1: SARS-CoV-2 infection rate and exposure to SARS-CoV-2 concept group**		
1	(((exp Coronavirus/ or exp Coronavirus Infections/ or (coronavirus* or corona virus* or ncov* or covid* or sars-cov* or sarscov* or Sars-coronavirus* or Severe Acute Respiratory Syndrome Coronavirus*).mp.) and ("201912" or 2020* or 2021* or 2022* 2023*).dt,ez,da.) not (OC43 or NL63 or 229E or HKU1 or SARS or SARS-CoV or MERS or MERS-CoV or Middle East respiratory syndrome or camel* or dromedar* or equine or coronary or coronal or covidence* or covidien or influenza virus or HIV or bovine or calves or TGEV or feline or porcine or BCoV or PED or PEDV or PDCoV or FIPV or FCoV or SADS-CoV or canine or CCov or zoonotic or avian influenza or H1N1 or H5N1 or H5N6 or IBV or murine corona*).mp.) or (Covid-19/ or covid.mp. or covid19.mp. or 2019-ncov.mp. or ncov19.mp. or ncov-19.mp. or 2019-novel CoV.mp. or sars-cov2.mp. or sars-cov-2.mp. or sarscov2.mp. or sarscov-2.mp. or Sars-coronavirus2.mp. or Sars-coronavirus-2.mp. or SARS-like coronavirus*.mp. or coronavirus-19.mp. or Deltacron.mp. or Omnicron.mp. or ((novel or new or nouveau) adj2 (CoV or nCoV or covid or coronavirus* or corona virus or Pandemi*2)).mp. or ((subvariant* or variant*) adj2 (India* or "South Africa*" or UK or English or Brazil* or alpha or beta or delta or gamma or kappa or lambda or mu or "AY.X" or "BA.1" or "BA.2" or "BA.3" or "BA.4" or "BA.5" or "P.1" or "C.37")).mp. or ("B.1.1.7" or "B.1.351" or "B.1.617.1" or "B.1.617.2" or "B.1.1.529*" or "B.1.61.7*" or "21L/BA.2" or "21K/BA.1" or "XBB.1.5 ").mp.)	387795
2	infection rate.mp.	18399
3	1 and 2	1302
4	(risk adj2 (assessment* or factor or factors)).mp.	1635396
5	protective factors.mp.	20583
6	4 or 5	1644047
7	3 and 6	139
8	limit 7 to (english language and full text and yr="2020 -Current")	5
**Search no. 2: SARS-CoV-2 infection rate and household exposure to SARS-CoV-2 concept group**		
1	COVID-19/pc, tm, vi [Prevention & Control, Transmission, Virology]	41393
2	(risk adj2 (assessment* or factor or factors)).mp.	1635396
3	((((exp Coronavirus/ or exp Coronavirus Infections/ or (coronavirus* or corona virus* or ncov* or covid* or sars-cov* or sarscov* or Sars-coronavirus* or Severe Acute Respiratory Syndrome Coronavirus*).mp.) and ("201912" or 2020* or 2021* or 2022* 2023*).dt,ez,da.) not (OC43 or NL63 or 229E or HKU1 or SARS or SARS-CoV or MERS or MERS-CoV or Middle East respiratory syndrome or camel* or dromedar* or equine or coronary or coronal or covidence* or covidien or influenza virus or HIV or bovine or calves or TGEV or feline or porcine or BCoV or PED or PEDV or PDCoV or FIPV or FCoV or SADS-CoV or canine or CCov or zoonotic or avian influenza or H1N1 or H5N1 or H5N6 or IBV or murine corona*).mp.) or (Covid-19/ or covid.mp. or covid19.mp. or 2019-ncov.mp. or ncov19.mp. or ncov-19.mp. or 2019-novel CoV.mp. or sars-cov2.mp. or sars-cov-2.mp. or sarscov2.mp. or sarscov-2.mp. or Sars-coronavirus2.mp. or Sars-coronavirus-2.mp. or SARS-like coronavirus*.mp. or coronavirus-19.mp. or Deltacron.mp. or Omnicron.mp. or ((novel or new or nouveau) adj2 (CoV or nCoV or covid or coronavirus* or corona virus or Pandemi*2)).mp. or ((subvariant* or variant*) adj2 (India* or "South Africa*" or UK or English or Brazil* or alpha or beta or delta or gamma or kappa or lambda or mu or "AY.X" or "BA.1" or "BA.2" or "BA.3" or "BA.4" or "BA.5" or "P.1" or "C.37")).mp. or ("B.1.1.7" or "B.1.351" or "B.1.617.1" or "B.1.617.2" or "B.1.1.529*" or "B.1.61.7*" or "21L/BA.2" or "21K/BA.1" or "XBB.1.5 ").mp.)) adj3 exposure.mp.	5004
4	2 and 3	682
5	household*.mp.	115366

6	4 and 5	62
7	1 and 6	12
**Search no. 3: SARS-CoV-2 infection rate and PPE use concept group**		
1	mask*.mp.	110079
2	N95.mp.	3161
3	PPE.mp.	7601
4	personal protective equipment.mp.	11388
5	face shield*.mp.	605
6	1 or 2 or 3 or 4 or 5	124812
7	((exp Coronavirus/ or exp Coronavirus Infections/ or (coronavirus* or corona virus* or ncov* or covid* or sars-cov* or sarscov* or Sars-coronavirus* or Severe Acute Respiratory Syndrome Coronavirus* or D614G).mp.) not (OC43 or NL63 or 229E or HKU1 or SARS or SARS-CoV or MERS or MERS-CoV or Middle East respiratory syndrome or camel* or dromedar* or equine or coronary or coronal or covidence* or covidien or influenza virus or HIV or bovine or calves or TGEV or feline or porcine or BCoV or PED or PEDV or PDCoV or FIPV or FCoV or SADS-CoV or canine or CCov or zoonotic or avian influenza or H1N1 or H5N1 or H5N6 or IBV or murine corona*).mp.) or coronavirus disease 2019/ or ((exp pneumonia/ or (pneumonia or covid* or coronavirus* or corona virus* or ncov* or 2019-ncov or sars*).mp.) and Wuhan.mp.) or ("coronavirus disease 2019" or 2019-ncov or ncov19 or ncov-19 or 2019-novel CoV or severe acute respiratory syndrome coronavirus 2 or sars-cov2 or sars-cov-2 or sarscov2 or sarscov-2 or Sars-coronavirus2 or Sars-coronavirus-2 or SARS-like coronavirus* or coronavirus-19 or covid19 or covid-19 or "covid 2019" or "B.1.1.7" or "B.1.351" or "B.1.617.1" or "B.1.617.2" or omicron or Deltacron).mp. or ((subvariant* or variant*) adj2 (India* or "South Africa*" or UK or English or Brazil* or alpha or beta or delta or gamma or kappa or lambda or mu or "AY.X" or "BA.1" or "BA.2" or "BA.3" or "BA.4" or "BA.5" or "P.1" or "C.37")).mp. or ("B.1.1.7" or "B.1.351" or "B.1.617.1" or "B.1.617.2" or "B.1.1.529*" or "B.1.61.7*" or "21L/BA.2" or "21K/BA.1" or "XBB.1.5 ").mp. or ((novel or new or nouveau) adj2 (CoV or nCoV or coronavirus* or corona virus)).mp.	393697
8	infection rate.mp.	18399
9	6 and 7 and 8	121
10	(risk adj2 (assessment* or factor or factors)).mp.	1635396
11	protective factors.mp.	20583
12	10 or 11	1644047
13	9 and 12	28
**Search no. 4: SARS-CoV-2 infection rate and risk exposure concept group**		
1	infection rate.mp.	18399
2	(risk adj2 (assessment* or factor or factors)).mp.	1635396
3	((((exp Coronavirus/ or exp Coronavirus Infections/ or (coronavirus* or corona virus* or ncov* or covid* or sars-cov* or sarscov* or Sars-coronavirus* or Severe Acute Respiratory Syndrome Coronavirus*).mp.) and ("201912" or 2020* or 2021* or 2022* 2023*).dt,ez,da.) not (OC43 or NL63 or 229E or HKU1 or SARS or SARS-CoV or MERS or MERS-CoV or Middle East respiratory syndrome or camel* or dromedar* or equine or coronary or coronal or covidence* or covidien or influenza virus or HIV or bovine or calves or TGEV or feline or porcine or BCoV or PED or PEDV or PDCoV or FIPV or FCoV or SADS-CoV or canine or CCov or zoonotic or avian influenza or H1N1 or H5N1 or H5N6 or IBV or murine corona*).mp.) or (Covid-19/ or covid.mp. or covid19.mp. or 2019-ncov.mp. or ncov19.mp. or ncov-19.mp. or 2019-novel CoV.mp. or sars-cov2.mp. or sars-cov-2.mp. or sarscov2.mp. or sarscov-2.mp. or Sars-coronavirus2.mp. or Sars-coronavirus-2.mp. or SARS-like coronavirus*.mp. or coronavirus-19.mp. or Deltacron.mp. or Omnicron.mp. or ((novel or new or nouveau) adj2 (CoV or nCoV or covid or coronavirus* or corona virus or Pandemi*2)).mp. or ((subvariant* or variant*) adj2 (India* or "South Africa*" or UK or English or Brazil* or alpha or beta or delta or gamma or kappa or lambda or mu or "AY.X" or "BA.1" or "BA.2" or "BA.3" or "BA.4" or "BA.5" or	5004

	"P.1" or "C.37")).mp. or ("B.1.1.7" or "B.1.351" or "B.1.617.1" or "B.1.617.2" or "B.1.1.529*" or "B.1.61.7*" or "21L/BA.2" or "21K/BA.1" or "XBB.1.5 ").mp.)) adj3 exposure.mp.	
4	1 and 2 and 3	7
**Search no. 5: SARS-CoV-2 infection rate and IPC training concept group**		
1	infection rate.mp.	18399
2	(risk adj2 (assessment* or factor or factors)).mp.	1635396
3	((((exp Coronavirus/ or exp Coronavirus Infections/ or (coronavirus* or corona virus* or ncov* or covid* or sars-cov* or sarscov* or Sars-coronavirus* or Severe Acute Respiratory Syndrome Coronavirus*).mp.) and ("201912" or 2020* or 2021* or 2022* 2023*).dt,ez,da.) not (OC43 or NL63 or 229E or HKU1 or SARS or SARS-CoV or MERS or MERS-CoV or Middle East respiratory syndrome or camel* or dromedar* or equine or coronary or coronal or covidence* or covidien or influenza virus or HIV or bovine or calves or TGEV or feline or porcine or BCoV or PED or PEDV or PDCoV or FIPV or FCoV or SADS-CoV or canine or CCov or zoonotic or avian influenza or H1N1 or H5N1 or H5N6 or IBV or murine corona*).mp.) or (Covid-19/ or covid.mp. or covid19.mp. or 2019-ncov.mp. or ncov19.mp. or ncov-19.mp. or 2019-novel CoV.mp. or sars-cov2.mp. or sars-cov-2.mp. or sarscov2.mp. or sarscov-2.mp. or Sars-coronavirus2.mp. or Sars-coronavirus-2.mp. or SARS-like coronavirus*.mp. or coronavirus-19.mp. or Deltacron.mp. or Omnicron.mp. or ((novel or new or nouveau) adj2 (CoV or nCoV or covid or coronavirus* or corona virus or Pandemi*2)).mp. or ((subvariant* or variant*) adj2 (India* or "South Africa*" or UK or English or Brazil* or alpha or beta or delta or gamma or kappa or lambda or mu or "AY.X" or "BA.1" or "BA.2" or "BA.3" or "BA.4" or "BA.5" or "P.1" or "C.37")).mp. or ("B.1.1.7" or "B.1.351" or "B.1.617.1" or "B.1.617.2" or "B.1.1.529*" or "B.1.61.7*" or "21L/BA.2" or "21K/BA.1" or "XBB.1.5 ").mp.)) adj3 exposure.mp.	5004
4	(train* or prevent* or control or educat*).mp.	7076463
5	1 and 2 and 3 and 4	4
**Search no. 6: SARS-CoV-2 infection rate and hand hygiene concept group**		
1	(risk adj2 (assessment* or factor or factors)).mp.	1635396
2	((((exp Coronavirus/ or exp Coronavirus Infections/ or (coronavirus* or corona virus* or ncov* or covid* or sars-cov* or sarscov* or Sars-coronavirus* or Severe Acute Respiratory Syndrome Coronavirus*).mp.) and ("201912" or 2020* or 2021* or 2022* 2023*).dt,ez,da.) not (OC43 or NL63 or 229E or HKU1 or SARS or SARS-CoV or MERS or MERS-CoV or Middle East respiratory syndrome or camel* or dromedar* or equine or coronary or coronal or covidence* or covidien or influenza virus or HIV or bovine or calves or TGEV or feline or porcine or BCoV or PED or PEDV or PDCoV or FIPV or FCoV or SADS-CoV or canine or CCov or zoonotic or avian influenza or H1N1 or H5N1 or H5N6 or IBV or murine corona*).mp.) or (Covid-19/ or covid.mp. or covid19.mp. or 2019-ncov.mp. or ncov19.mp. or ncov-19.mp. or 2019-novel CoV.mp. or sars-cov2.mp. or sars-cov-2.mp. or sarscov2.mp. or sarscov-2.mp. or Sars-coronavirus2.mp. or Sars-coronavirus-2.mp. or SARS-like coronavirus*.mp. or coronavirus-19.mp. or Deltacron.mp. or Omnicron.mp. or ((novel or new or nouveau) adj2 (CoV or nCoV or covid or coronavirus* or corona virus or Pandemi*2)).mp. or ((subvariant* or variant*) adj2 (India* or "South Africa*" or UK or English or Brazil* or alpha or beta or delta or gamma or kappa or lambda or mu or "AY.X" or "BA.1" or "BA.2" or "BA.3" or "BA.4" or "BA.5" or "P.1" or "C.37")).mp. or ("B.1.1.7" or "B.1.351" or "B.1.617.1" or "B.1.617.2" or "B.1.1.529*" or "B.1.61.7*" or "21L/BA.2" or "21K/BA.1" or "XBB.1.5 ").mp.)) adj3 exposure.mp.	5004
3	hand hygiene.mp.	6795
4	1 and 2 and 3	15
**Search no. 7: SARS-CoV-2 infection rate and hospital or environment infection control concept group**		
1	(((exp Coronavirus/ or exp Coronavirus Infections/ or (coronavirus* or corona virus* or ncov* or covid* or sars-cov* or sarscov* or Sars-coronavirus* or Severe Acute	387795

	Respiratory Syndrome Coronavirus*).mp.) and ("201912" or 2020* or 2021* or 2022* 2023*).dt,ez,da.) not (OC43 or NL63 or 229E or HKU1 or SARS or SARS-CoV or MERS or MERS-CoV or Middle East respiratory syndrome or camel* or dromedar* or equine or coronary or coronal or covidence* or covidien or influenza virus or HIV or bovine or calves or TGEV or feline or porcine or BCoV or PED or PEDV or PDCoV or FIPV or FCoV or SADS-CoV or canine or CCov or zoonotic or avian influenza or H1N1 or H5N1 or H5N6 or IBV or murine corona*).mp.) or (Covid-19/ or covid.mp. or covid19.mp. or 2019-ncov.mp. or ncov19.mp. or ncov-19.mp. or 2019-novel CoV.mp. or sars-cov2.mp. or sars-cov-2.mp. or sarscov2.mp. or sarscov-2.mp. or Sars-coronavirus2.mp. or Sars-coronavirus-2.mp. or SARS-like coronavirus*.mp. or coronavirus-19.mp. or Deltacron.mp. or Omnicron.mp. or ((novel or new or nouveau) adj2 (CoV or nCoV or covid or coronavirus* or corona virus or Pandemi*2)).mp. or ((subvariant* or variant*) adj2 (India* or "South Africa*" or UK or English or Brazil* or alpha or beta or delta or gamma or kappa or lambda or mu or "AY.X" or "BA.1" or "BA.2" or "BA.3" or "BA.4" or "BA.5" or "P.1" or "C.37")).mp. or ("B.1.1.7" or "B.1.351" or "B.1.617.1" or "B.1.617.2" or "B.1.1.529*" or "B.1.61.7*" or "21L/BA.2" or "21K/BA.1" or "XBB.1.5 ").mp.)	
2	((environment* or hospital*) adj2 infection control).mp.	1480
3	1 and 2	108
4	(risk adj2 (assessment* or factor or factors)).mp.	1635396
5	3 and 4	13
**Search no. 8: SARS-CoV-2 infection rate and risk assessment concept group**		
1	(((exp Coronavirus/ or exp Coronavirus Infections/ or (coronavirus* or corona virus* or ncov* or covid* or sars-cov* or sarscov* or Sars-coronavirus* or Severe Acute Respiratory Syndrome Coronavirus*).mp.) and ("201912" or 2020* or 2021* or 2022* 2023*).dt,ez,da.) not (OC43 or NL63 or 229E or HKU1 or SARS or SARS-CoV or MERS or MERS-CoV or Middle East respiratory syndrome or camel* or dromedar* or equine or coronary or coronal or covidence* or covidien or influenza virus or HIV or bovine or calves or TGEV or feline or porcine or BCoV or PED or PEDV or PDCoV or FIPV or FCoV or SADS-CoV or canine or CCov or zoonotic or avian influenza or H1N1 or H5N1 or H5N6 or IBV or murine corona*).mp.) or (Covid-19/ or covid.mp. or covid19.mp. or 2019-ncov.mp. or ncov19.mp. or ncov-19.mp. or 2019-novel CoV.mp. or sars-cov2.mp. or sars-cov-2.mp. or sarscov2.mp. or sarscov-2.mp. or Sars-coronavirus2.mp. or Sars-coronavirus-2.mp. or SARS-like coronavirus*.mp. or coronavirus-19.mp. or Deltacron.mp. or Omnicron.mp. or ((novel or new or nouveau) adj2 (CoV or nCoV or covid or coronavirus* or corona virus or Pandemi*2)).mp. or ((subvariant* or variant*) adj2 (India* or "South Africa*" or UK or English or Brazil* or alpha or beta or delta or gamma or kappa or lambda or mu or "AY.X" or "BA.1" or "BA.2" or "BA.3" or "BA.4" or "BA.5" or "P.1" or "C.37")).mp. or ("B.1.1.7" or "B.1.351" or "B.1.617.1" or "B.1.617.2" or "B.1.1.529*" or "B.1.61.7*" or "21L/BA.2" or "21K/BA.1" or "XBB.1.5 ").mp.)	387795
2	infection rate.mp.	18399
3	1 and 2	1302
4	(risk adj2 (assessment* or factor or factors)).mp.	1635396
5	protective factors.mp.	20583
6	4 or 5	1644047
7	3 and 6	139
8	limit 7 to (english language and full text and yr="2020 -Current")	13
**Grand total of 8 search strategies**		97

Supplementary Table 3: Search strategy for Embase

**Search no. 1: SARS-CoV-2 infection rate and occupational/household exposure concept group**		
1	((exp Coronavirus/ or exp Coronavirus Infections/ or (coronavirus* or corona virus* or ncov* or covid* or sars-cov* or sarscov* or Sars-coronavirus* or Severe Acute Respiratory Syndrome Coronavirus* or D614G).mp.) not (OC43 or NL63 or 229E or HKU1 or SARS or SARS-CoV or MERS or MERS-CoV or Middle East respiratory syndrome or camel* or dromedar* or equine or coronary or coronal or covidence* or covidien or influenza virus or HIV or bovine or calves or TGEV or feline or porcine or BCoV or PED or PEDV or PDCoV or FIPV or FCoV or SADS-CoV or canine or CCov or zoonotic or avian influenza or H1N1 or H5N1 or H5N6 or IBV or murine corona*).mp.) or coronavirus disease 2019/ or ((exp pneumonia/ or (pneumonia or covid* or coronavirus* or corona virus* or ncov* or 2019-ncov or sars*).mp.) and Wuhan.mp.) or ("coronavirus disease 2019" or 2019-ncov or ncov19 or ncov-19 or 2019-novel CoV or severe acute respiratory syndrome coronavirus 2 or sars-cov2 or sars-cov-2 or sarscov2 or sarscov-2 or Sars-coronavirus2 or Sars-coronavirus-2 or SARS-like coronavirus* or coronavirus-19 or covid19 or covid-19 or "covid 2019" or "B.1.1.7" or "B.1.351" or "B.1.617.1" or "B.1.617.2" or omicron or Deltacron).mp. or ((subvariant* or variant*) adj2 (India* or "South Africa*" or UK or English or Brazil* or alpha or beta or delta or gamma or kappa or lambda or mu or "AY.X" or "BA.1" or "BA.2" or "BA.3" or "BA.4" or "BA.5" or "P.1" or "C.37")).mp. or ("B.1.1.7" or "B.1.351" or "B.1.617.1" or "B.1.617.2" or "B.1.1.529*" or "B.1.61.7*" or "21L/BA.2" or "21K/BA.1" or "XBB.1.5 ").mp. or ((novel or new or nouveau) adj2 (CoV or nCoV or coronavirus* or corona virus)).mp.	464904
2	infection rate.mp.	54935
3	1 and 2	4645
4	(risk adj2 (assessment* or factor or factors)).mp.	2328739
5	protective factors.mp.	19667
6	4 or 5	2337342
7	household*.mp.	146959
8	(occupational or hospital* or health setting).mp.	3743897
9	7 and 8	21358
10	7 and 9	21358
11	3 and 6	852
12	10 and 11	13
**Search no. 2: SARS-CoV-2 infection rate and PPE use concept group**		
1	((exp Coronavirus/ or exp Coronavirus Infections/ or (coronavirus* or corona virus* or ncov* or covid* or sars-cov* or sarscov* or Sars-coronavirus* or Severe Acute Respiratory Syndrome Coronavirus* or D614G).mp.) not (OC43 or NL63 or 229E or HKU1 or SARS or SARS-CoV or MERS or MERS-CoV or Middle East respiratory syndrome or camel* or dromedar* or equine or coronary or coronal or covidence* or covidien or influenza virus or HIV or bovine or calves or TGEV or feline or porcine or BCoV or PED or PEDV or PDCoV or FIPV or FCoV or SADS-CoV or canine or CCov or zoonotic or avian influenza or H1N1 or H5N1 or H5N6 or IBV or murine corona*).mp.) or coronavirus disease 2019/ or ((exp pneumonia/ or (pneumonia or covid* or coronavirus* or corona virus* or ncov* or 2019-ncov or sars*).mp.) and Wuhan.mp.) or ("coronavirus disease 2019" or 2019-ncov or ncov19 or ncov-19 or 2019-novel CoV or severe acute respiratory syndrome coronavirus 2 or sars-cov2 or sars-cov-2 or sarscov2 or sarscov-2 or Sars-coronavirus2 or Sars-coronavirus-2 or SARS-like coronavirus* or coronavirus-19 or covid19 or covid-19 or "covid 2019" or "B.1.1.7" or "B.1.351" or "B.1.617.1" or "B.1.617.2" or omicron or Deltacron).mp. or ((subvariant* or variant*) adj2 (India* or "South Africa*" or UK or English or Brazil* or alpha or beta or delta or gamma or kappa or lambda or mu or "AY.X" or "BA.1" or "BA.2" or "BA.3" or "BA.4" or "BA.5" or "P.1" or "C.37")).mp. or ("B.1.1.7" or "B.1.351" or "B.1.617.1" or "B.1.617.2" or "B.1.1.529*" or "B.1.61.7*" or "21L/BA.2" or "21K/BA.1" or "XBB.1.5 ").mp. or ((novel or new or nouveau) adj2 (CoV or nCoV or coronavirus* or corona virus)).mp.	464904

2	infection rate.mp.	54935
3	1 and 2	4645
4	(risk adj2 (assessment* or factor or factors)).mp.	2328739
5	protective factors.mp.	19667
6	4 or 5	2337342
7	mask*.mp.	165464
8	N95.mp.	5528
9	PPE.mp.	9731
10	personal protective equipment.mp.	10932
11	face shield*.mp.	1532
12	7 or 8 or 9 or 10 or 11	183273
13	3 and 6	852
14	12 and 13	81
**Search no. 3: SARS-CoV-2 infection rate and IPC training concept group**		
1	((exp Coronavirus/ or exp Coronavirus Infections/ or (coronavirus* or corona virus* or ncov* or covid* or sars-cov* or sarscov* or Sars-coronavirus* or Severe Acute Respiratory Syndrome Coronavirus* or D614G).mp.) not (OC43 or NL63 or 229E or HKU1 or SARS or SARS-CoV or MERS or MERS-CoV or Middle East respiratory syndrome or camel* or dromedar* or equine or coronary or coronal or covidence* or covidien or influenza virus or HIV or bovine or calves or TGEV or feline or porcine or BCoV or PED or PEDV or PDCoV or FIPV or FCoV or SADS-CoV or canine or CCov or zoonotic or avian influenza or H1N1 or H5N1 or H5N6 or IBV or murine corona*).mp.) or coronavirus disease 2019/ or ((exp pneumonia/ or (pneumonia or covid* or coronavirus* or corona virus* or ncov* or 2019-ncov or sars*).mp.) and Wuhan.mp.) or ("coronavirus disease 2019" or 2019-ncov or ncov19 or ncov-19 or 2019-novel CoV or severe acute respiratory syndrome coronavirus 2 or sars-cov2 or sars-cov-2 or sarscov2 or sarscov-2 or Sars-coronavirus2 or Sars-coronavirus-2 or SARS-like coronavirus* or coronavirus-19 or covid19 or covid-19 or "covid 2019" or "B.1.1.7" or "B.1.351" or "B.1.617.1" or "B.1.617.2" or omicron or Deltacron).mp. or ((subvariant* or variant*) adj2 (India* or "South Africa*" or UK or English or Brazil* or alpha or beta or delta or gamma or kappa or lambda or mu or "AY.X" or "BA.1" or "BA.2" or "BA.3" or "BA.4" or "BA.5" or "P.1" or "C.37")).mp. or ("B.1.1.7" or "B.1.351" or "B.1.617.1" or "B.1.617.2" or "B.1.1.529*" or "B.1.61.7*" or "21L/BA.2" or "21K/BA.1" or "XBB.1.5 ").mp. or ((novel or new or nouveau) adj2 (CoV or nCoV or coronavirus* or corona virus)).mp.	464904
2	infection rate.mp.	54935
3	1 and 2	4645
4	(risk adj2 (assessment* or factor or factors)).mp.	2328739
5	protective factors.mp.	19667
6	4 or 5	2337342
7	(train* or prevent* or control or educat*).mp.	9501144
8	exposure.mp.	1582416
9	3 and 6	852
10	7 and 9	412
11	8 and 10	50
**Search no. 4: SARS-CoV-2 infection rate and hand hygiene concept group**		
1	((exp Coronavirus/ or exp Coronavirus Infections/ or (coronavirus* or corona virus* or ncov* or covid* or sars-cov* or sarscov* or Sars-coronavirus* or Severe Acute Respiratory Syndrome Coronavirus* or D614G).mp.) not (OC43 or NL63 or 229E or HKU1 or SARS or SARS-CoV or MERS or MERS-CoV or Middle East respiratory syndrome or camel* or dromedar* or equine or coronary or coronal or covidence* or covidien or influenza virus or HIV or bovine or calves or TGEV or feline or porcine or BCoV or PED or PEDV or PDCoV or FIPV or FCoV or SADS-CoV or canine or CCov or zoonotic or avian influenza or H1N1 or H5N1 or H5N6 or IBV or murine corona*).mp.) or coronavirus disease 2019/ or ((exp pneumonia/ or (pneumonia or covid* or coronavirus* or corona virus* or ncov* or 2019-ncov or sars*).mp.) and	464904

	Wuhan.mp.) or ("coronavirus disease 2019" or 2019-ncov or ncov19 or ncov-19 or 2019-novel CoV or severe acute respiratory syndrome coronavirus 2 or sars-cov2 or sars-cov-2 or sarscov2 or sarscov-2 or Sars-coronavirus2 or Sars-coronavirus-2 or SARS-like coronavirus* or coronavirus-19 or covid19 or covid-19 or "covid 2019" or "B.1.1.7" or "B.1.351" or "B.1.617.1" or "B.1.617.2" or omicron or Deltacron).mp. or ((subvariant* or variant*) adj2 (India* or "South Africa*" or UK or English or Brazil* or alpha or beta or delta or gamma or kappa or lambda or mu or "AY.X" or "BA.1" or "BA.2" or "BA.3" or "BA.4" or "BA.5" or "P.1" or "C.37")).mp. or ("B.1.1.7" or "B.1.351" or "B.1.617.1" or "B.1.617.2" or "B.1.1.529*" or "B.1.61.7*" or "21L/BA.2" or "21K/BA.1" or "XBB.1.5 ").mp. or ((novel or new or nouveau) adj2 (CoV or nCoV or coronavirus* or corona virus)).mp.	
2	infection rate.mp.	54935
3	1 and 2	4645
4	(risk adj2 (assessment* or factor or factors)).mp.	2328739
5	protective factors.mp.	19667
6	4 or 5	2337342
7	hand hygiene.mp.	9767
8	3 and 6	852
9	7 and 8	5
**Search no. 5: SARS-CoV-2 infection rate and hospital infection control concept group**		
1	((exp Coronavirus/ or exp Coronavirus Infections/ or (coronavirus* or corona virus* or ncov* or covid* or sars-cov* or sarscov* or Sars-coronavirus* or Severe Acute Respiratory Syndrome Coronavirus* or D614G).mp.) not (OC43 or NL63 or 229E or HKU1 or SARS or SARS-CoV or MERS or MERS-CoV or Middle East respiratory syndrome or camel* or dromedar* or equine or coronary or coronal or covidence* or covidien or influenza virus or HIV or bovine or calves or TGEV or feline or porcine or BCoV or PED or PEDV or PDCoV or FIPV or FCoV or SADS-CoV or canine or CCov or zoonotic or avian influenza or H1N1 or H5N1 or H5N6 or IBV or murine corona*).mp.) or coronavirus disease 2019/ or ((exp pneumonia/ or (pneumonia or covid* or coronavirus* or corona virus* or ncov* or 2019-ncov or sars*).mp.) and Wuhan.mp.) or ("coronavirus disease 2019" or 2019-ncov or ncov19 or ncov-19 or 2019-novel CoV or severe acute respiratory syndrome coronavirus 2 or sars-cov2 or sars-cov-2 or sarscov2 or sarscov-2 or Sars-coronavirus2 or Sars-coronavirus-2 or SARS-like coronavirus* or coronavirus-19 or covid19 or covid-19 or "covid 2019" or "B.1.1.7" or "B.1.351" or "B.1.617.1" or "B.1.617.2" or omicron or Deltacron).mp. or ((subvariant* or variant*) adj2 (India* or "South Africa*" or UK or English or Brazil* or alpha or beta or delta or gamma or kappa or lambda or mu or "AY.X" or "BA.1" or "BA.2" or "BA.3" or "BA.4" or "BA.5" or "P.1" or "C.37")).mp. or ("B.1.1.7" or "B.1.351" or "B.1.617.1" or "B.1.617.2" or "B.1.1.529*" or "B.1.61.7*" or "21L/BA.2" or "21K/BA.1" or "XBB.1.5 ").mp. or ((novel or new or nouveau) adj2 (CoV or nCoV or coronavirus* or corona virus)).mp.	464904
2	infection rate.mp.	54935
3	1 and 2	4645
4	(risk adj2 (assessment* or factor or factors)).mp.	2328739
5	protective factors.mp.	19667
6	4 or 5	2337342
7	((environment* or hospital*) adj2 infection control).mp.	2117
8	3 and 6	852
9	7 and 8	1
**Grand total of 5 search strategies**		150

Supplementary Table 4: Search strategy for Google Scholar

1	allintitle: (coronavirus OR "2019 nCoV" OR "2019 novel" OR "2019 nCoV" OR "2019 nCoV" OR "COVID 19" OR COVID19 OR "COVID 19" OR "SARS CoV2" OR "SARS CoV 2" OR "severe acute respiratory syndrome Coronavirus 2" OR seroprevalence AND “healthcare worker” OR “risk factors” OR “public health intervention”)	13,600
2	Limiting search to the first 251 results	251

Supplementary Table 5: Characterising main findings of the included 63 articles.

Study no.	Author	Study design	Data collection (time; location)	Key findings in healthcare workers (HCWs)*
1.	Chandra[1]	Cross-sectional	Sep 2020 - Dec 2021; Kolkata, India	• Out of the 242 HCWs, 30 (12 %) tested positive for SARS-CoV-2 antibodies.<br>• Despite active IPC policies, high-risk exposure and the presence of comorbidities were significant risk factors for acquiring the disease.
2.	George[2]	Nested case–control	Dec 2020 - Dec 2021; 7 states, India	• The risk factors associated with SARS-CoV-2 infection were age over 31 years, male gender, IPC training, direct exposure to patient with diabetes mellitus and/or COVID-19.<br>• Emphasised the necessity of having a separate hospital IPC department.
3.	Dube[3]	Cohort	Aug 2020 - May 2021; Atlanta, Georgia, USA	• Job type remained a major factor in the increased risk of infection during the first year of the pandemic.<br>• SARS-CoV-2 infection measured by seroconversion was significantly higher among nursing staff.
4.	Allen[4]	Cross-sectional	Apr 2021; Dublin & Galway, Ireland	• The higher HCW seroprevalence reflects the third wave of the pandemic in Ireland.<br>• Concerted efforts needed to address risk factors related to ethnicity and lower education levels
5.	Martin[5]	Cross-sectional	Dec 2020 - Mar 2021; nationwide, UK	• Important risk factors for SARS-CoV-2 infection in occupational settings include attending to a higher number of SARS-CoV-2 positive patients, working in a nursing or midwifery role, experiencing a lack of access to PPE, and working in an ambulance or hospital inpatient setting.<br>• ICU workers had a lower chance of infection.
6.	Maina[6]	Cross-sectional	Nov 2020 - Feb 2021; Nairobi, Kenya	• Lower prevalence of SARS-CoV-2 in HCWs than in community<br>• No higher risk observed for staff working in clinical areas compared to those working in non-clinical areas.
7.	Żółtowska[7]	Cross-sectional	Jan 2021; Krakow, Poland	• PPE alone is not enough; hand hygiene and regular SARS-CoV-2 testing for healthcare workers are crucial for infection prevention.
8.	Bueno-Hernández[8]	Prospective cohort	Aug 2020 - Jan 2021; Mexico City, Mexico	• A major proportion (58%) of HCWs contracted SARS-CoV-2, with 67% being asymptomatic.

				• Workplace exposure did not significantly increase infection rates.
9.	Doernberg[9]	Prospective cohort	May 2020 - Jan 2021; Bay area, USA	• Despite consistent exposure at work, SARS-CoV-2 incidence was modest.<br>• Community contact was strongly associated with infections, but workplace contact was not unless it involved high-risk exposure.
10.	Stephen[10]	Retrospective cross-sectional	Apr 2020 - Jan 2021; Adamawa, Nigeria	• Clinical staff had a higher burden of SARS-CoV-2 infection than non-clinical staff, and increasing age increased the risk.
11.	Bansal[11]	Prospective cohort	Mar - Dec 2020; Western Norway	• Norwegian HCWs had a high SARS-CoV-2 infection rate of 11% in 2020.<br>• Infection was associated with household and occupational exposure to SARS-CoV-2.
12.	Chea[12]	Case–control	May - Dec 2020; 5 US states	• HCW's close contact with COVID-19 patients, both inside and outside of the workplace, was linked to a higher risk of infection.
13.	Socan[13]	Cross-sectional	Nov - Dec 2020; 2 sites, Slovenia	• High seroprevalence rate in HCWs (20%) in the early phase of the pandemic
14.	Rosińska[14]	Cross-sectional	Dec 2020; 3 regions, Poland	• Despite IPC measures, HCWs continued to have elevated infection risks, primarily due to close contacts with infected patients.
15.	Ghosh[15]	Cross-sectional	Aug - Nov 2020; Multicentric, India	• High SARS-CoV-2 seropositivity rate of 20% was observed in HCWs.<br>• Statistically significant associations were found with location, category of HCW, previous molecular test positivity, and recent symptoms.
16.	Allen[16]	Cross-sectional	Oct 2020; Dublin & Galway, Ireland	• SARS-CoV-2 seroprevalence was six times higher among HCWs than in the community especially after occupational close contact.<br>• Degree of asymptomatic infections emphasizes the need for stronger IPC among HCWs.
17.	Sharma[17]	Cross-sectional	Aug - Sep 2020; New Delhi, India	• The seropositivity rate of SARS-CoV-2 in HCWs was high (12%), likely lower than in the general population.<br>• Medical doctors had the lowest seropositivity rate, while administrative staff had the highest. Male sex and having lived in a containment zone were associated with past SARS-CoV-2 infection.
18.	Brousseau[18]	Cross-sectional	Jul - Sep 2020; Quebec, Canada	• HCWs in Quebec hospitals were at high risk of occupational SARS-CoV-2 infection. Varied seroprevalence among hospitals, ranging 2-34%.
19.	Akinbami[19]	Cross-sectional	Jul - Aug 2020; Rhode Island, USA	• Seropositivity among 9,863 HCWs was moderate (5%), but it varied 4-fold between hospital (3%) and nursing home personnel (13%).
20.	Goenka[20]	Cross- sectional	Jul - Aug 2020; Kolkata, India	• High overall seroprevalence (12%) in HCWs, which was especially high in gastroenterology (12%), oncology (11%), pathology (10%), emergency medicine (8%) and critical care medicine (7%)

21.	Szajek[21]	Cohort	Jun - Aug 2020; South-eastern Switzerland	• Household exposure to SARS-CoV-2 was most important risk factor for seroconversion in HCWs
22.	Kahlert[22]	Cross- sectional	Jun - Aug 2020; Northern/Eastern Switzerland	• Low seroprevalence (3%) among 4,664 HCWs and household exposure to SARS-CoV-2 was most important risk factor for seroconversion in HCWs
23.	Ludewick[23]	Cross- sectional	Jun - Aug 2020; Western Australia	• Very low transmission of SARS-CoV-2 in HCWs. Out of 799, two cases were related to occupational exposure and 2 due to overseas travel
24.	Papasavas[24]	Longitudinal	May - Aug 2020; Connecticut, USA	• Moderate baseline seropositivity (6%), which was higher in African Americans and Hispanics (10-12%).<br>• Only 61% HCWs remained sero-positive after a median of 164 days (range 90-196)
25.	Jacob[25]	Cross-sectional	Apr - Aug 2020; 3 US states	• Low SARS-CoV-2 seropositivity (4%)<br>• Seropositivity was linked to community exposures, not workplace characteristics
26.	Gupta[26]	Prospective, cross-sectional	Jun - Jul 2020; New Delhi, India	• Overall high SARS-CoV-2 seropositivity (13%); especially in administrative staff and HCWs using public transportation (20%)<br>• Almost half (46%) of infected HCWs were asymptomatic
27.	Paris[27]	Cross-sectional sero-prevalence	May - Jul 2020; Rennes, France	• Low SARS-CoV-2 seropositivity (4%)<br>• Risk factors for HCWs contracting SARS-CoV-2 were occupational and household exposure to SARS-CoV-2 and performing aerosol generating procedures.
28.	Dev[28]	Case–control	May - Jul 2020; Delhi, India	• High SARS-CoV-2 infection rate (16%) in HCWs, with ~45% asymptomatic.<br>• Main risk factors for SARS-CoV-2 infectivity were being a cleaner, improper PPE use.
29.	Rodriguez-Lopez[29]	Case–control	Jun - Jul 2020; Cali, Colombia	• High-performance masks or double masking, and adequate use of PPE were protective factors in HCWs. Gender, level of education along with occupational characteristics, were also associated with infection.
30.	Mutha[30]	Cross-sectional	Jun 2020; Mumbai, India	• Moderate SARS-CoV-2 infection rate (8%) in HCWs, with 22% asymptomatic<br>• Symptoms, but not working in designated COVID-19 area, associated with infection.
31.	Nishida[31]	Cross-sectional	Jun 2020; Osaka, Japan	• Very low SARS-CoV-2 infection rate (0.4%) in HCWs, which was comparable to community seroprevalence in Osaka (0.5%).

32.	Howard-Anderson[32]	Cross-sectional	May - Jun 2020; Atlanta, USA	• Moderate SARS-CoV-2 infection rate (6%) in HCWs.<br>• Spending >50% of a typical shift at the bedside and black race were risk factors for seropositivity.
33.	Brant-Zawadzki[33]	Cross-sectional	May - June 2020; California, USA	• Low SARS-CoV-2 infection rate (1%) in HCWs, which was lower than community seroprevalence (4%).<br>• Hospital's timely procurement of PPE, rigorous employee education, patient triage might have helped curtail the viral spread.
34.	Blairon[34]	Cross-sectional	May - Jun 2020; Brussels, Belgium	• High SARS-CoV-2 infection rate (15%) in HCWs.<br>• Higher seroprevalence in nurses and HCWs who worked in COVID-19 units
35.	Wilkins[35]	Cross-sectional	May - Jun 2020; Chicago, USA	• Moderate SARS-CoV-2 infection rate (5%) in HCWs<br>• Higher seroprevalence in nurses and those with longer exposure to SARS-CoV-2.
36.	Bryan[36]	Cross-sectional	Apr - Jun 2020, New York, USA	• High SARS-CoV-2 infection rates (29%) in HCWs.<br>• Seropositivity was associated with black race and household exposure to SARS-CoV-2.
37.	Baker[37]	Cross-sectional	Apr - Jun 2020; Atlanta, USA	• Low SARS-CoV-2 infection rates (4%) in HCWs.<br>• Seropositivity was associated with black race and community exposure to SARS-CoV-2.
38.	Moscola[38]	Cohort	Apr - Jun 2020; NYC, USA	• High SARS-CoV-2 infection rates (14%) in HCWs.<br>• Seropositivity was associated with higher age and non-white ethnicity, and occupational exposure to SARS-CoV-2
39.	Darvishian[39]	Cross-sectional	Apr - Jun 2020; Tehran, Iran	• High SARS-CoV-2 infection rates (17%) in HCWs.<br>• Higher seropositivity in nurses and private hospitals (due to suboptimal PPE use).
40.	Trieu[40]	Prospective cohort study	Mar - Jun 2020; Bergen, Norway	• A moderate seroprevalence (5%) was found, but a higher rate of seropositivity compared to RT-PCR positivity, highlighting the significance of antibody testing.
41.	Eyre[41]	Prospective observational	Mar - Jun 2020; Oxford, UK	• High SARS-CoV-2 infection rates (11%) in HCWs, which was higher than in community (7%)<br>• Higher seropositivity related to occupational and household exposure to SARS-CoV-2.
42.	Venugopal[42]	Cross-sectional	Mar - May 2020; NYC, USA	• High SARS-CoV-2 infection rates (27%) in HCWs

				• Occupational and community exposure was associated with seropositivity.
43.	Celebi[43]	Case-control	Mar - May 2020; Zonguldak, Turkey	• Moderate SARS-CoV-2 infection rates (7%) in HCWs.<br>• Occupational and household exposures were associated with seropositivity.
44.	Rosser[44]	Sero-surveillance	Apr - May 2020; San Francisco, USA	• Very low SARS-CoV-2 infection rates (1%) in HCWs.<br>• Household exposure, ethnicity and working in environmental services, food services, or patient transport were linked to infection.
45.	Rudberg[45]	Cross- sectional	Apr - May 2020; Danderyd, Sweden	• High SARS-CoV-2 infection rates (19%) in HCWs.<br>• Occupational contact and symptoms (anosmia and ageusia) associated with seropositivity.
46.	Iversen[46]	Cohort	Apr 2020; Copenhagen, Denmark	• Low SARS-CoV-2 infection rates (4%) in HCWs.<br>• Frontline HCWs had higher risk of seropositivity.
47.	Piccoli[47]	Cohort	Apr 2020; Ticino, Switzerland	• High SARS-CoV-2 infection rates (10%) in HCWs, which was only slightly higher than in community; suggesting importance of strict IPC policy at work.<br>• Household contact to SARS-CoV-2 represented major risk factor.
48.	Steensels[48]	Screening	Apr 2020; Genk, Belgium	• Household contact to SARS-CoV-2 represented major risk factor, but not occupational contact.<br>• Hospital-wide antibody sero-surveillance could guide IPC policies.
49.	Galan[49]	Cross-sectional	Apr 2020; Madrid, Spain	• Very high SARS-CoV-2 infection rates (32%) in HCWs, which was particularly higher with occupational exposure.
50.	Sotgiu[50]	Cross-sectional	Apr 2020; Milan, Italy	• High seropositivity rates of 14% was observed in HCWs. Study did not explore relationship with other independent variables.
51.	Garcia-Basteiro[51]	Cross-sectional	Mar - Apr 2020; Barcelona, Spain	• High SARS-CoV-2 infection rate of 11% was observed in HCWs.<br>• Serology identified previously undetected cases (~40%, 26/65), although 12 of them reported COVID-19 compatible symptoms.
52.	Kohler[52]	Cohort	Mar - Apr 2020; St. Gallen, Switzerland	• Low seropositivity rates (1%) in HCWs
53.	Algado-Selles[53]	Observational	Mar - Apr 2020; Alicante, Spain	• Low SARS-CoV-2 infection rate of 4% in HCWs.
54.	Fusco[54]	Cross-sectional	Feb - Mar 2020; Naples, Italy	• Low SARS-CoV-2 infection rate of 3% in HCWs.
55.	Ran[55]	Cohort	Dec 2019 - Jan 2020; Wuhan, China	• HCWs at high occupational exposure to SARS-CoV-2 had 2 times higher risk.

56.	Wang[56]	Cohort	Dec 2019 - Jan 2020; Wuhan, China	• HCWs infected with SARS-CoV-2 had poor sleep quality and higher working pressure than uninfected HCWs.
57.	Lartey[57]	Case-control	Nov 2020 - Jan 2021; three regions, Ghana	• Occupational and community exposure to SARS-CoV-2, and presence of comorbidities were risk factors for infection.
58.	Mukwege[58]	Observational	Jul - Aug 2020; Bukavu, DRC	• Very high SARS-CoV-2 infection rates (41%) in HCWs. Only 22% of reported symptoms after infection and none required hospitalisation.
59.	Atnafie[59]	Cross- sectional	May - Aug 2020; Amhara, Ethiopia	• High SARS-CoV-2 infection rates (19%) in HCWs.<br>• Occupational exposure and higher family size were risk factors for developing infection.
60.	Kataria[60]	Cross-sectional	Jul 2020; Boston, USA	• Moderate SARS-CoV-2 seropositivity (5%) in HCWs.<br>• Lack of physical distancing among HCWs was associated with seropositivity.
61.	Brehm[61]	Cohort	Mar - Jul 2020; Hamburg, Germany	• Low SARS-CoV-2 seropositivity (2%) in HCWs. Occupational and community exposure were risk factors for developing COVID-19.
62.	Chatterjee[62]	Case–control	Apr - May 2020; multi-site	• Moderate SARS-CoV-2 infection rate (5%) in HCWs.<br>• Improper use of PPE and performing aerosol generating procedures were associated with SARS-CoV-2 infection.
63.	Lau[63]	Cross-sectional cohort	Nov - Dec 2020; Victoria, Australia	• Low SARS-CoV-2 infection rate (2%) in HCWs, but higher than in community.<br>• RT-PCR test identified majority of infections. Only 3/720 HCWs were positive by serology and had no positive PCR results

* For easy comparison, SARS-CoV-2 infection rates in healthcare workers were arbitrarily categorised as low (<5%), moderate (5-10%) and high (>10%).

References

1 Chandra, S., Ghosh, R. & Rashid, M. K. A cross-sectional anti-SARS coV-2 seroprevalence study among healthcare workers in a tertiary care hospital of eastern India. *Journal of Dr. NTR University of Health Sciences* **11**, 113-117 (2022). https://doi.org/10.4103/jdrntruhs.jdrntruhs_90_21

2 George, L. S. *et al.* Risk factors for SARS-CoV-2 infection among health workers in India: a case control study. *Front Public Health* **11**, 1156782 (2023). https://doi.org/10.3389/fpubh.2023.1156782

3 Dube, W. C. *et al.* Quantifying Risk for SARS-CoV-2 Infection Among Nursing Home Workers for the 2020-2021 Winter Surge of the COVID-19 Pandemic in Georgia, USA. *J Am Med Dir Assoc* **23**, 942-946 e941 (2022). https://doi.org/10.1016/j.jamda.2022.02.014

4 Allen, N. *et al.* Prevalence of Antibodies to SARS-CoV-2 Following Natural Infection and Vaccination in Irish Hospital Healthcare Workers: Changing Epidemiology as the Pandemic Progresses. *Front Med (Lausanne)* **8**, 758118 (2021). https://doi.org/10.3389/fmed.2021.758118

5 Martin, C. A. *et al.* Risk factors associated with SARS-CoV-2 infection in a multiethnic cohort of United Kingdom healthcare workers (UK-REACH): A cross-sectional analysis. *PLoS Med* **19**, e1004015 (2022). https://doi.org/10.1371/journal.pmed.1004015

6 Maina, D. *et al.* Seroprevalence, correlates and kinetics of SARS-CoV-2 nucleocapsid IgG antibody in healthcare workers and nonclinical staff at a tertiary hospital: A prevaccine census study. *PLoS One* **17**, e0267619 (2022). https://doi.org/10.1371/journal.pone.0267619

7 Zoltowska, B. *et al.* The Seroprevalence of SARS-CoV-2 Antibodies among HealthCare Workers in University Hospital in Krakow before the Era of Vaccination. *Int J Environ Res Public Health* **19** (2022). https://doi.org/10.3390/ijerph19074044

8 Bueno-Hernandez, N. *et al.* High Incidence Rate of SARS-CoV-2 Infection in Health Care Workers at a Dedicated COVID-19 Hospital: Experiences of the Pandemic from a Large Mexican Hospital. *Healthcare (Basel)* **10** (2022). https://doi.org/10.3390/healthcare10050896

9 Doernberg, S. B. *et al.* Incidence and prevalence of COVID-19 within a healthcare worker cohort during the first year of the SARS-CoV-2 pandemic. *Clin Infect Dis* (2022). https://doi.org/10.1093/cid/ciac210

10 Stephen, R. I., Olumoh, J., Tyndall, J. & Adegboye, O. Risk Factors for COVID-19 Infection among Healthcare Workers in North-East Nigeria. *Healthcare (Basel)* **10** (2022). https://doi.org/10.3390/healthcare10101919

11 Bansal, A. *et al.* Risk assessment and antibody responses to SARS-CoV-2 in healthcare workers. *Front. Public Health* **11** (2023). https://doi.org/10.3389/fpubh.2023.1164326

12 Chea, N. *et al.* Risk Factors for SARS-CoV-2 Infection Among US Healthcare Personnel, May-December 2020. *Emerg Infect Dis* **28**, 95-103 (2022). https://doi.org/10.3201/eid2801.211803

13 Socan, M. *et al.* Determinants of seropositivity for SARS-CoV-2 in hospital staff in the second wave of the pandemic in Slovenia. *Int J Occup Med Environ Health* **35**, 571-584 (2022). https://doi.org/10.13075/ijomeh.1896.01932

14 Rosinska, M. *et al.* Healthcare workers highly affected during the COVID-19 epidemic wave in Poland prior to vaccination availability: seroprevalence study. *Med Pr* **73**, 109-123 (2022). https://doi.org/10.13075/mp.5893.01216

15 Ghosh, S. *et al.* Seropositivity of severe acute respiratory syndrome coronavirus 2 infection among healthcare workers of the Armed Forces medical services, India: A multicentric study. *Med J Armed Forces India* **77**, S359-S365 (2021). https://doi.org/10.1016/j.mjafi.2021.03.020

16 Allen, N. *et al.* Prevalence of antibodies to SARS-CoV-2 in Irish hospital healthcare workers. *Epidemiol Infect* **149**, e157 (2021). https://doi.org/10.1017/S0950268821000984

17 Sharma, P. *et al.* Seroprevalence of antibodies to SARS-CoV-2 and predictors of seropositivity among employees of a teaching hospital in New Delhi, India. *Osong Public Health Res Perspect* **12**, 88-95 (2021). https://doi.org/10.24171/j.phrp.2021.12.2.06
18 Brousseau, N. *et al.* SARS-CoV-2 seroprevalence in health care workers from 10 hospitals in Quebec, Canada: a cross-sectional study. *CMAJ* **193**, E1868-E1877 (2021). https://doi.org/10.1503/cmaj.202783
19 Akinbami, L. J. *et al.* Severe Acute Respiratory Syndrome Coronavirus 2 Seropositivity among Healthcare Personnel in Hospitals and Nursing Homes, Rhode Island, USA, July-August 2020. *Emerg Infect Dis* **27**, 823-834 (2021). https://doi.org/10.3201/eid2703.204508
20 Goenka, M. *et al.* Seroprevalence of COVID-19 Amongst Health Care Workers in a Tertiary Care Hospital of a Metropolitan City from India. *J Assoc Physicians India* **68**, 14-19 (2020).
21 Szajek, K. *et al.* Healthcare institutions' recommendation regarding the use of FFP-2 masks and SARS-CoV-2 seropositivity among healthcare workers: a multicenter longitudinal cohort study. *Antimicrob Resist Infect Control* **11**, 6 (2022). https://doi.org/10.1186/s13756-021-01047-x
22 Kahlert, C. R. *et al.* Non-occupational and occupational factors associated with specific SARS-CoV-2 antibodies among hospital workers - A multicentre cross-sectional study. *Clin Microbiol Infect* **27**, 1336-1344 (2021). https://doi.org/10.1016/j.cmi.2021.05.014
23 Ludewick, H. *et al.* COVID-19 Serosurvey of Frontline Healthcare Workers in Western Australia. *J Epidemiol Glob Health* (2022). https://doi.org/10.1007/s44197-022-00065-1
24 Papasavas, P. *et al.* Seroprevalence of SARS-CoV-2 antibodies, associated epidemiological factors and antibody kinetics among healthcare workers in Connecticut. *J Hosp Infect* **114**, 117-125 (2021). https://doi.org/10.1016/j.jhin.2021.04.021
25 Jacob, J. T. *et al.* Risk Factors Associated With SARS-CoV-2 Seropositivity Among US Health Care Personnel. *JAMA Netw Open* **4**, e211283 (2021). https://doi.org/10.1001/jamanetworkopen.2021.1283
26 Gupta, R. *et al.* Seroprevalence of antibodies to SARS-CoV-2 in healthcare workers & implications of infection control practice in India. *Indian J Med Res* **153**, 207-213 (2021). https://doi.org/10.4103/ijmr.IJMR_3911_20
27 Paris, C. *et al.* Risk factors for SARS-CoV-2 infection among health care workers. *Am J Infect Control* **50**, 375-382 (2022). https://doi.org/10.1016/j.ajic.2021.11.001
28 Dev, N., Meena, R. C., Gupta, D. K., Gupta, N. & Sankar, J. Risk factors and frequency of COVID-19 among healthcare workers at a tertiary care centre in India: a case-control study. *Trans R Soc Trop Med Hyg* **115**, 551-556 (2021). https://doi.org/10.1093/trstmh/trab047
29 Rodriguez-Lopez, M. *et al.* A case-control study of factors associated with SARS-CoV-2 infection among healthcare workers in Colombia. *BMC Infect Dis* **21**, 878 (2021). https://doi.org/10.1186/s12879-021-06581-y
30 Mutha, A. S. *et al.* Risk Factors for Reverse Transcriptase Polymerase Chain Reaction Positivity for SARS-CoV-2 among Healthcare Workers in a Group of Tertiary Care Hospitals in Mumbai: A Cross-sectional Study. *JCDR* **15** (2021). https://doi.org/10.7860/JCDR/2021/48855.14834
31 Nishida, T. *et al.* Seroprevalence of SARS-CoV-2 antibodies among 925 staff members in an urban hospital accepting COVID-19 patients in Osaka prefecture, Japan: A cross-sectional study. *Medicine (Baltimore)* **100**, e26433 (2021). https://doi.org/10.1097/MD.0000000000026433
32 Howard-Anderson, J. R. *et al.* Occupational risk factors for severe acute respiratory coronavirus virus 2 (SARS-CoV-2) infection among healthcare personnel: A cross-sectional analysis of subjects enrolled in the COVID-19 Prevention in Emory Healthcare Personnel (COPE) study. *Infect Control Hosp Epidemiol* **43**, 381-386 (2022). https://doi.org/10.1017/ice.2021.54
33 Brant-Zawadzki, M. *et al.* SARS-CoV-2 antibody prevalence in health care workers: Preliminary report of a single center study. *PLoS One* **15**, e0240006 (2020). https://doi.org/10.1371/journal.pone.0240006

34 Blairon, L. *et al.* Large-scale, molecular and serological SARS-CoV-2 screening of healthcare workers in a 4-site public hospital in Belgium after COVID-19 outbreak. *J Infect* **82**, 159-198 (2021). https://doi.org/10.1016/j.jinf.2020.07.033
35 Wilkins, J. T. *et al.* Seroprevalence and Correlates of SARS-CoV-2 Antibodies in Health Care Workers in Chicago. *Open Forum Infect Dis* **8**, ofaa582 (2021). https://doi.org/10.1093/ofid/ofaa582
36 Bryan, A. *et al.* Cross-sectional study evaluating the seroprevalence of SARS-CoV-2 antibodies among healthcare workers and factors associated with exposure during the first wave of the COVID-19 pandemic in New York. *BMJ Open* **11**, e053158 (2021). https://doi.org/10.1136/bmjopen-2021-053158
37 Baker, J. M. *et al.* Quantification of Occupational and Community Risk Factors for SARS-CoV-2 Seropositivity Among Health Care Workers in a Large U.S. Health Care System. *Ann Intern Med* **174**, 649-654 (2021). https://doi.org/10.7326/M20-7145
38 Moscola, J. *et al.* Prevalence of SARS-CoV-2 Antibodies in Health Care Personnel in the New York City Area. *JAMA* (2020). https://doi.org/10.1001/jama.2020.14765
39 Darvishian, M. *et al.* SARS-CoV-2 Seroprevalence Among Health Care Workers in Major Private and Public Hospitals With COVID-19 Patient's Referral in Tehran, Iran. *Front Public Health* **10**, 832003 (2022). https://doi.org/10.3389/fpubh.2022.832003
40 Trieu, M. C. *et al.* SARS-CoV-2-Specific Neutralizing Antibody Responses in Norwegian Health Care Workers After the First Wave of COVID-19 Pandemic: A Prospective Cohort Study. *J Infect Dis* **223**, 589-599 (2021). https://doi.org/10.1093/infdis/jiaa737
41 Eyre, D. W. *et al.* Differential occupational risks to healthcare workers from SARS-CoV-2 observed during a prospective observational study. *Elife* **9** (2020). https://doi.org/10.7554/eLife.60675
42 Venugopal, U. *et al.* SARS-CoV-2 seroprevalence among health care workers in a New York City hospital: A cross-sectional analysis during the COVID-19 pandemic. *Int J Infect Dis* **102**, 63-69 (2021). https://doi.org/10.1016/j.ijid.2020.10.036
43 Celebi, G. *et al.* Specific risk factors for SARS-CoV-2 transmission among health care workers in a university hospital. *Am J Infect Control* **48**, 1225-1230 (2020). https://doi.org/10.1016/j.ajic.2020.07.039
44 Rosser, J. I. *et al.* Severe acute respiratory coronavirus virus 2 (SARS-CoV-2) seroprevalence in healthcare personnel in northern California early in the coronavirus disease 2019 (COVID-19) pandemic. *Infect Control Hosp Epidemiol* **42**, 1053-1059 (2021). https://doi.org/10.1017/ice.2020.1358
45 Rudberg, A. S. *et al.* SARS-CoV-2 exposure, symptoms and seroprevalence in healthcare workers in Sweden. *Nat Commun* **11**, 5064 (2020). https://doi.org/10.1038/s41467-020-18848-0
46 Iversen, K. *et al.* Risk of COVID-19 in health-care workers in Denmark: an observational cohort study. *Lancet Infect Dis* **20**, 1401-1408 (2020). https://doi.org/10.1016/S1473-3099(20)30589-2
47 Piccoli, L. *et al.* Risk assessment and seroprevalence of SARS-CoV-2 infection in healthcare workers of COVID-19 and non-COVID-19 hospitals in Southern Switzerland. *Lancet Reg Health Eur* **1**, 100013 (2021). https://doi.org/10.1016/j.lanepe.2020.100013
48 Steensels, D. *et al.* Hospital-Wide SARS-CoV-2 Antibody Screening in 3056 Staff in a Tertiary Center in Belgium. *JAMA* **324**, 195-197 (2020). https://doi.org/10.1001/jama.2020.11160
49 Galan, M. I. *et al.* Hospital-Wide SARS-CoV-2 seroprevalence in health care workers in a Spanish teaching hospital. *Enferm Infecc Microbiol Clin (Engl Ed)* (2020). https://doi.org/10.1016/j.eimc.2020.11.015
50 Sotgiu, G. *et al.* SARS-CoV-2 specific serological pattern in healthcare workers of an Italian COVID-19 forefront hospital. *BMC Pulm Med* **20**, 203 (2020). https://doi.org/10.1186/s12890-020-01237-0

51 Garcia-Basteiro, A. L. *et al.* Seroprevalence of antibodies against SARS-CoV-2 among health care workers in a large Spanish reference hospital. *Nat Commun* **11**, 3500 (2020). https://doi.org/10.1038/s41467-020-17318-x
52 Kohler, P. P. *et al.* Prevalence of SARS-CoV-2 antibodies among Swiss hospital workers: Results of a prospective cohort study. *Infect Control Hosp Epidemiol* **42**, 604-608 (2021). https://doi.org/10.1017/ice.2020.1244
53 Algado-Selles, N. *et al.* Frequency, Associated Risk Factors, and Characteristics of COVID-19 Among Healthcare Personnel in a Spanish Health Department. *Am J Prev Med* **59**, e221-e229 (2020). https://doi.org/10.1016/j.amepre.2020.07.014
54 Fusco, F. M. *et al.* COVID-19 among healthcare workers in a specialist infectious diseases setting in Naples, Southern Italy: results of a cross-sectional surveillance study. *J Hosp Infect* **105**, 596-600 (2020). https://doi.org/10.1016/j.jhin.2020.06.021
55 Ran, L. *et al.* Risk Factors of Healthcare Workers With Coronavirus Disease 2019: A Retrospective Cohort Study in a Designated Hospital of Wuhan in China. *Clin Infect Dis* **71**, 2218-2221 (2020). https://doi.org/10.1093/cid/ciaa287
56 Wang, X. *et al.* Risk factors of SARS-CoV-2 infection in healthcare workers: a retrospective study of a nosocomial outbreak. *Sleep Med X* **2**, 100028 (2020). https://doi.org/10.1016/j.sleepx.2020.100028
57 Lartey, M. *et al.* Risk factors for COVID-19 infections among health care workers in Ghana. *PLoS One* **18**, e0288242 (2023). https://doi.org/10.1371/journal.pone.0288242
58 Mukwege, D. *et al.* High SARS-CoV-2 Seroprevalence in Healthcare Workers in Bukavu, Eastern Democratic Republic of Congo. *Am J Trop Med Hyg* **104**, 1526-1530 (2021). https://doi.org/10.4269/ajtmh.20-1526
59 Atnafie, S. A., Anteneh, D. A., Yimenu, D. K. & Kifle, Z. D. Assessment of exposure risks to COVID-19 among frontline health care workers in Amhara Region, Ethiopia: A cross-sectional survey. *PLoS One* **16**, e0251000 (2021). https://doi.org/10.1371/journal.pone.0251000
60 Kataria, Y. *et al.* Seroprevalence of SARS-CoV-2 IgG antibodies and risk factors in health care workers at an academic medical center in Boston, Massachusetts. *Sci Rep* **11**, 9694 (2021). https://doi.org/10.1038/s41598-021-89107-5
61 Brehm, T. T. *et al.* Seroprevalence of SARS-CoV-2 antibodies among hospital workers in a German tertiary care center: A sequential follow-up study. *Int J Hyg Environ Health* **232**, 113671 (2021). https://doi.org/10.1016/j.ijheh.2020.113671
62 Chatterjee, P. *et al.* Healthcare workers & SARS-CoV-2 infection in India: A case-control investigation in the time of COVID-19. *Indian J Med Res* **151**, 459-467 (2020). https://doi.org/10.4103/ijmr.IJMR_2234_20
63 Lau, J. S. *et al.* SARS-CoV-2 seroprevalence in healthcare workers in a tertiary healthcare network in Victoria, Australia. *Infect Dis Health* **26**, 208-213 (2021). https://doi.org/10.1016/j.idh.2021.03.004
